# Prediction of oncogene mutation status in non-small cell lung cancer: a systematic review and meta-analysis with a special focus on artificial intelligence-based methods

**DOI:** 10.1007/s00330-025-11962-x

**Published:** 2025-09-08

**Authors:** Almudena Fuster-Matanzo, Alfonso Picó-Peris, Fuensanta Bellvís-Bataller, Ana Jimenez-Pastor, Glen J. Weiss, Luis Martí-Bonmatí, Antonio Lázaro Sánchez, David Bazaga, Giuseppe L. Banna, Alfredo Addeo, Carlos Camps, Luis M. Seijo, Ángel Alberich-Bayarri

**Affiliations:** 1 Quantitative Imaging Biomarkers in Medicine, Quibim, Valencia, Spain; 2 Quantitative Imaging Biomarkers in Medicine, Quibim, New York, NY USA; 3 Grupo de Investigación Biomédica en Imagen, Instituto de Investigación Sanitaria La Fe, Valencia, Spain; 4 Área Clínica de Imagen Médica, Hospital Universitari i Politècnic La Fe, València, Spain; 5 Department of Medical Oncology, Hospital General Universitario Morales Meseguer, Murcia, Spain; 6 Department of Oncology, Portsmouth Hospitals University NHS Trust, Portsmouth, UK; 7 Faculty of Science and Health, School of Pharmacy and Biomedical Sciences, University of Portsmouth, Portsmouth, UK; 8 Oncology Service, University Hospital Geneva, Geneva, Switzerland; 9 Department of Medicine, University of Valencia, Valencia, Spain; 10 Unidad Mixta Ómica, Centro Investigación Príncipe Felipe-Universidad de Valencia, Valencia, Spain; 11 Clínica Universidad de Navarra, Madrid, Spain

**Keywords:** Radiomics, Artificial intelligence, Diagnostic imaging, Mutation, Non-small cell lung cancer.

## Abstract

**Objectives:**

In non-small cell lung cancer (NSCLC), non-invasive alternatives to biopsy-dependent driver mutation analysis are needed. We reviewed the effectiveness of radiomics alone or with clinical data and assessed the performance of artificial intelligence (AI) models in predicting oncogene mutation status.

**Materials and methods:**

A PRISMA-compliant literature review for studies predicting oncogene mutation status in NSCLC patients using radiomics was conducted by a multidisciplinary team. Meta-analyses evaluating the performance of AI-based models developed with CT-derived radiomics features alone or combined with clinical data were performed. A meta-regression to analyze the influence of different predictors was also conducted.

**Results:**

Of 890 studies identified, 124 evaluating models for the prediction of epidermal growth factor-1 (EGFR), anaplastic lymphoma kinase (ALK), and Kirsten rat sarcoma virus (KRAS) mutations were included in the systematic review, of which 51 were meta-analyzed. The AI algorithms’ sensitivity/false positive rate (FPR) in predicting mutation status using radiomics-based models was 0.754 (95% CI 0.727–0.780)/0.344 (95% CI 0.308–0.381) for EGFR, 0.754 (95% CI 0.638–0.841)/0.225 (95% CI 0.163–0.302) for ALK and 0.475 (95% CI 0.153–0.820)/0.181 (95% CI 0.054–0.461) for KRAS. A meta-analysis of combined models was possible for EGFR mutation, revealing a sensitivity of 0.806 (95% CI 0.777–0.833) and a FPR of 0.315 (95% CI 0.270–0.364). No statistically significant results were obtained in the meta-regression.

**Conclusions:**

Radiomics-based models may offer a non-invasive alternative for determining oncogene mutation status in NSCLC. Further research is required to analyze whether clinical data might boost their performance.

**Key Points:**

***Question*** *Can imaging-based radiomics and artificial intelligence non-invasively predict oncogene mutation status to improve diagnosis in non-small cell lung cancer (NSCLC)?*

***Findings*** *Radiomics-based models achieved high performance in predicting mutation status in NSCLC; adding clinical data showed limited improvement in predictive performance.*

***Clinical relevance*** *Radiomics and AI tools offer a non-invasive strategy to support molecular profiling in NSCLC. Validation studies addressing clinical and methodological aspects are essential to ensure their reliability and integration into routine clinical practice.*

**Graphical Abstract:**

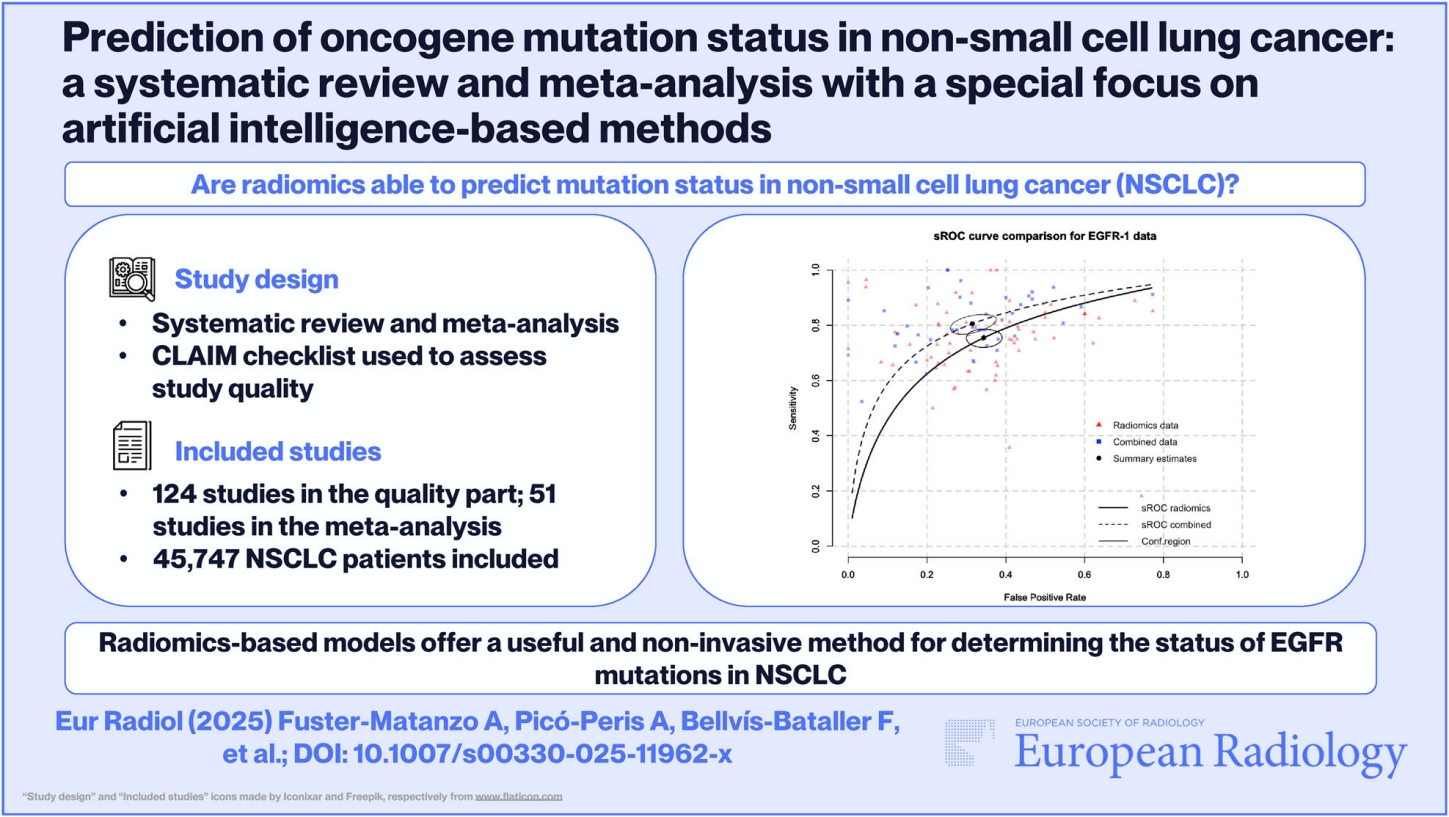

## Introduction

Lung cancer represents the most lethal cancer in both women and men worldwide [1]. Non-small cell lung cancer (NSCLC), the most frequent histological subtype, accounts for 80–85% of cases, with adenocarcinoma being the most common subtype (40–50% of cases) [2]. Molecular subtyping has become crucial for genotype-driven targeted therapy, now being the standard of care for many patients with advanced NSCLC [3]. However, conventional methods for molecular genotyping require invasive testing and genetic sequence testing, facing challenges such as high costs, sampling bias, sample adequacy, turnaround time, procedural risks and medical complications [4–6]. Furthermore, the overall accessibility of alternative blood-based molecular diagnostics, known as liquid biopsy, may be limited for many patients [7], highlighting the need to investigate non-invasive methods to characterize the oncogene mutation status of NSCLC.

Radiological imaging is a crucial non-invasive diagnostic tool for lung cancer, used in screening, diagnosis, staging, and the management of early- and advanced-stage cases [8]. Specifically, computed tomography (CT) remains the standard of care for lung cancer visualization, providing excellent morphological and textural information. In recent years, radiomics, a scientific discipline focused on the extraction and analysis of quantitative features from medical images, has gained attention, especially in lung cancer [8]. Furthermore, the application of artificial intelligence (AI) to imaging has enabled addressing important clinical needs by predicting outcomes, treatment response, disease progression, or the mutational and molecular profiling of tumors [9]. Particularly, the combination of radiomics and AI has proven to be a promising non-invasive tool for predicting oncogene mutation status in NSCLC [8].

In this systematic review and meta-analysis our multidisciplinary group aimed to: (1) review the available scientific evidence on the use of imaging-based models and radiomics for the prediction of three key targetable oncogenic driver alterations in NSCLC, including epidermal growth factor receptor (EGFR), anaplastic lymphoma kinase (ALK), and Kirsten rat sarcoma virus (KRAS); (2) analyze the overall performance of AI-based methods specifically for the prediction of oncogene mutation status; (3) evaluate whether the inclusion of clinical variables in the models improve their performance; and (4) evaluate the impact of the available evidence from a clinical perspective.

## Materials and methods

This systematic review and meta-analysis were conducted in accordance with the Preferred Reporting Items for Systematic Reviews and Meta-Analysis (PRISMA) [10] and Meta-analyses Of Observational Studies in Epidemiology (MOOSE) [11] guidelines. The review was registered on PROSPERO before initiation (registration no. CRD42022349809; Supplementary Materials).

### Search strategy

A systematic search for eligible publications published through 20 November 2024 was performed in Medline (via Pubmed), Cochrane Library and EMBASE databases using the keywords “Radiomics,” “NSCLC” and “Mutational status” (further details provided in Supplementary Table S1). There were no limitations on the publishing year, participant age, or nationality. The search was exclusively limited to English-language publications.

### Study selection

Literature search and study selection were independently performed by two reviewers. Covidence systematic review software (Veritas Health Innovation, Melbourne, Australia; www.covidence.org) was used as a screening and data extraction tool (inclusion and exclusion criteria in Supplementary Materials).

### Quality assessment

The methodological quality of each study for its possible inclusion in the quantitative assessment was evaluated by using the Checklist for Artificial Intelligence in Medical Imaging (CLAIM) [12] (see Supplementary Materials). For the studies included, the risk of bias (RoB) was subsequently assessed using the Quality Assessment of Diagnostic Accuracy Studies-2 (QUADAS-2) tool [13], which is specifically designed for the evaluation of diagnostic accuracy studies. The tool was applied across its four standard domains—patient selection, index test, reference standard, and flow and timing—without modifications (see Supplementary Materials).

### Data extraction

See Supplementary Materials.

### Data analysis

For quantitative analysis, only CT scan-based models were considered. Meta-analyses were performed using a bivariate approach for sensitivity and false positive rate (FPR), following Reitsma et al’s method [14]. A summary receiver operating characteristic (sROC) was calculated, converting each sensitivity and specificity pair into a diagnostic odds ratio (DOR), which was used as the primary summary metric due to its robustness across varying thresholds and its clinical interpretability. The DOR is defined as (sensitivity × specificity) / [(1 − sensitivity) × (1 − specificity)] and reflects the odds of a positive result in patients with the target condition relative to those without it. A DOR of 1 indicates no discriminative ability, while higher values denote improved diagnostic performance. Importantly, the DOR is independent of disease prevalence, which makes it particularly suitable for meta-analyses pooling data across studies with varying population characteristics. FPR, mathematically equivalent to 1−specificity, was deliberately selected instead of specificity, as it more directly reflects the likelihood of incorrect positive predictions—a key concern in clinical decision-making scenarios. Additional details are available in Supplementary Materials.

To assess the risk of publication bias across studies, we performed Deeks’ funnel plot asymmetry test for each mutation type (EGFR, ALK, KRAS, and combined EGFR models), as recommended for diagnostic test accuracy meta-analyses. A *p*-value < 0.10 was considered indicative of significant asymmetry.

A meta-regression, exploring the influence of different predictors, was also performed (see Supplementary Materials).

Analyses were conducted with R Statistical Software v4.2.2 and mada/tidyverse packages.

## Results

**Fig. 1:**
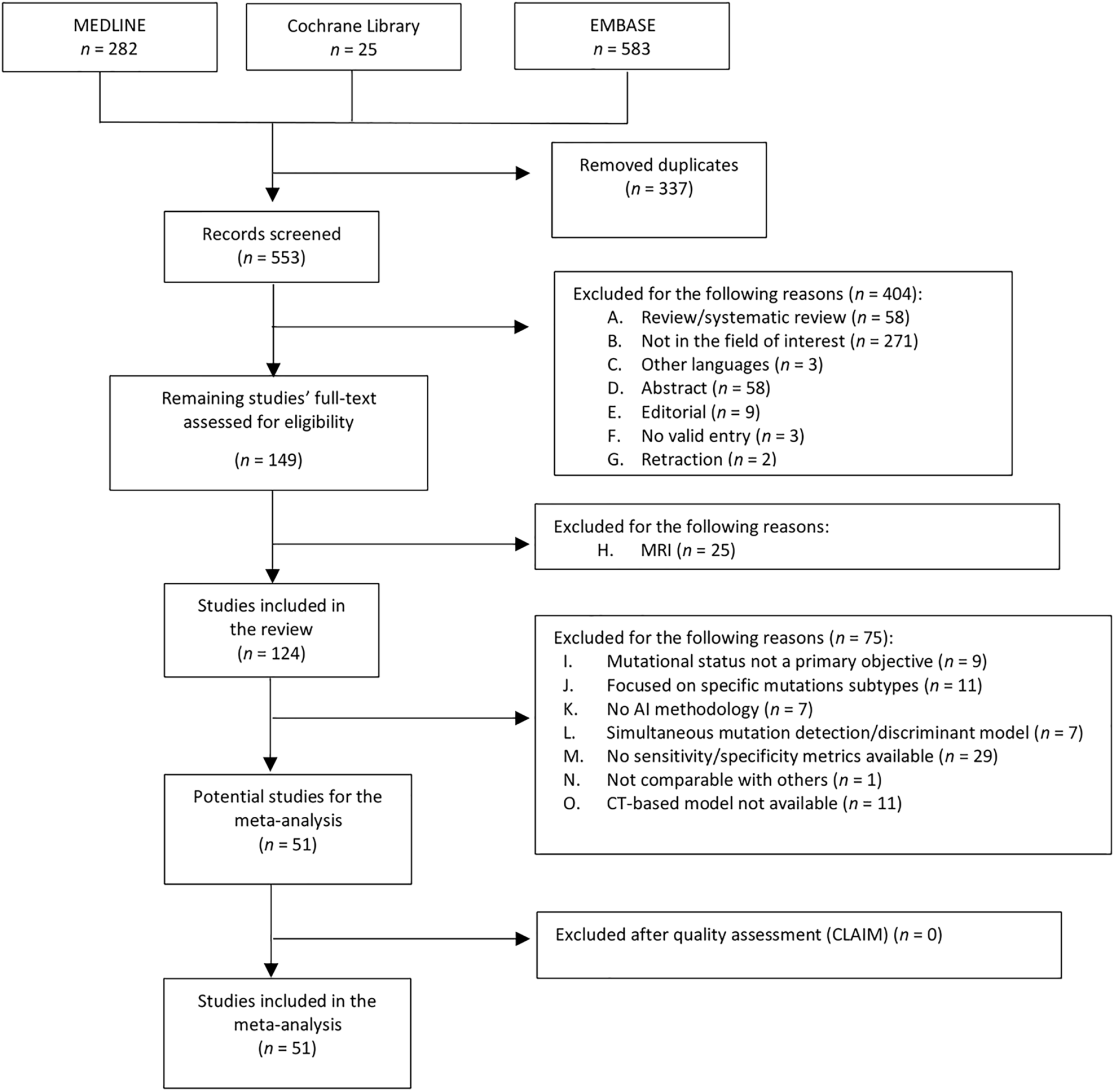
PRISMA flowchart. AI, artificial intelligence; CLAIM, Checklist for Artificial Intelligence in Medical Imaging; CT, computed tomography; MRI, magnetic resonance imaging

A total of 890 articles were obtained according to the search strategy (Fig. 1). After de-duplication, 553 studies were selected and screened. According to the inclusion and exclusion criteria, 124 studies were included in the systematic review, all of them developing models for the prediction of EGFR, ALK, and/or KRAS. Out of those, 51 were found eligible for meta-analyses, passed the quality check, and were therefore included (Supplementary Table S2).

### Qualitative analysis (systematic review)

#### Methodological characteristics of the studies

Table 1 summarizes the methodological characteristics of the studies. Out of 124 studies, 90 exclusively used machine learning (ML) algorithms, and 14 employed ML for comparator models in deep learning (DL)-focused articles. Only 10 studies exclusively used DL algorithms, while classical statistical models were used in 10 publications. Among the 90 articles using ML (one or more classifiers), the most common classifiers were logistic regression (*n* = 56), support vector machine (*n* = 49), and random forest (*n* = 39). The most frequently used partitioning strategy among the 124 papers analyzed was the training-validation split (*n* = 98). External validation was only performed in a small set of studies (*n* = 21). Regarding imaging techniques, CT was the most frequently used modality (*n* = 86), followed by positron emission tomography (PET)/CT (*n* = 30) and by PET alone (*n* = 6). Additionally, in one study [15], PET/CT scans and contrast-enhanced CT images independently acquired were collected, while in another study [16], PET/CT, CT, and contrast-enhanced diagnostic quality (CTD) images were used. Of the 86 studies conducted with CT scans, 51 included non-contrast-enhanced images and 23 contrast-enhanced images. Eight studies did not specify the use of contrast, while four included contrast- and non-contrast-enhanced scans. Manual segmentation was performed in 73 studies, and automatic or semi-automatic segmentation was used in four and 38 studies, respectively. Three studies applied both methodologies (for verification or a different approach according to the imaging modality used), and six studies did not specify the method of segmentation.

**Table 1:** Methodological characteristics of the studies (*N* = 124) included in the systematic review

Author [ref]	Imaging modality	Contrast CT*	Tumor segmentation	Model	Classifier (ML)	Datasets			Partition strategy
						Training	Validation	Test	
Agüloğlu et al [51]	PET/CT	Non-contrast CT	Semi-automatic	ML	RF NB KNN DT SVM LR	133	56	–	Training-Validation split
Aerts et al [40]	CT	Non-contrast CT	Semi-automatic	Classical statistical model	–	–	–	–	–
Agazzi et al [123]	CT	Contrast-enhanced	Manual	ML	GBM	104	67	–	Training-Validation split
Aide et al [36]	PET	–	Manual	ML	LASSO	87	22	–	Training-Validation split
Chang et al [82]	PET/CT	Non-contrast CT	Manual	ML	LASSO	409	174	–	Training-Validation split
Chang et al [104]	PET/CT	Non-contrast CT	Manual	ML	LR^†^	367	159	–	Training-Validation split
Chen et al [124]	CT	Non-contrast CT	Manual	ML	SVM	179	44	–	Training-Validation split
Chen et al [125]	CT	Non-contrast CT	Semi-automatic	ML	LASSO	176	57	–	Training-Validation split
Chen et al [126]	CT	Non-contrast CT	Semi-automatic	ML	LASSO	678 246	203 123	164 95	Training-Validation split^‡^
Cheng et al [32]	CT	Non-contrast CT	Manual	ML	LR	135	68	–	Training-Validation split
Choe et al [127]	CT	Contrast-enhanced	Semi-automatic	ML	LR	349	154	–	Training-Validation split
Dang et al [17]	CT	Non-contrast CT	Semi-automatic	ML	LASSO	88	30	–	Training-Validation split
Digumarthy et al [128]	CT	Contrast-enhanced	Not specified	Classical statistical model	–	–	–	–	–
Dong et al [37]	CT	Non-contrast CT	Not specified	ML	LR	87	45	–	Training-Validation split
Dong et al [88]	CT	Non-contrast CT	Manual	DL ML	RF	363	162	–	Training-Validation split
Feng et al [89]	CT	Non-contrast CT	Manual	ML	RF XGBoost LR SVM	151	–	–	Training-Validation split
Gao et al [90]	PET/CT	Non-contrast CT	Semi-automatic	ML	LR RF SVM	404	111	–	Training-Validation split
Gong et al [91]	CT	Not specified	Manual	ML DL	SVM	654	164	131	Training-Validation split
Hao et al [129]	CT	Non-contrast CT	Manual	ML	SVM XGBoost AdaBoost LBP DT LR	154	39	–	Training-Validation split
He et al [130]	CT	Non-contrast CT	Semi-automatic	ML	RF KNN LGBM SVM	–	–	–	Training-Validation split
Hinzpeter et al [92]	PET/CT	Non-contrast CT	Semi-automatic	ML	LR	–	–	–	100-fold cross-validation
Hong et al [24]	CT	Contrast-enhanced	Manual	ML	NBC KNN RF SVM DT LR	140	61	–	Training-Validation split
Hu et al [33]	CT	Contrast-enhanced	Manual	ML	SVM RF Neural Network LR	–	–	69	Training-Validation split^†^
Huang et al [41]	CT	Non-contrast CT	Semi-automatic	Classical statistical model	–	–	–	–	–
Huang et al [131]	PET/CT	Non-contrast CT	Manual	DL ML	LR	138	57	–	Training-Validation split
Huang et al [120]	CT	Non-contrast CT	Manual	DL ML	LR	770	304	–	Training-Validation split
Huo et al [93]	CT	Contrast-enhanced	Manual	ML	GBT	487	121	–	Training-Validation split
Hou et al [132]	CT	Contrast-enhanced	Semi-automatic	Classical statistical model	–	144	62	–	Training-Validation split
Jia et al [94]	CT	Non-contrast CT	Semi-automatic	ML	RF	345	158	–	Training-Validation split
Jiang et al [133]	PET/CT	Non-contrast CT	Semi-automatic	ML	SVM	–	–	–	10-fold cross-validation
Jiang et al [95]	CT	Non-contrast CT	Manual	ML	SVM	514	178	–	Training-Validation split
Kawazoe et al [134]	CT	Non-contrast CT	Semi-automatic	ML	SVM LR LGBM	120	44	–	Training-Validation split
Kawazoe et al [135]	CT	Non-contrast CT	Semi-automatic	ML	SVM LR	120	52	–	Training-Validation split
Kim et al [136]	CT	Not specified	Automatic	DL	–	–	–	433	5-fold cross-validation
Kohan et al [96]	CT	Contrast-enhanced	Semi-automatic	ML	LR	–	–	–	100-fold cross-validation
Koyasu et al [137]	PET/CT	Non-contrast CT	Manual	ML	RF XGBoost	–	–	–	10-fold cross-validation
Le et al [52]	CT	Non-contrast CT	Manual	ML	XGBoost	143	18	–	Training-Validation split
Li et al [47]	CT	Non-contrast CT	Manual	DL ML	RF	810	200	–	Training-Validation split
Li et al [38]	CT	Contrast-enhanced	Semi-automatic	ML	SVM	–	–	–	3-fold cross-validation
Li et al [83]	PET/CT	Non-contrast CT	Manual	ML	Boosting ML scheme	–	–	–	10-fold cross-validation
Li et al [138]	CT	Non-contrast CT	Manual	ML	LR	236	76	–	Training-Validation split
Li et al [98]	CT	Non-contrast CT	Manual	ML	LR SVM RF NB Neural network	326	112	–	Training-Validation split
Li et al [139]	PET	–	Semi-automatic	ML	SVM	50	25	–	Training-Validation split
Li et al [97]	PET/CT	Non-contrast CT	Manual	ML	LR	125	54	–	Training-Validation split
Li et al [140]	CT	Non-contrast CT	Manual	ML	SVM LASSO	150	65	–	Training-Validation split
Li et al [141]	PET/CT	Non-contrast CT	Semi-automatic	ML	GBT	105	45	–	Training-Validation split
Liu et al [142]	CT	Non-contrast CT	Semi-automatic	Classical statistical model	–	–	–	–	–
Liu et al [84]	CT	Contrast-enhanced	Semi-automatic	ML	LR	210	53	–	Training-Validation split
Liu et al [21]	PET/CT	Non-contrast CT	Manual	ML	XGBoost	111	37	–	Training-Validation split
Liu et al [99]	CT	Non-contrast CT	Manual	ML	LR DT RF SVM	296	50	–	Training-Validation split
Liu et al [143]	PET/CT	Non-contrast CT	Manual	ML	LR DT RF SVM	–	–	–	10-fold cross-validation
Liu et al [144]	CT	Non-contrast CT	Manual	Classical statistical model	–	–	–	–	–
Lu et al [85]	CT	Non-contrast CT	Manual	ML	LR	–	–	21	5-fold cross-validation
Lu et al [145]	CT	Non-contrast CT	Semi-automatic	ML	KNN Bagging SVM RF	105	228	–	Training-Validation split
Lu et al [26]	CT	Non-contrast CT	Manual	ML	DT AdaBoost NB RF LR SVM XGBoost KNN	140	61	–	Training-Validation split
Lu et al [25]	CT	Non-contrast CT	Manual	ML	DT KNN LR NB RF SVM XGBoost	192	82	–	Training-Validation split
Ma et al [105]	CT	Contrast-enhanced	Manual	ML	SVM	98	42	–	Training-Validation split
Ma et al [100]	CT	Contrast-enhanced	Manual	ML	LR RF SVM AdaBoost	122	53	–	Training-Validation split
Mahajan et al [27]	CT	Contrast-enhanced	Semi-automatic	ML DL	SVM	–	–	–	3-fold cross-validation
Mei et al [146]	CT	Non-contrast CT	Manual	Classical statistical model	–	–	–	–	–
Mu et al [121]	PET/CT	Non-contrast CT	Manual	DL	–	429	187	65	Training-Validation split
Nair et al [15]	PET/CT	Contrast-enhanced	Manual	ML	LR	–	–	–	LOOCV
Ninomiya et al [44]	CT	Contrast-enhanced	Manual	ML	SVM	–	–	95	5-fold cross-validation
Ninomiya et al [53]	CT	Contrast-enhanced	Not specified	ML	SVM	92	62	–	Training-Validation split
Njoto et al [43]	CT	Contrast-enhanced	Not specified	Classical statistical model	–	–	–	48	–
Omura et al [18]	CT	Contrast-enhanced	Automatic	ML	RF	–	–	–	Training-Validation split
Ottaiano et al [54]	CT	Not specified	Manual	ML	SVM	–	–	–	Cross-validation
Rinaldi et al [55]	CT	Contrast-enhanced	Manual	ML	LASSO	261	48	–	Training-Validation split
Ríos Velázquez et al [56]	CT	Contrast + Non-contrast CT	Semi-automatic	ML	RF	353	352	–	Training-Validation split
Rossi et al [101]	CT	Non-contrast CT	Manual	ML	SVM	–	–	61	5-fold cross-validation
Ruan et al [57]	PET/CT	Non-contrast CT	Manual	ML	SVM	70	30	–	Training-Validation split
Shang et al [58]	CT	Non-contrast CT	Manual	ML	RF KNN LR ExtraTrees XGBoost NeuralNetFastAI NeuralNetTorc LightGBM	384	128	139	Training-Validation split
Shao et al [59]	CT	Non-contrast CT	Semi-automatic	DL	–	–	–	–	Training-Validation split
Shao et al [60]	PET/CT	Non-contrast CT	Semi-automatic	DL	–	404	112	–	Training-Validation split
Shiri et al [16]	CT low-dose CTD PET/CT	Contrast-enhanced	Manual Automatic^§^	ML	SVM KNN DT QDA MLP SGD LR NB GNB RF AdaBoost Bagging	–	–	68	10-fold cross-validation
Shiri et al [61]	PET/CT	Non-contrast CT	Manual Automatic^§^	ML	RF	–	–	–	Training-Validation split
Song et al [62]	CT	Not specified	Manual Automatic	DL ML	SVM	528	137	–	Training-Validation split
Song et al [106]	CT	Non-contrast CT	Automatic	ML	DT	268	67	–	Training-Validation split
Tan et al [102]	PET/CT	Non-contrast CT	Manual	ML	LR	117	38	–	Training-Validation split
Trivizakis et al [63]	CT	Not specified	Not specified	DL ML	KNN DT RBF-GPC RBF-SVM Linear SVM Polynomial SVM Sigmoid SVM	–	–	–	5-fold cross-validation
Tu et al [64]	CT	Non-contrast CT	Not specified	ML	LR	243	161	–	Training-Validation split
Wang et al [19]	CT	Contrast-enhanced	Manual	ML	SVM	41	–	–	Training-Validation split
Wang et al [65]	CT	Non-contrast CT	Manual	DL	–	882	125	255	Training-Validation split
Wang et al [48]	CT	Non-contrast CT	Manual	DL ML	LASSO	–	–	–	Training-Validation split
Wang et al [45]	PET/CT	Non-contrast CT	Semi-automatic	ML	LR	180	78	–	Training-Validation split
Wang et al [66]	PET/CT	Non-contrast CT	Manual	ML	SVM	189	80	–	Training-Validation split
Wang et al [86]	CT	Non-contrast CT	Manual	DL	–	187	80	–	Training-Validation split
Wang et al [147]	PET	–	Semi-automatic	ML	LR	131 251 382	55 107 162	–	Training-Validation split^¶^
Wang et al [34]	PET/CT	Non-contrast CT	Manual	Classical statistical model	LR	–	*–*	–	–
Weng et al [67]	CT	Non-contrast CT	Semi-automatic	ML	LR	210	91	–	Training-Validation split
Wu et al [28]	CT	Contrast-enhanced	Manual	ML	LR	–	*–*	–	10-fold cross-validation
Wu et al [20]	CT	Not specified	Manual	DL	_	268	115	55	Training-Validation split
Xiao et al [68]	PET/CT	Non-contrast CT	Manual	DL ML	RF	121	29	–	Training-Validation split
Xiong et al [29]	CT	Non-contrast CT	Manual	ML	LR RF SVM	84	36	–	Training-Validation split
Xu et al [35]	CT	Contrast + Non-contrast CT	Manual	DL	–	339	146	–	Training-Validation split
Yamazaki et al [69]	CT	Non-contrast CT	Semi-automatic	ML	RF	–	–	–	–
Yang et al [23]	CT	Contrast-enhanced	Semi-automatic	ML	LASSO	130	40	–	Training-Validation split
Yang et al [70]	PET/CT	Non-contrast CT	Semi-automatic	ML	RF	139	35	–	Training-Validation split
Yang et al [49]	CT	Contrast + Non-contrast CT	Manual	ML	LR RF SVM GBT NB	327	66	19	Training-Validation split
Yang et al [22]	PET/CT	Non-contrast CT	Semi-automatic	ML	SVM DT RF	218	95	–	Training-Validation split
Yang et al [42]	CT	Contrast-enhanced	Manual	ML	LR	176	74	–	Training-Validation split
Yao et al [71]	PET/CT	Non-contrast CT	Manual	DL	–	103	44	–	Training-Validation split
Yip et al [72]	PET	–	Manual	Classical statistical model	–	–	–	–	–
Zhang et al [30]	CT	Non-contrast CT	Manual	ML	LR	140	40	–	Training-Validation split
Zhang et al [87]	PET/CT	Non-contrast CT	Manual	ML	RF SVM LR	–	–	–	10-fold cross-validation
Zhang et al [73]	PET/CT	Non-contrast CT	Semi-automatic	ML	LR	175	73	–	Training-Validation split
Zhang et al [46]	CT	Non-contrast CT	Semi-automatic	DL ML	RF SVM	638	71	205	Training-Validation split
Zhang et al [148]	CT	Contrast-enhanced	Semi-automatic	ML	LASSO	–	–	–	Training-Validation split
Zhang et al [103]	CT	Non-contrast CT	Manual	ML	DT LR SVM Multivariate analysis for C-R-R model	294	126	–	Training-Validation split
Zhang et al [149]	PET	–	Manual	ML	SVM RF LR AdaBoost	–	–	–	10-fold cross-validation
Zhang et al [74]	PET	–	Automatic	ML	LR	96	11	12	Training-Validation split
Zhang et al [50]	CT	Contrast + Non-contrast CT	Manual	ML	LR DT RF SVM KNN XGBoost	297	127	–	Training-Validation split
Zhang et al [81]	CT	Non-contrast CT	Manual	DL ML	LR SVM NB	528	132	–	Training-Validation split
Zhang et al [150]	CT	Contrast-enhanced	Manual	ML	SVM KNN RF NB LR MLP LDA	–	–	–	Training-Validation split^‡^
Zhang et al [75]	CT	Not specified	Manual	ML DL	NB MLP LDA SVM RF KNN LR	337 329 331	145 141 143	–	Training-Validation split^‡^
Zhao et al [122]	CT	Non-contrast CT	Manual	DL ML	LR	348	116	116	Training-Validation split
Zhao et al [78]	CT	Non-contrast CT	Manual	ML	LR	322	315	–	Training-Validation split
Zhao et al [76]	PET/CT	Non-contrast CT	Semi-automatic	ML	LR	65	23	–	Training-Validation split
Zhao et al [77]	CT	Not specified	Manual	DL	–	383	129	215	Training-Validation split
Zhu et al [39]	CT	Non-contrast CT	Semi-automatic	ML	LASSO RF SVM	875	217	–	Training-Validation split
Zhu et al [31]	CT	Contrast-enhanced	Manual	ML	SVM KNN RF LR	159	40	–	Training-Validation split
Zuo et al [80]	PET/CT	Non-contrast CT	Manual	ML	LGBM XGBoost RF LR	410	170	180	Training-Validation split
Zuo et al [79]	PET/CT	Non-contrast CT	Manual	ML	XGB LGBM CB GBT RF LR AdaBoost	265	65	60	Training-Validation split
Zuo et al [151]	PET/CT	Non-contrast CT	Manual	ML	LR RF LGBM XGB SVM GBT AdaBoost	312	78	88	Training-Validation split

For those studies with the same name for the first author and published the same year, a hashtag was added to unequivocally indicate those that were included in the different meta-analyses and consequently, that are represented in the forest plots

^*^In studies in which PET/CT was performed, only PET acquisition details were provided. Consequently, it was assumed that CT scans were non-contrast enhanced.

^†^Not specified but inferred from the methodology and results

^‡^Different partition strategies were performed according to the target gene (EGFR/KRAS/ALK [if applicable])

^§^Manual segmentation for PET images; automatic segmentation for CT images

^¶^Different partition strategies were performed according to the different objectives

*CB* cat-boost, *CT* computed tomography, *CTD* contrast-enhanced diagnostic quality, *DL* deep learning, *DT* decision tree, *ERBB2* v-erb-b2 avian erythroblastic leukemia viral oncogene homolog 2, *extraTree* extremely randomized trees, *GBM* gradient boosted machine, *GBT* gradient boosting tree, *GNB* Gaussian Naives Bayes, *GPC* Gaussian processes classification, *LASSO* least absolute shrinkage and selection operator, *LBP* local binary pattern, *LDA* linear discriminant analysis, *LGBM* Light gradient boosted machine, *LOOCV* leave-one-out cross-validation, *LR* logistic regression, *ML* machine learning, *MLP* multilayer perceptron, *NB* Naive Bayes, *KNN* K-nearest neighbors, *PET* positron emission tomography, *QDA* quadratic discriminant analysis, *RBF* radial basis function, *RF* random forest, *SGD* stochastic gradient descendent, *SVM* support vector machine, *TP53* tumor suppressor protein 53

#### Clinical characteristics of the studies

The 124 studies evaluated in the qualitative synthesis included a total of 45,747 patients with NSCLC. Although the majority of the studies included more than 200 patients, 51 publications had sample sizes below this threshold, with 13 of them including fewer than 100 patients. All studies were retrospective and mostly unicentric (*n* = 92). The number of participant centers was not specified in one study [17]. In terms of geographic origin, most patient datasets were derived from Chinese cohorts (*n* = 87), followed by those from the United States (*n* = 9), and fewer from other countries such as Japan, Canada, and several European nations (Supplementary Table S3). In general, basic clinical and demographic information collected included sex, age, smoking status, TNM stage, histology, and treatment status at the moment of image acquisition, although this information was not available in 15, 12, 23, 49, 33 and 29 studies out of the 124 assessed, respectively. The clinical characteristics of the patients included in the 124 studies are depicted in Table 2. The median [range]/mean ± standard deviation (SD) age of patients was 61.9 [58.93–64.22] years and 61.78 ± 3.60 years, respectively. In terms of sex, the total population was balanced, with 19,305 females and 20,702 males. The smoking history was available for 32,333 patients, and most of them were non-smokers (*n* = 17,293); while smoking history was unknown for 1175 patients. Out of the 75 studies detailing information about the TNM stage, the majority of them (*n* = 49) included information about the four stages (I–IV). Among the 26 studies that did not include patients of all stages, three studies included only early stage patients (stages I and II) [18–20], two included patients with stage II–IV [21, 22], nine included only patients with stages III and IV [23–31] (three of them with a majority of stage IV patients [23–25, 27, 31]), three included only stage IV patients [32–34], one included patients with advanced stage without specifying whether stage III or IV [35], and five included patients with stages I–III without including the most advanced stage [17, 36–39]. A total of 90 studies included patients with adenocarcinoma: 68 exclusively including this histological subtype and 27 including other NSCLC histologies as well. Finally, in most cases (*n* = 85), images were acquired before patients received any treatment, with two studies also including post-treatment images [40, 41]. Only two studies [29, 42] included patients who had received treatment with tyrosine kinase inhibitors (TKIs).

**Table 2:** Clinical characteristics of the studies (*N* = 124) included in the systematic review

Author [ref]	Target oncogene mutation	Design	Total of patients	Sex		Age Mean/median	Histology	Smoking status		TNM stage	Treatment
				Female	Male			Current/former smoker	Non-smoker		
Agüloğlu et al [51]	EGFR ALK	Unicentric	189	59	130	62/–	NSCLC	130	59	Stages I–IV	Naïve
Aerts et al [40]	EGFR	Unicentric	47	–	–	–/–	NSCLC	–	–	–	Naïve + post-treatment images
Agazzi et al [123]	EGFR ALK	Unicentric	84	39	45	–/63	ADC	57	27	–	Naïve
Aide et al [36]	EGFR	Unicentric	109	34	75	–/66	ADC	96	13	Stages II–IV	Naïve
Chang et al [82]	EGFR	Unicentric	583	305	278	–/62	ADC	229	354	Stages I–III	Naïve
Chang et al [104]	ALK	Unicentric	526	272	254	–/58.25	ADC	202	324	Stages I–IV	Naïve
Chen et al [124]	EGFR	Unicentric	223	109	114	64.63/–	NSCLC	55	168	Stages I–IV	Naïve
Chen et al [125]	EGFR	Unicentric	233	105	128	57.5/–	ADC	65	168	Stages I–IV	Naïve
Chen et al [126]	EGFR KRAS*	Multicentric	1045	533	512	Freq./Freq.^¶ †^	NSCLC	564	481	–	Naïve
Cheng et al [32]	EGFR	Unicentric	203	104	99	58.92/–	NSCLC	95	108	Stage IV	Naïve
Choe et al [127]	ALK	Unicentric	503	273	230	62.5/–	ADC	200	303	Stages I–IV	Not specified
Dang et al [17]	EGFR	Not specified	118	55	63	63.82/–	ADC, SCC	–	–	Stages I–III	No treatment*^‡^
Digumarthy et al [128]	EGFR	Unicentric	93	50	43	60/–	ADC, SCC	61	32	–	Naïve
Dong et al [37]	EGFR	Multicentric	132	64	68	58.8/–	NSCLC	42	90	Stages I–III	Naïve
Dong et al [88]	EGFR KRAS	Multicentric	525	250	275	–/65.5	NSCLC	373	152	–	Not specified
Feng et al [89]	EGFR	Multicentric	168	–	–	–/–	NSCLC	–	–	–	Not specified^†§^
Gao et al [90]	EGFR	Unicentric	515	264	251	64/–	ADC	175	–	Stages I–IV	Naïve
Gong et al [91]	EGFR	Unicentric	949	496	436	61.96/–	ADC, NSCLC NOS, SCC	–	–	–	Not specified^†^
Hao et al [129]	ALK	Unicentric	193	102	91	54.26/–	NSCLC	49	144	Stages II and IV	Naïve
He et al [130]	EGFR	Multicentric	758	317	441	55.6/–	NSCLC	358	400	Stages I–IV	Naïve
Hinzpeter et al [92]	EGFR KRAS	Unicentric	128	62	66	62.4/–	ADC, SCC	92	–	Stages I–IV	Naïve
Hong et al [24]	EGFR	Unicentric	201	94	107	58.12/–	ADC	64	137	Stages I–IV	Naïve
Hu et al [33]	EGFR	Unicentric	359	186	173	59.02/–	NSCLC	204	155	Stage IV	Naïve
Huang et al [41]	EGFR	Unicentric	46	–	–	–/–	NSCLC	–	–	–	Naïve + post-treatment images
Huang et al [131]	EGFR	Unicentric	195	72	123	61.14 –	NSCLC	127	68	–	No treatment*^‡^
Huang et al [120]	EGFR	Unicentric	1074	–	–	–/–	NSCLC	–	–	–	Not specified
Huo et al [93]	EGFR	Unicentric	608	272	336	61.7/–	ADC	0	335	Stages II and IV	Naïve
Hou et al [132]	EGFR	Unicentric	206	120	86	–/59	ADC, SCC, ASC^‡^	57	–	Stages I–IV	Naïve
Jia et al [94]	EGFR	Unicentric	503	249	254	–/60.5	ADC	80	423	Stages I–IV	Not specified^†§^
Jiang et al [133]	EGFR	Unicentric	80	32	48	64/62.5	NSCLC	21	59	–	Naïve
Jiang et al [95]	EGFR	Unicentric	692	–	–	59/–	ADC	–	–	–	Naïve
Kawazoe et al [134]	EGFR	Unicentric	164	75	89	70.24/–	ADC	102	62	Stages I–IV	No treatment^§**^
Kawazoe et al [135]	EGFR	Unicentric	172	77	95	70.76/–	ADC	107	65	Stages I–IV	Naïve
Kim et al [136]	EGFR	Multicentric	1280	380	900	70.58/–	NSCLC	556	–	Stages I–IV^††^	Naïve
Kohan et al [96]	EGFR KRAS	Unicentric	157	77	83	62.9/63.6	NSCLC	124	33	–	Naïve
Koyasu et al [137]	EGFR	Unicentric	138	54	84	67.8/–	ADC, SCC	–	–	–	Not specified
Le et al [52]	EGFR KRAS	Multicentric	161	50	111	68.05/–	ADC, NSCLC NOS, SCC	61	100	–	Naïve
Li et al [47]	EGFR	Unicentric	1010	457	553	–/63	ADC	262	748	Stages I–IV	Naïve
Li et al [38]	EGFR	Unicentric	51	19	32	58.1/–	ADC	24	27	Stages I–III	Not specified^†§^
Li et al [83]	EGFR	Unicentric	115	62	53	–/63	NSCLC	36	79	Stages II and IV	Naïve
Li et al 2019 [138]	EGFR	Unicentric	312	164	148	Freq./Freq.^¶†^	ADC, SCC	109	203	Stages II and IV	Naïve
Li et al [98]	EGFR	Multicentric	438	–	–	61.31/–	ADC	–	–	–	Naïve
Li et al [139]	EGFR	Unicentric	75	45	30	62/–	Lung cancer^**§§^	34	41	–	Not specified
Li et al [97]	EGFR	Unicentric	179	103	76	61.51/59.5	ADC	65	114	–	Naïve
Li et al [140]	EGFR	Multicentric	215	75	140	61.64/–	ADC	69	146	–	Not specified
Li et al [141]	EGFR TP53	Unicentric	150	55	95	64.63/–	ADC	82	68	Stages I–IV	Naïve
Liu et al [142]	EGFR	Unicentric	298	172	126	–/60	ADC, Others	136	162	Stages II and IV	Naïve
Liu et al [84]	EGFR	Unicentric	263	121	142	62.5/–	ADC	31	232	–	Not specified^†§^
Liu et al [21]	EGFR	Unicentric	148	63	85	–/61.2	ADC	–	–	Stages II–IV	Naïve
Liu et al [99]	EGFR	Multicentric	346	141	205	66.69/–	ADC, SCC, LCC, PSC	225	121	–	Naïve
Liu et al [143]	EGFR	Unicentric	115	62	53	–/62.75	ADC	36	79	Stages I–IV	Naïve
Liu et al [144]	EGFR	Unicentric	288	165	123	-/58	ADC	233	50	Stages I–IV	Naïve
Lu et al [85]	EGFR	Unicentric	104	64	40	58.27/–	ADC	30	74	Stages I–IV	No treatment*^‡^
Lu et al [145]	EGFR	Multicentric	228^††¶¶^	85^††¶¶^	120^††¶¶^	67.94/–	ADC, SCC, NOS	–	–	Stages 0–IV	Not specified
Lu et al [26]	EGFR	Unicentric	201	99	102	64.81/–	ADC	84	117	Stages III–IV	Naïve
Lu et al [25]	EGFR	Unicentric	274	134	140	65.21/–	ADC ASC	137	137	Stages III–IV	Naïve
Ma et al [105]	ALK	Unicentric	140	87	53	54.19/–	ADC	45	95	Stages II and IV	Naïve
Ma et al [100]	EGFR	Unicentric	175	94	81	57.68/–	ADC	68	107	–	Naïve
Mahajan et al [27]	EGFR	Unicentric	223	76	143	–/55.5	ADC	66^***^	156^***^	Stages III–IV	Naïve
Mei et al [146]	EGFR	Unicentric	296	144	152	58.56/–	ADC	86	210	–	Not specified^†§^
Mu et al [121]	EGFR	Multicentric	681	303	378	61.83/–	ADC, SCC	315	366	Stages I–IV	Naïve
Nair et al [15]	EGFR	Unicentric	50	18	32	–/–	NSCLC	35	15	–	Naïve
Ninomiya et al [44]	EGFR	Multicentric	194	74	120	–/67	NSCLC	128	66	Stages I–IV	Not specified
Ninomiya et al [53]	EGFR	Multicentric	154	86	68	–/67	Lung cancer	73	81	Stages I–IV	Not specified
Njoto et al [43]	EGFR	Unicentric	208	107	101	–/58	ADC	105	103	_	Naïve
Omura et al [18]	EGFR	Unicentric	99	65	34	66/–	ADC	41	–	Stages I–II	Naïve
Ottaiano et al [54]	ALK	Unicentric	57	18	39	Freq./Freq.^¶†^	ADC SCC	52	5	Stages I–IV	Naïve
Rinaldi et al [55]	EGFR KRAS ALK	Unicentric	309	117	192	58.9/-	ADC	228^†††^	71^†††^	Stage IV Others (109 patients)	No treatment*‡
Ríos Velázquez et al [56]	EGFR KRAS	Multicentric	763	459	304	65/–	ADC	548	215	Stages I–IV	Not specified
Rossi et al [101]	EGFR	Multicentric	170	–	–	–/–	ADC	110	30	–	Naïve
Ruan et al [57]	EGFR	Unicentric	100	42	58	–/64.5	NSCLC	33	67	Stages I–IV	Naïve
Shang et al [58]	EGFR	Unicentric	779	434	345	61.9/–	ADC	101^‡‡‡^	650^‡‡‡^	–	No treatment^§§§^
Shao et al [59]	EGFR	Unicentric	1096	–	–	58.26/–	NSCLC	–	–	–	Naïve
Shao et al [60]	EGFR	Unicentric	516	265	251	63.98/–	ADC	175	–	Stages I–IV	Naïve
Shiri et al [16]	EGFR KRAS	Unicentric	150	–	–	69.1 –	ADC, SCC, NOS	–	–	–	Not specified
Shiri et al [61]	EGFR KRAS	Multicentric	136	–	–	–/–	ADC, SCC, NOS	–	–	–	Not specified
Song et al [62]	EGFR	Multicentric	665	336	329	Freq./Freq.^¶†^	ADC	334	331	Stages II and IV	Not specified^‡‡§§§^
Song et al [106]	ALK	Unicentric	335	196	139	57/–	ADC	103	232	Stages I–IV	Naïve
Tan et al [102]	EGFR	Unicentric	155	61	94	66.28/–	ADC	71	84	Stages I–IV	Naïve
Trivizakis et al [63]	EGFR	Unicentric	112	–	–	–/–	ADC, SCC	–	–	–	Not specified
Tu et al [64]	EGFR	Unicentric	404	211	193	59.95/–	NSCLC	114	290	Stages II and IV	Naïve
Wang et al [19]	EGFR	Unicentric	51	35	16	58.45/–	ADC	9	42	Stages 0–II	Not specified
Wang et al [65]	EGFR PD-L1	Unicentric	1262	642	620	57.7/–	ADC, SCC, Others^‡^	452	749	Stages I–IV	Not specified^§§¶¶¶^
Wang et al [48]	EGFR PD-L1	Unicentric	3629	1674	1955	59.29/–	ADC, SCC, Others	1413	1981	Stages I–IV	Naïve
Wang et al [45]	KRAS	Unicentric	258	78	180	62.35/–	NSCLC	166	92	–	No treatment*^‡^
Wang et al [66]	EGFR	Unicentric	269	114	155	63.0/–	ADC	129	–	Stages I–IV	Naïve
Wang et al [86]	EGFR	Unicentric	267	117	150	66/–	ADC	81	–	–	No targeted treatment^****^
Wang et al [147]	EGFR	Multicentric	546	235	311	60.2/–	ADC SCC	209	337	–	Naïve
Wang et al [34]	EGFR	Multicentric	71	32	39	Freq./Freq.^¶†^	ADC	24	47	Stage IV	Naïve
Weng et al [67]	EGFR	Unicentric	301	145	156	64.95/–	NSCLC	110	191	–	Naïve
Wu et al [28]	EGFR	Unicentric	67	29	38	56.35/–	ADC, SCC	34	33	Stages III–IV	Naïve
Wu et al [20]	EGFR	Multicentric	438	252	186	59.65/–	ADC, Other	129	309	Stage I	Naïve
Xiao et al [68]	EGFR	Unicentric	150	59	91	–/58	NSCLC	64	86	–	Not specified
Xiong et al [29]	EGFR	Unicentric	120	67	53	66.18/–	NSCLC	20	100	Stages III–IV	Treated with TKIs
Xu et al [35]	EGFR	Multicentric	485	211	274	Freq./Freq.^¶†^	NSCLC	170	315	Advanced stage	Naïve
Yamazaki et al [69]	EGFR	Unicentric	478	190	288	Freq./Freq.^¶†^	ADC, SCC, Others^‡^	–	–	Stages II and IV	Naïve
Yang et al [23]	EGFR	Unicentric	253	155	98	–/62	ADC	105	148	Stages III–IV	Naïve
Yang et al [70]	EGFR	Unicentric	174	81	93	61.72/–	ADC	59	115	Stages II and IV	Naïve
Yang et al [49]	EGFR	Unicentric	412	223	189	62/–	ADC, SCC	105	307	–	Naïve
Yang et al [22]	EGFR	Unicentric	313	164	149	59.21/–	ADC	105	208	Stages II–IV	Naïve
Yang et al [42]	EGFR	Unicentric	250	–	–	56.35/–	ADC	–	–	–	Treated with TKIs^¶¶††††^
Yao et al [71]	EGFR	Unicentric	202	67	80	–/63	NSCLC	58	89	Stages I–IV	Naïve
Yip et al [72]	KRAS	Unicentric	348	214	134	–/65	ADC, NSCLC NOS, SC. Not available for 1 patient	286	62	Stages I–IV	Naïve
Zhang et al [30]	EGFR	Unicentric	180	46	134	59.7/–	ADC, SCC, Others	119	61	Stages III–IV	Naïve
Zhang et al [87]	EGFR	Unicentric	173	58	115	60.8/–	ADC SCC, LCC, NSCLC-NOS	–	–	Stages I–IV	Naïve
Zhang et al [73]	EGFR	Unicentric	248	113	135	62.23/–	ADC	117	131	Stages I–IV	Naïve
Zhang et al [46]	EGFR	Unicentric	914	493	421	59.79/–	ADC	–	–	–	Naïve
Zhang et al [148]	EGFR KRAS ERBB2 TP53	Unicentric	134	56	78	63.6/–	ADC, SCC, ASC	28	106	–	Not specified
Zhang et al [103]	EGFR	Unicentric	420	201	219	57.43/56.5	ADC	147	273	–	Naïve
Zhang et al [149]	EGFR	Unicentric	115	–	–	–/–	NSCLC	–	–	–	Naïve
Zhang et al [74]	KRAS	Multicentric	119	51	68	Freq./Freq.^¶^	ADC	89	30	–	Naïve
Zhang et al [50]	EGFR	Multicentric	424	198	226	58.50/–	ADC	160	264	–	Naïve
Zhang et al [81]	EGFR	Multicentric	660	315	345	57.64/–	ADC	239	421	Stage I–IV	Naïve
Zhang et al [150]	EGFR KRAS ALK	Multicentric	508	–	–	–	NSCLC	–	–	–	Naïve
Zhang et al [75]	EGFR KRAS ALK	Multicentric	508	177	331	65.9/–	NSCLC	294	214	–	Not specified
Zhao et al [122]	EGFR	Unicentric	616	–	–	–	ADC	–	–	Stages 0–IV	Not specified
Zhao et al [78]	EGFR	Unicentric	637	368	269	59.9/–	ADC	49	588	–	Naïve
Zhao et al [76]	EGFR	Unicentric	88	39	49	64.23/–	ADC	31	57	Stages II and IV	Naïve
Zhao et al [77]	EGFR	Multicentric	640	–	–	–	ADC	–	–	–	Not specified
Zhu et al [39]	EGFR	Unicentric	1092	648	442	59.59/–	ADC	–	–	Stages I–III	Naïve
Zhu et al [31]	EGFRTP53	Unicentric	199	86	113	Freq./Freq.^¶†^	ADC	94	105	Stages III–IV	Naïve
Zuo et al [80]	EGFR	Multicentric	767	372	395	–/62.04	ADC	–	–	Stages I–IV Others (34 patients)	Not specified
Zuo et al [79]	EGFR	Multicentric	383	217	166	–/64.1	ADC	–	–	Stages I–IV	Not specified
Zuo et al [151]	EGFR	Multicentric	478	239	239	61/–	ADC	–	–	Stages I–IV	Naïve

For those studies with the same name for the first author and published the same year, a hashtag was added to unequivocally indicate those that were included in the different meta-analyses and consequently, that are represented in the forest plots. Please note that calculations for sex, age, and smoking status account for all study subjects, including those from any external validation cohort(s), if applicable

^*^The prediction models for KRAS were created and evaluated based on EGFR−. However, some patients with EGFR− did not undergo KRAS detection, and therefore these patients were only used for the analysis of predicting EGFR mutations but not KRAS

^†^These studies provide age data as frequencies establishing an age threshold

^‡^Patients were excluded if treated with RT and/or chemotherapy, but targeted therapy is not specified

^§^CT scans acquired prior surgery; no information on prior treatments

^¶^Sex information not available for 17 patients of the external cohort

^**^Patients did receive target treatment, but no information on the administration of other treatments (immunotherapy and/or chemotherapy) is specified

^††^One extra patient exceeding the total sample size was included in the group of patients with stage IV disease

^‡‡^Table 1 contains an error; a total sample size of 160 is indicated contrary to what appears in the text

^§§^Inferred that NSCLC patients were included as it is specified that 17 patients had 19Del and 20 cases had L858R mutation; EGFR mutations are very rare in SCLC

^¶¶^In this study, there are 23 patients with no information about sex

^***^Smoking status information incomplete (one missing patient)

^†††^Patients with information about smoking status do not account for the total sample size of the study, but no missing values are indicated

^‡‡‡^28 missing values reported

^§§§^Image acquired 3 months before mutation testing; no information about treatments

^¶¶¶^CT images acquired within 1 month before pathological diagnosis

^****^Patients were excluded if treated with targeted therapy, but other treatments are not specified

^††††^Imaging-proven progression on first- or second-generation TKIs; patients underwent chest contrast-enhanced CT at the time of confirmed progression, and the interval between CT and confirmed progression was within 3 days

^‡‡‡‡^In this study, due to the difficulty in obtaining clinical data, only 147 of the 202 patients were included in the statistical analysis of the clinical information to explore the potential association between clinical data and EGFR prediction

*ADC* adenocarcinoma, *ALK* anaplastic lymphoma kinase, *ASC* adenosquamous carcinoma, *CT* computed tomography, *EGFR* epidermal growth factor receptor, *ERBB2* v-erb-b2 avian erythroblastic leukemia viral oncogene homolog 2, *Freq.* frequency, *KRAS* Kirsten rat sarcoma viral oncogene homologue, *LCC* large cell lung carcinoma, *NOS* not otherwise specified, *NSCLC* non-small cell lung cancer, *PCR* polymerase chain reaction, *PD-L1* programmed death ligand 1, *PSC* pulmonary sarcomatoid carcinoma, *RT* radiotherapy, *SCC* Squamous cell carcinoma, *SCLC* small-cell lung cancer, *TKI* tyrosine kinase inhibitor, *TP53* tumor suppressor protein 53

#### Risk of bias in studies

**Fig. 2:**
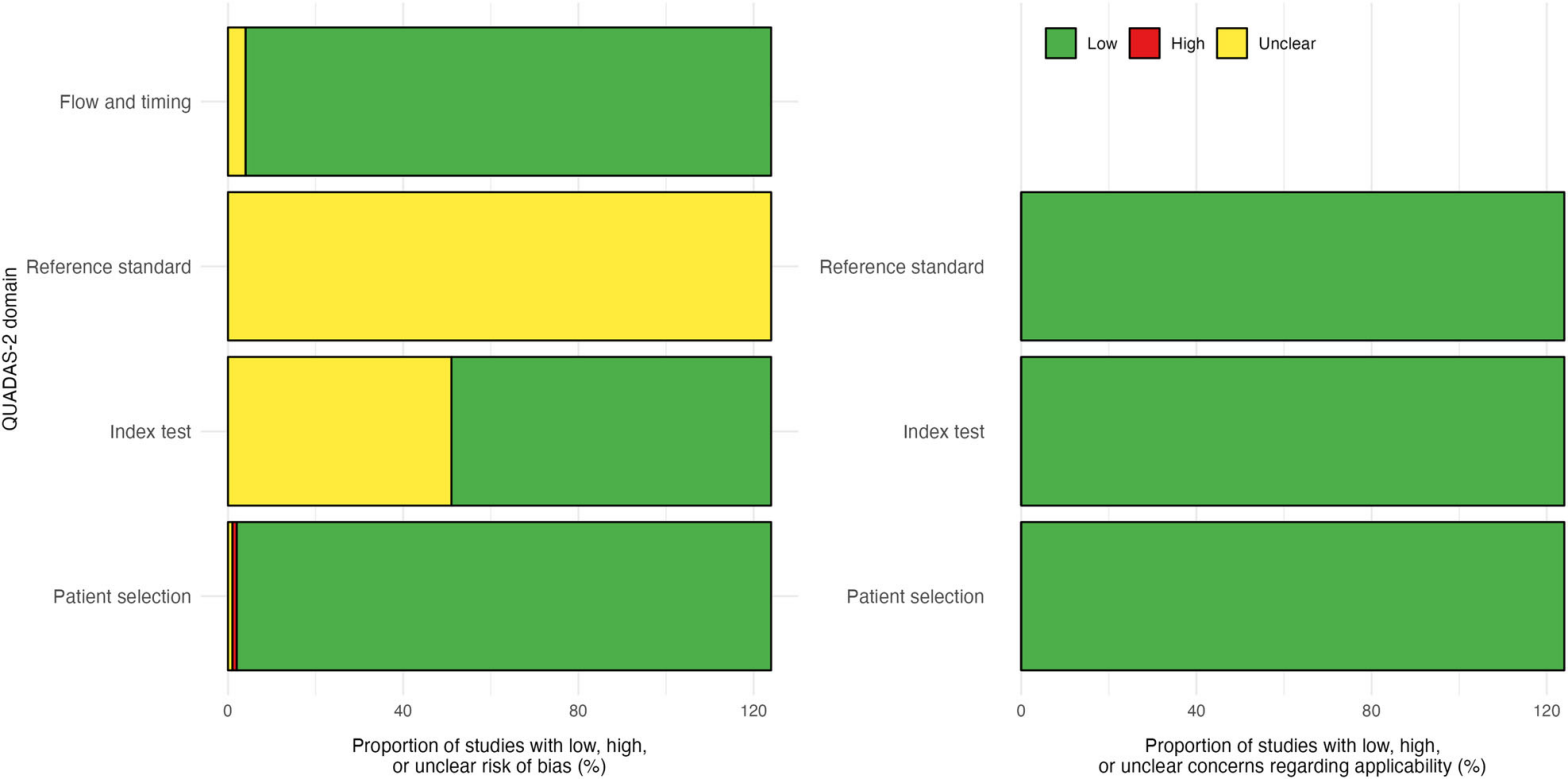
Risk of bias graph summarizing the authors’ judgments for each domain of the Quality Assessment of Diagnostic Accuracy Studies-2 (QUADAS-2) tool, presented as percentages across all included studies (*N* = 124)

Figure 2 summarizes the risk of bias across the included studies. The individual responses for each signaling question and applicability judgment per study are detailed in Supplementary Table S4. Regarding patient selection, most studies (*n* = 122) were judged to have a low risk of bias, while only one study was rated as high [43] and one as unclear [44]. In the domain of the index test, 73 studies were considered to have a low risk, whereas 51 [16, 18–20, 22, 23, 29, 30, 35, 36, 39–41, 43–80] were rated as unclear due to insufficient details on how the test was conducted or interpreted. As for the reference standard, all 124 studies were categorized as unclear. This classification was applied because none of the studies explicitly stated whether genetic testing was conducted blind to the results of the imaging-based model. However, since molecular testing is inherently independent from image-based predictions and does not rely on subjective interpretation, the practical risk of bias in this domain is likely to be minimal. Concerning flow and timing, 120 studies were rated as low risk and four [18, 43, 44, 53] as unclear, mostly due to incomplete reporting of the interval between index test and reference standard or potential inconsistencies in patient inclusion and follow-up.

### Quantitative analysis (meta-analysis)

A total of 51 studies, encompassing 21,766 patients, met the inclusion criteria for quantitative assessment. Three distinct meta-analyses were conducted for radiomics-based models: (1) EGFR mutation (*n* = 47 studies) [15, 20, 23, 26, 28, 30, 32, 33, 37, 39, 44, 46–50, 52, 53, 56, 60, 64, 66, 67, 76, 81–103]; (2) ALK translocation (*n* = 3 studies) [104–106]; (3) KRAS mutation (*n* = 6 studies) [45, 52, 56, 88, 92, 96]. Five studies developed models for both EGFR and KRAS mutations [52, 56, 88, 92, 96]. Additionally, a separate meta-analysis was performed for combined models (radiomics features + clinical variables) predicting EGFR mutation. Supplementary Table S5 summarizes studies included in all conducted meta-analyses. Details on the radiomics features for EGFR, ALK, and KRAS models are outlined in Supplementary Table S6, Supplementary Table S7, and Supplementary Table S8, respectively.

#### EGFR

**Fig. 3:**
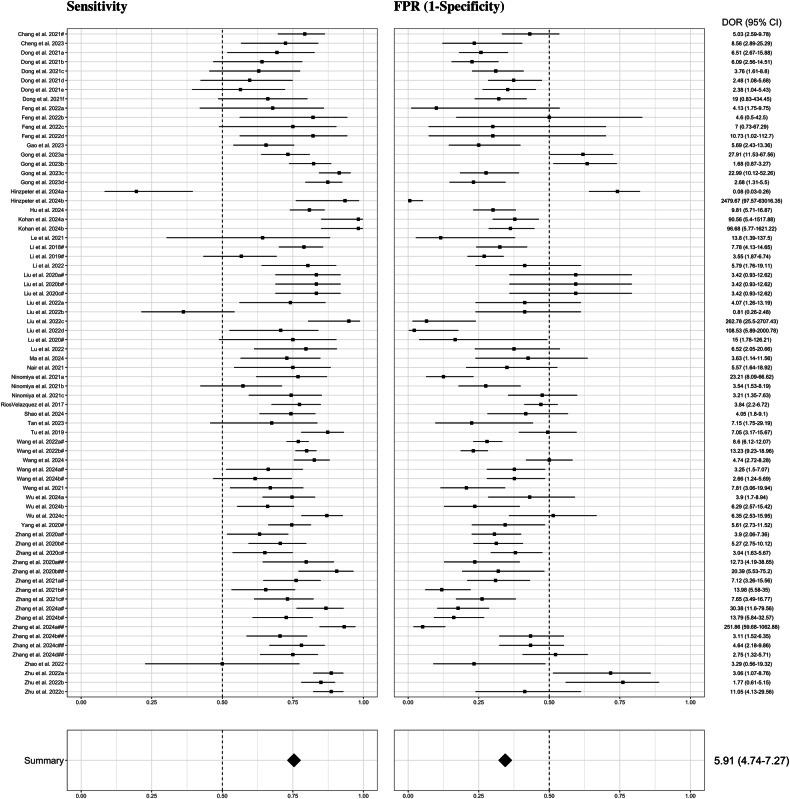
Forest plots of the included studies developing radiomics models using machine learning and/or deep learning methods for the prediction of EGFR mutation status. Numbers are estimated with 95% CIs in brackets and indicated by horizontal lines. For those studies with the same name for the first author and published in the same year, a hashtag was added to unequivocally tag them as done in Tables 1 and 2 and in the reference list. EGFR, epidermal growth factor receptor; CI, confidence interval; DOR, diagnostic odds ratio; FPR, false positive rate

Results of the meta-analysis focused on models built with radiomics features are summarized in Fig. 3. A hierarchical sROC curve was plotted for the included 37 studies (72 different models) [15, 20, 23, 26, 32, 33, 39, 44, 46–48, 50, 52, 56, 60, 64, 66, 67, 76, 81–92, 96, 97, 99, 100, 102, 103] that evaluate the performance of AI algorithms in predicting EGFR mutation status in NSCLC (Supplementary Fig. S1). As observed, radiomics-based models exhibited high diagnostic performance in predicting EGFR mutation status with an overall AUC of 0.769. The AI algorithms’ sensitivity in determining the EGFR mutation status varied from 0.196 to 0.982, resulting in an estimate of 0.754 (95% CI 0.727–0.780). The FPR of these algorithms ranged from 0.006 to 0.761, with an estimate of 0.344 (95% CI 0.308–0.381). This corresponds to a pooled specificity of 0.656 (95% CI 0.619–0.692). Detecting a positive case for EGFR mutation was almost six times more likely than not detecting it (DOR = 5.91 [95% CI 4.74–7.27]).

**Fig. 4:**
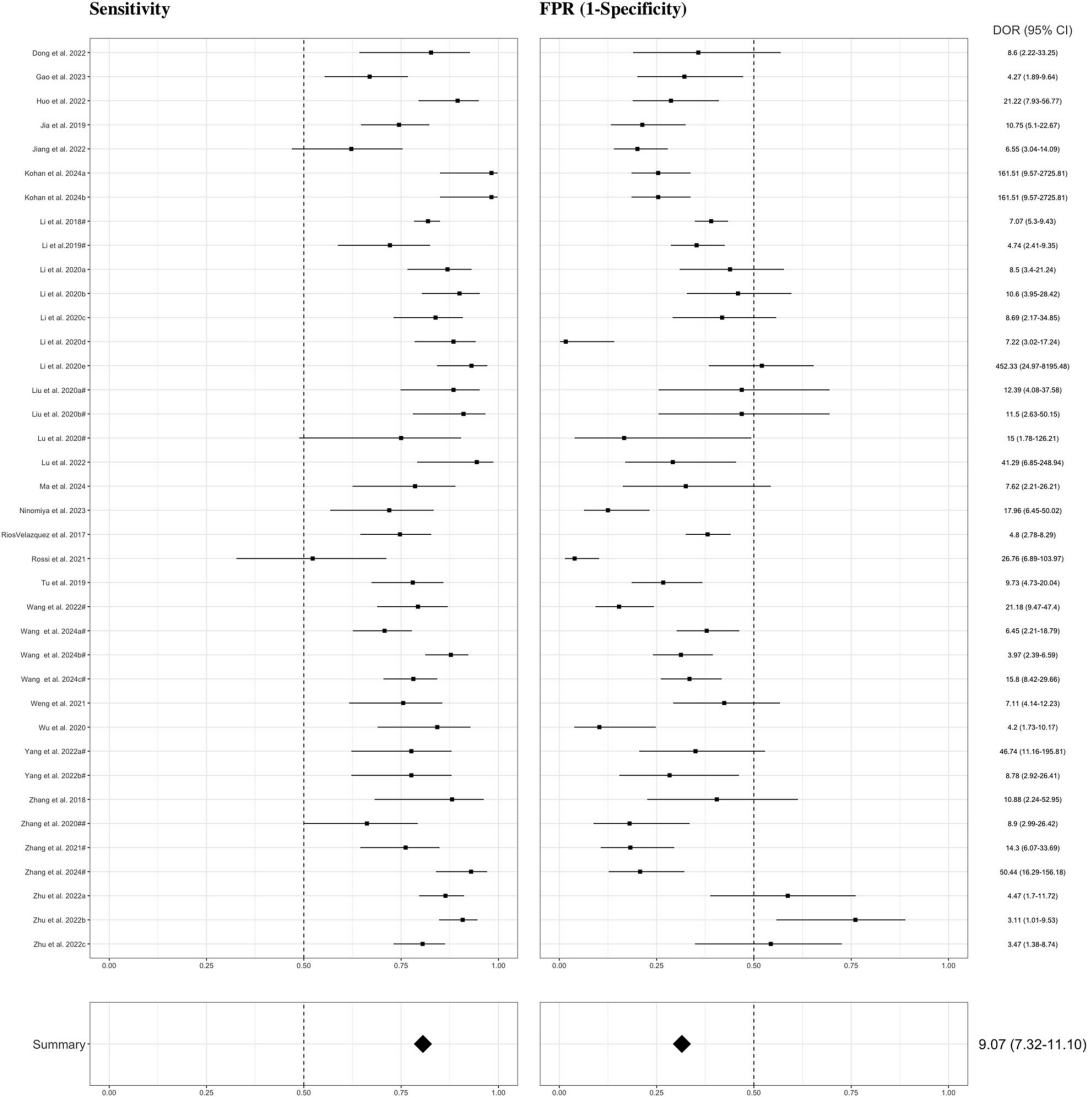
Forest plots of the included studies developing combined models (radiomics + clinical data) using machine learning and/or deep learning methods for the prediction of EGFR mutation status. Numbers are estimates with 95% CIs in brackets and indicated by horizontal lines. For those studies with the same name for the first author and published in the same year, a hashtag was added to unequivocally tag them as done in Tables 1 and 2 and in the reference list. EGFR, epidermal growth factor receptor; CI, confidence interval; DOR, diagnostic odds ratio; FPR, false positive rate

The effect of adding clinical variables to radiomics models or to those including both radiomics and deep features [48] (models including clinical data and radiomic or deep features referred to in this work as combined models) in the prediction of EGFR mutation was also analyzed. This meta-analysis included 29 studies (38 models) [26, 28, 30, 37, 39, 46–50, 53, 56, 64, 67, 81, 83–86, 90, 91, 93–96, 98, 100, 101, 103]. Please note that from [86], all combined models provided were included in the analysis (radiomic features plus clinical data, deep learning plus clinical data, deep learning plus radiomic features plus clinical data). Results are depicted in Fig. 4 and sROC curve in Supplementary Fig. S1. Overall, the performance of combined models slightly improved compared to radiomics models, with an AUC of 0.821 and a sensitivity of 0.806 (95% CI 0.776–0.833): model’s sensitivity ranging from 0.523 to 0.982. The FPR resulted similar with a value of 0.315 (95% CI 0.270–0.364; model’s FPR ranging from 0.017 to 0.761). The corresponding pooled specificity was 0.685 (95% CI 0.636–0.730). Detecting a positive case for EGFR mutation with combined models was more than nine times more likely than not detecting it (DOR = 9.07 [95% CI 7.32–11.10]). Additionally, we conducted an extended analysis incorporating supplementary models developed by certain authors [70, 76, 79]. These models, while excluding clinical data, integrated radiomic and deep features. The performance metrics exhibited minimal variation (AUC = 0.825, sensitivity = 0.805 [95% CI 0.777–0.831], FPR = 0.306 [95% CI 0.263–0.353]), as these models contributed only marginally to the combined pool of studies integrating radiomic and clinical information (Supplementary Figs. S2, S3).

#### ALK

**Fig. 5:**
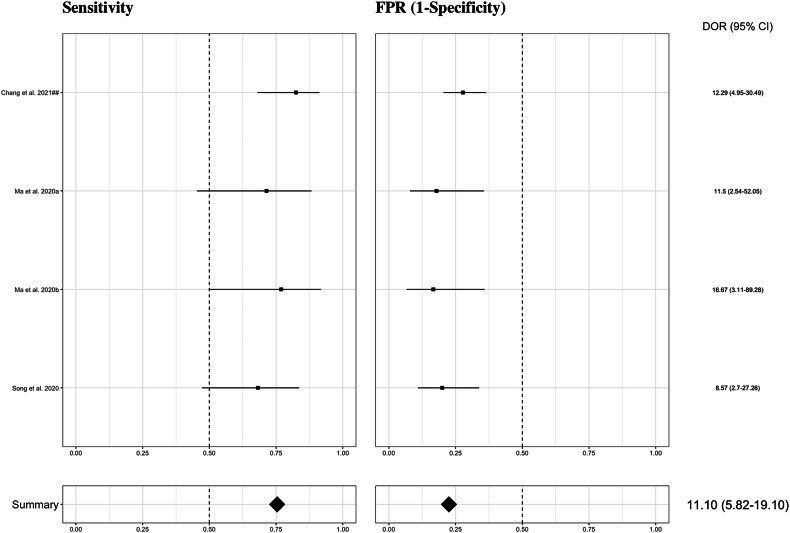
Forest plots of the included studies developing radiomics models using machine learning and/or deep learning methods for the prediction of ALK mutation status. Numbers are estimates with 95% CIs in brackets and indicated by horizontal lines. For those studies with the same name for the first author and published the same year, a hashtag was added to unequivocally tag them as done in Tables 1 and 2 and in the reference list. ALK, anaplastic lymphoma kinase; CI, confidence interval; DOR, diagnostic odds ratio; FPR, false positive rate

The meta-analysis focused on radiomics-based models included three studies (four models) [104–106]. An overall AUC of 0.831 was obtained for the prediction of ALK translocations, with a sensitivity ranging from 0.682 to 0.825, resulting in an estimate of 0.754 (95% CI 0.638–0.841). The FPR of these algorithms ranged from 0.167 to 0.277, with an estimate of 0.225 (95% CI 0.163–0.302). This yields a specificity of 0.775 (95% CI 0.698–0.837). Detecting a positive case for ALK aberration was 11 times more likely than not detecting it (DOR = 11.10 [95% CI 5.83–19.10]) (Fig. 5 and Supplementary Fig. S4).

#### KRAS

**Fig. 6:**
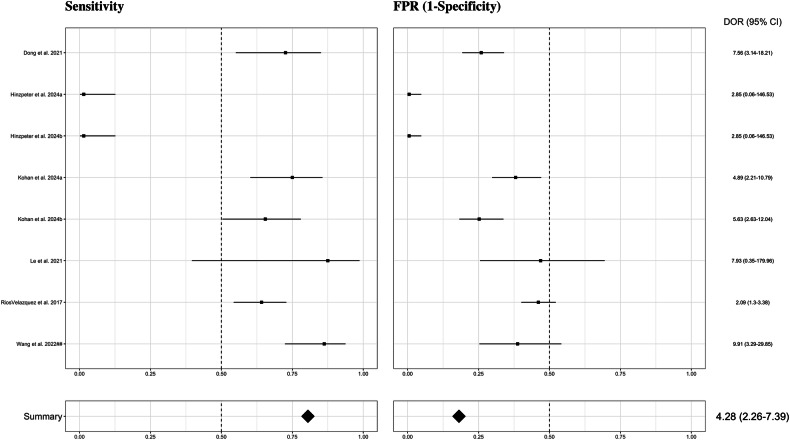
Forest plots of the included studies developing radiomics models using machine learning methods for the prediction of KRAS mutation status. Numbers are estimates with 95% CIs in brackets and indicated by horizontal lines. For those studies with the same name for the first author and published the same year, a hashtag was added to unequivocally tag them as done in Tables 1 and 2 and in the reference list. CI, confidence interval; DOR, diagnostic odds ratio; FPR, false positive rate; KRAS, Kirsten rat sarcoma viral oncogene homolog

Six studies (eight models) evaluating radiomics-based models for KRAS mutation prediction were included [45, 52, 56, 88, 92, 96]. Results are shown in Fig. 6 and Supplementary Fig. S5. KRAS mutation was predicted with an overall AUC of 0.735 and a sensitivity of 0.475 (95% CI 0.153–0.820); model’s sensitivity ranging from 0.015 to 0.875. The FPR was 0.181 (95% CI 0.054–0.461); model’s FPR ranging from 0.005 to 0.468. The pooled specificity for KRAS mutation was 0.819 (95% CI 0.539–0.946). Detecting a positive case for KRAS mutation with radiomics-based models was more than four times more likely than not detecting it (DOR = 4.28 [95% CI 2.26–7.4]). As observed, performance varied substantially across studies, reflecting considerable heterogeneity.

#### Assessment of small-study effects

Finally, to assess potential small-study effects and publication bias, we performed Deeks’ asymmetry test, the recommended method for diagnostic test meta-analyses. Only the combined EGFR models yielded a statistically significant result (*p* < 0.05), suggesting potential asymmetry. However, visual inspection and sensitivity analysis indicated this was driven by a single influential study with disproportionate effect size and precision. No significant asymmetry was observed for the EGFR radiomics-only models, nor for KRAS or ALK. The corresponding funnel-like plots and *p*-values are presented in Supplementary Figs. S6–S9.

### Meta-regression and subgroup analysis

The possible effects of different predictors on the predictive performance of the models were evaluated for EGFR mutation (not enough studies were available for ALK or KRAS mutations). A total of 47 studies were included in this analysis [15, 20, 23, 26, 28, 30, 32, 33, 37, 39, 44, 46–50, 52, 53, 56, 60, 64, 66, 67, 76, 81, 82, 84–102, 107, 108]. Subgroups comprised 25 radiomics-only models [15, 23, 32, 33, 39, 44, 46, 48, 52, 76, 81, 82, 85, 88–92, 97, 99, 107] and 22 combined models [26, 28, 30, 37, 47, 49, 50, 53, 56, 64, 67, 84, 86, 87, 93–96, 98, 100, 101, 108]; 38 studies using ML [15, 23, 26, 28, 30, 32, 33, 37, 39, 44, 49, 50, 52, 53, 56, 60, 64, 66, 67, 76, 82, 84, 85, 89, 90, 92–102, 107, 108] and 9 using DL [20, 46–48, 81, 86–88, 91]; 32 based on non-contrast-enhanced CT [26, 30, 32, 37, 39, 46–48, 52, 60, 64, 66, 67, 76, 81, 82, 85–90, 92, 94, 95, 97–99, 101, 102, 107, 108] and 10 on contrast-enhanced CT [15, 23, 28, 33, 44, 53, 84, 93, 96, 100] (two studies using both approaches and three in which this information was unknown were excluded) and 33 studies with manual segmentation [15, 20, 23, 26, 28, 30, 32, 33, 37, 39, 44, 46–50, 52, 53, 56, 60, 64, 66, 67, 76, 81, 82, 84–102, 107, 108] and 11 with semi-automatic approaches [23, 46, 56, 60, 67, 76, 84, 90, 92, 94, 96] (three studies in which this information was unknown were excluded). Neither age, nor the use of contrast, nor the type of segmentation, nor the model, nor the AI methodology, yielded statistically significant results (Supplementary Table S9).

## Discussion

At present, invasive molecular testing is the gold standard for advanced NSCLC diagnosis and genotyping [109, 110]. However, limitations including inadequate samples—few cells or hemorrhagic samples—[111, 112], the risk of invasive procedures in fragile cancer patients, and lengthy turnaround times [109], warrant validation and integration of new procedures in clinical practice. In recent years, radiomics has shown encouraging results in prognosis and prediction in lung cancer [113]. This straightforward, reproducible methodology enables clinicians to gather important data about the type of tumor, its aggressiveness, progression, and response to therapy [114]. Besides, unlike other methodologies, relying solely on medical images like CT scans acquired as part of the standard patient journey, radiomics captures patient- and tissue-level heterogeneity, representing an affordable tool both in terms of resource consumption and costs. Proper validation, however, is crucial for standardization before incorporating such methodology into routine clinical workflows.

This systematic review and meta-analysis assess the performance of various imaging-based models for predicting three common oncogene mutations—EGFR, ALK and KRAS—in NSCLC with a special focus on AI methodologies. Although this has been previously addressed by Nguyen et al [115], and more recently by Chen et al [116], both reviews focused primarily on technical performance and did not explore clinical applicability in depth (a detailed comparison of the methodological scope and contributions of these three reviews is provided in Supplementary Table S10). Our study offers a distinct perspective by stratifying approaches according to input type (clinical-only, radiomics-only, or combined), which allows for direct comparison of their relative value from a clinical decision-making standpoint. Additionally, we limited inclusion to methods based on CT scans, the standard imaging modality in NSCLC clinical workflows, as previously discussed, thereby ensuring methodological homogeneity and enhancing translational relevance. Moreover, in contrast to the common practice in the literature—and specifically to the approach adopted by Nguyen et al [115] and Chen et al [116], who extracted only the best-performing models per study for quantitative synthesis—we included all models that met pre-defined criteria, regardless of their performance. By including all comparable models, this analysis provides a broader view of the evidence, highlighting the heterogeneity and the importance of larger, multicentric study designs to achieve more robust conclusions. To synthesize this heterogeneity in a statistically sound manner, we applied a bivariate random-effects model (Reitsma et al [14]) and conducted subgroup meta-regression analyses, allowing us to explore the influence of methodological and clinical covariates on model performance. Thus, our meta-analyses reveal that AI-based models using CT-derived radiomics features perform well in predicting EGFR and ALK mutations, with sensitivities of 0.754 [95% CI (0.727–0.780)] and 0.754 [95% CI (0.638–0.841)], respectively. However, the prediction performance for KRAS was suboptimal, with limited sensitivity (0.475 [95% CI (0.153–0.820)]), primarily attributable to the heterogeneity introduced by the models developed by Hinzpeter et al [92]. Although heterogeneity could not be reliably assessed using the method developed by Zhou and Dendukuri [117], the negligible sensitivity observed in the two models (0 for both) developed by Hinzpeter et al [92] negatively impacted the overall sensitivity for KRAS prediction in the analysis. Given the considerable variability in model performance (e.g., sensitivity ranging from 0.015 to 0.875), these findings should be interpreted as exploratory and hypothesis-generating. Standardization in modeling strategies, feature selection, and validation protocols will be essential in future studies to reduce heterogeneity and allow for more conclusive assessments of KRAS prediction. Of note, the sensitivity and FPR values reported reflect the specific operating thresholds used in each individual study. In contrast, the SROC curve provides a global summary of diagnostic performance across all possible thresholds. These two perspectives are complementary: while the SROC reflects the overall discriminative ability of the models, study-specific metrics offer a more direct insight into real-world performance and misclassification risks under routine clinical conditions. Finally, although our results do not decisively indicate whether the inclusion of clinical variables enhances model performance, we did observe a modest trend toward improved predictive accuracy in EGFR models when clinical features were added (AUC increase from 0.769 to 0.821), consistent with previous reports [116]. While this 5% absolute gain in AUC may appear limited, we believe it can have meaningful implications—particularly in large-scale screening or triage scenarios, where even small improvements in discriminative ability can translate into substantial differences in patient outcomes and healthcare resource allocation. Clinical variables may help reduce false positives and enhance specificity, which is especially valuable when these models are used to prioritize molecular testing. This observation is consistent with multiple radiomics and deep learning studies incorporated in our work, which improved specificity when clinical data are integrated—typically with absolute gains ranging from 0.05 to 0.24 depending on the modeling strategy and feature selection approach [39, 56, 64, 84, 86, 88, 96, 100]. Nonetheless, the added complexity in data collection and potential challenges in model interpretability must be carefully weighed, and future prospective validations are needed to better assess this trade-off.

Our findings support the use of radiomics as a potential screening tool for determining oncogene mutation status, with a specific focus on CT-based models to align with the standard clinical workflow, given CT’s widespread use in advanced NSCLC (PET/CT is commonly used for localized and advanced stages) [118]. Conclusively, future studies should prioritize additional validation and consider essential aspects, starting with ensuring a minimum sample size to guarantee the reliability of results obtained with AI-based models [119, 120]. In both our systematic review and meta-analyses, more than half of the studies were conducted in > 200 patients (*n* = 72/124 and *n* = 32/51 (*n* = 29/47 for EGFR, *n* = 2/3 for ALK and *n* = 3/6 for KRAS)), but still a sizable number had small sample sizes, which limited the relevance of their conclusions. Multicentric designs would also be desirable to get more solid conclusions, an approach that few studies followed (*n* = 31/124 in the systematic review and *n* = 15/51 in the meta-analysis (*n* = 10/47 for EGFR, *n* = 0/3 for ALK and *n* = 3/6 for KRAS)). Second, including independent cohorts for external validations would reinforce the results, leading to more robust and reproducible models. Out of the 124 studies included in the qualitative analysis, (*n* = 21) used external cohorts for validation, of which seven were included in the EGFR meta-analysis [33, 44, 46, 49, 85, 91, 101]. Finally, it is important that patient cohorts reflect real-world clinical scenarios. Thus, for optimal diagnostic applicability, studies should focus on treatment-naïve populations to avoid potential therapy-related confounding effects, a criterion observed in most evaluated studies, but lacking in some. Preferably, research should target stage III–IV NSCLC patients, especially stage IV, aligning with clinical guidelines recommending molecular testing as a priority for these patients [109, 110]. As demonstrated in this work, most of the studies published so far do not provide information on TNM stage or include patients from all stages.

Despite the heterogeneity of the studies evaluated, this work demonstrates radiomics’ potential in determining oncogene mutation status. Thus, AI-based models using radiomics from CT scans could serve as effective non-invasive screening tools for detecting targetable driver mutations in NSCLC, offering good sensitivity and moderate specificity. Nonetheless, we acknowledge that any clinical implementation of such tools must be preceded by robust prospective validation. These tools are not intended to replace gold standard techniques like PCR or next-generation sequencing–routinely performed before any treatment decision–but rather facilitate the timely identification of ideal candidates who would benefit most from genetic testing, thereby saving time while reducing costs and preserving valuable samples obtained invasively. Thus, AI-based predictions should be viewed as adjunctive tools aimed at optimizing pre-test probability, rather than as stand-alone diagnostics. Given their high sensitivity, these models can help ensure that most mutation-positive cases are correctly flagged and subsequently confirmed through laboratory-based testing. Importantly, this review does not aim to advocate for immediate clinical use, but to synthesize current evidence and identify promising directions for future research. In this regard, prospective validation studies and the harmonization of imaging protocols and AI methodologies will be key to ensuring safe and effective clinical translation. As with any screening strategy, however, AI-based tools for mutation prediction carry the risk of false positives, which may lead to unnecessary confirmatory tests or misinterpretations. This issue has been well documented in the context of lung cancer screening with low-dose CT (LDCT), where false-positive results have been associated with overdiagnosis and potentially harmful downstream procedures [121]. Although our work does not focus on cancer screening per se, the same concern is relevant when applying AI models to enrich or triage patients for molecular testing. Several approaches could help mitigate this risk, including multimodal integration of clinical and radiological data to enhance specificity, the use of stricter decision thresholds when models are used in a screening context, hierarchical workflows combining complementary predictive tools, and rigorous external validation and calibration to ensure proper model interpretation. While in our study, the addition of clinical variables resulted in only a marginal improvement in predictive performance, their potential to reduce false positives in real-world settings should be further explored in prospective validations. These strategies, together with efforts to increase model transparency and systematically report false positives in clinical studies, may improve the safety and clinical usefulness of radiomics-based screening tools. Ultimately, while this discussion has emphasized the clinical burden of false positives, it is equally important to acknowledge the impact of false negatives, which may lead to missed opportunities for targeted therapies in patients with actionable mutations. Therefore, sensitivity remains a key priority in the assessment of these tools.

The evaluation of factors influencing EGFR mutation prediction yielded non-significant results, likely due to limited studies and missing data. Nevertheless, certain variables, though not statistically significant, may be crucial in developing accurate models for potential clinical use. Some studies in our qualitative analysis explored methodological aspects’ impact on model performance [41, 61]. For instance, Huang et al [41] found that interobserver variability in tumor segmentation affects radiomics’ ability to predict oncogene mutation, suggesting automatic or semi-automatic models may be preferable. Feature harmonization across centers also emerges as a relevant topic. Shiri et al [61], for example, applied ComBat harmonization to radiomics features extracted from multicenter CT datasets and reported a substantial improvement in model performance for both EGFR and KRAS mutation prediction—up to a 10–15% increase in predictive performance. These findings underscore the importance of harmonization techniques when developing models intended for multicenter use, as scanner- and protocol-related heterogeneity can significantly compromise generalizability. Although only a minority of studies in our review applied harmonization techniques, and these were not systematically reported, their potential to enhance model robustness across clinical settings warrants further attention in future multicenter developments. Other authors highlighted the impact of experimental settings’ variability [72] and CT slice thickness on radiomics-based model predictiveness [38]. The choice of AI methodology is also noteworthy. Although no significant differences in EGFR mutation prediction were found between ML and DL methods, the latter may offer advantages. Thus, while radiomics analysis involves lesion segmentation and feature extraction, a time-consuming and variable process, DL models only require a lesion bounding box, reducing variability. On the other hand, DL models, particularly end-to-end CNN models, like those included in our study [27, 46, 47, 59, 62, 65, 68, 88, 122–124], are generally more complex and capable of solving intricate problems. Considering the available evidence, it seems reasonable to think that methodological approaches should be carefully revised when validation studies are designed and conducted. More broadly, these findings highlight the need for greater methodological transparency and standardization in primary research, including consistent reporting of imaging protocols, segmentation strategies, and AI model characteristics, to enable more informative and robust subgroup analyses in future systematic reviews. In this regard, and to ensure full transparency in the present work, we applied Deeks’ funnel plot asymmetry test for each oncogene. No significant asymmetry was detected for EGFR (radiomics-only), ALK, or KRAS, suggesting low risk of publication bias in the included studies. Nevertheless, it is important to note that Deeks’ test evaluates the association between effect size and study precision; therefore, significant results should be interpreted with caution, especially when based on a limited number of studies. The only statistically significant result emerged in the combined EGFR models, although this was largely attributable to a single influential study, as confirmed by sensitivity analysis. These findings complement our efforts to minimize bias in the meta-analysis and underscore the importance of comprehensive and transparent reporting in future research.

Our study has limitations due to the retrospective/unicentric nature and heterogeneity of included publications, impacting the strength of our conclusions. The scarcity of evidence for ALK and KRAS mutations adds difficulty to drawing firm conclusions in these cases. Another important consideration would be related to the methodological approach of the studies. Thus, most of them are exclusively focused on the tumor, without considering other relevant aspects, such as its distance to the mediastinum, surrounding lung parenchyma, or its location in the lung, to name a few. Moreover, the inconsistent reporting of key methodological parameters—such as contrast enhancement protocols, segmentation strategy, or AI pipeline details—limited our capacity to conduct more granular subgroup or meta-regression analyses to explore the sources of heterogeneity in model performance. We firmly believe that taking these factors into account could also help to improve the performance of radiomics models. However, our work provides the most up-to-date and comprehensive analysis of the available evidence on imaging-based models for predicting key NSCLC oncogene mutations, emphasizing a clinical approach and AI models. Despite limitations, our review and meta-analyses aim to guide future research in the field.

In conclusion, radiomics-based models offer a useful and non-invasive method for determining the status of EGFR mutations in NSCLC and seem to retain similar predictive value at least for ALK mutations. Additionally, although the inclusion of clinical variables tends to increase the performance of the models, further validation is required.

## Supplementary information

ELECTRONIC SUPPLEMENTARY MATERIAL

Supplementary Figure S9

Supplementary material

## Data Availability

All data used to produce this study were gathered from published studies. The key terms and search strategies built to retrieve studies are available in Supplementary Table 1 and in the Supplementary Material. The list of included studies is available in Supplementary Data 1. All other relevant data that support the findings of the study are available from the corresponding author upon reasonable request. This work was available as a preprint at: https://www.medrxiv.org/content/10.1101/2024.05.31.24308261v1.

## Abbreviations

AI: Artificial intelligence
ALK: Anaplastic lymphoma kinase
CLAIM: Checklist for Artificial Intelligence in Medical Imaging
CT: Computed tomography
CTD: Contrast-enhanced diagnostic quality
DL: Deep learning
DOR: Diagnostic odds ratio
EGFR: Epidermal growth factor receptor
FPR: False positive rate
KRAS: Kirsten rat sarcoma virus
ML: Machine learning
NSCLC: Non-small cell lung cancer
PET: Positron emission tomography
PRISMA: Preferred Reporting Items for Systematic Reviews and Meta-Analysis
sROC: Summary receiver operating characteristic
TKIs: Tyrosine kinase inhibitors
