# Supplementary material for: Prediction of oncogene mutation status in non-small cell lung cancer: a systematic review and meta-analysis with a special focus on artificial intelligence-based methods
Source: Eur Radiol. 2025 Sep 8;36(3):2157–85. doi: 10.1007/s00330-025-11962-x (PMC12963223; doi:10.1007/s00330-025-11962-x)
Supplement: Supplementary file 1 — ELECTRONIC SUPPLEMENTARY MATERIAL [file 330_2025_11962_MOESM1_ESM.pdf]

Prediction of oncogene mutation status in non-small cell lung cancer: A systematic review and meta-analysis with a special focus on artificial intelligence-based methods

ELECTRONIC SUPPLEMENTARY MATERIAL

PROSPERO registration

This systematic review and meta-analysis was initially registered in PROSPERO (https://www.crd.york.ac.uk/PROSPERO) on July 29, 2022. Further amendments were performed on October 5, 2022. Amendments included:

- EMBASE as an additional search database
- “Predictive model” was replaced by “classification model”

Inclusion criteria

Papers were included in the qualitative synthesis (systematic review) if meeting the following inclusion criteria based on Patient, Index test, Comparator, Reference test, Diagnosis of reference (PIRD) questions: 1) being focused on the ability of radiomics to predict oncogene mutation status in NSCLC; 2) radiomics features were extracted from CT or from F-18 fluoro-deoxy-glucose (FDG)/CT scans; 3) a full text was available; 4) were written in English, since it is the international language of science, and therefore used in high-impact journals.

Exclusion criteria

Papers describing studies conducted using MRI scans (not the standard of care for NSCLC patients) or performed in phantom or animal models, or published as case reports, editorials, reviews, poster presentations, letters, editorials, or meeting abstracts were excluded. Papers not on the field of interest were also excluded.

For the quantitative synthesis (meta-analysis), the following additional exclusion criteria were applied: 1) oncogene mutation status was not the primary objective of the paper; 2) were focused on specific mutation subtypes; 3) did not apply AI-based methodologies (In this review, any study that applied core data science practices—such as dataset partitioning into training and test sets, cross-validation, or feature selection—was considered to use machine learning methods, regardless of the predictive model employed (e.g., logistic regression). Conversely, studies using logistic regression without

these techniques were classified under traditional statistical models); 4) developed simultaneous multi-output models (i.e., predicting more than one mutation simultaneously) or discriminant models that did not allow the independent calculation of diagnostic performance metrics for each mutation; 5) sensitivity or specificity metrics were not available and could not be calculated; 6) were not comparable with the other articles included (model was developed based on intra- and extra-tumor derived radiomics features); 7) only included models developed with a combination of quantitative features extracted from PET/CT or from PET images (strictly adhering to a clinical perspective, PET scanning equipment is not always available and CT remains the standard of care for NSCLC patients); 8) did not reach a sufficient quality score according to the quality assessment (described below).

Quality assessment

Classification, image reconstruction, text analysis, and workflow optimization are some of the applications of AI in medical imaging that are addressed by CLAIM, which is modeled after the Standards for Reporting of Diagnostic Accuracy Studies (STARD) guideline [1-4]. CLAIM checklist consists of 42 items divided into the conventional sections included in peer-reviewed scientific articles: title or abstract (1 item), abstract (1 item), introduction (2 items), methods (28 items subdivided into study design [2 items], data [7 items], ground truth [5 items], data partitions [3 items], model [3 items], training [3 items] and evaluation [5 items]), results (5 items subdivided into data [2 items] and model performance [3 items]), discussion (2 items) and other information (3 items). The CLAIM guideline offers a roadmap for writers and reviewers with the intention of fostering clear, open, and verifiable scientific discourse on the use of AI in medical imaging [5].

For our quality assessment, a score was calculated for each paper ([total score, 42 - number of “not applicable” fields in each case]). A cut-off value of at least half of the total score after removing the “not applicable” items was established for the inclusion in the quantitative analysis. Therefore, this cut-off value varied for each study depending on the number of items that were applicable from among the 42 total items included in the CLAIM checklist (e.g., a cut-off value of 19 was established for those studies in which only 38 items of the checklist were applicable). **See Supplementary Table 2**. The assessment of the rigor, quality, and generalizability of the work of all enrolled studies was performed by three reviewers.

The Risk of Bias (RoB) as well as applicability concerns of the studies included in our systematic review were assessed using the QUADAS-2 tool[7]. This tool is specifically designed for diagnostic accuracy studies and comprises four domains. Each domain

includes a set of signaling questions to assess the risk of bias, as well as applicability concerns for the first three domains. The signaling questions were answered using the categories “Low”, “High”, or “Unclear” RoB. Applicability concerns were evaluated using “Yes, “No”, or “Unclear”, which correspond to “Low”, “High”, and “Unclear” applicability concerns, respectively.

1. **Patient selection**. Evaluates whether the selection of patients avoided bias and whether the included population is relevant to the review question.
   Signaling questions:
   - **Q1:** Was a consecutive or random sample of patients enrolled?
   - **Q2:** Was a case-control design avoided?
   - Q**3:** Were inappropriate exclusions avoided?

   Applicability:

   - **A1:** Are the patients included representative of those who would receive the test in real-world practice?
2. **Index test.** Assesses potential bias in how the index test (e.g., radiomics-based model) was conducted and interpreted, as well as whether the test is applicable to the clinical context.
   Signaling questions:
   - **Q4:** Were index test results interpreted without knowledge of the reference standard results?
   - **Q5:** Was a prespecified threshold used?

   Applicability:
   - **A2:** Does the index test reflect the technology and procedures relevant to current clinical practice?
3. **Reference standard.** Examines whether the reference standard accurately identifies the target condition and whether its use is appropriate to the context of the review.
   Signaling questions:
   - **Q6:** Is the reference standard likely to correctly classify the target condition?
   - **Q7:** Were reference standard results interpreted without knowledge of the index test?

   Applicability:

- **A3:** Is the reference standard suitable for confirming mutation status in NSCLC?

4. **Flow and timing**. Focuses exclusively on risk of bias. It evaluates the sequence and timing between index test and reference standard, and whether all patients were appropriately included in the analysis.
   Signaling questions:
   - **Q8:** Was the interval between index test and reference standard appropriate?
   - **Q9:** Did all patients receive the same reference standard?
   - **Q10:** Were all patients included in the analysis?

   No applicability assessment is conducted for this domain.

Data extraction

Data extracted included the following: (1) study details: first author, publication year, research questions, study design; (2) patient details: the source of data acquisition (single-center/multicenter), sample size, smoking history, age, sex, TNM staging, treatment status (naïve or any treatment received prior image acquisition), histological subtype; (3) imaging details: imaging modality, plain or contrast CT; (4) oncogene mutation status-related information: type of mutation, specific subtype of mutation (if available), sequencing method; sequencing kit (5) radiomics details: segmentation software, type of segmentation (manual, automatic, or semi-automatic), radiomics feature extraction software, number of imaging features extracted, number and name of radiomics features included in final models, features selection methods, type of models constructed (machine learning [ML], deep learning [DL], classical statistical model), final classifier used in machine learning models, clinical variables included in the models (if applicable), and models performance. Two independent reviewers with more than 10 years of experience in biomedical research completed the initial screening and extracted data from all included studies.

Data analysis

The Reitsma method [6] has the distinct advantage of preserving the two-dimensional nature of the underlying data. It can also produce summary estimates of sensitivity and specificity (false positive rate [FPR, 1-specificity]), recognizing any possible correlation between these two measures. The method uses a random effect approach in which the values of the sensitivity and FPR estimates are obtained with restricted maximum likelihood.

The analyses were carried out by reproducing the confusion matrices of each model presented in the studies, the number of cases and the prevalence of oncogene mutant positive cases. All calculations were performed on the basis of validation cohorts for studies applying a training/validation split method, or on the basis of the total sample when cross-validation was the validation strategy. To ensure homogeneity, calculations were conducted based on internal validation cohort data when external validation was also performed (minority of the cases).

Predictor factors analyzed in the meta-regression: (1) average age of the cases, (2) manual segmentation vs semi-automatic segmentation vs both procedures (no studies including automatic segmentation approaches met the inclusion criteria for the quantitative analysis), (3) whether the model included only radiomics features or was combined with clinical variables, and (4) whether the model was classified as ML or DL. The heterogeneity in the description of the clinical variables included in the models prevented the inclusion of additional predictors of greatest clinical interest. Only the best model from each study according to its DOR was selected. When the mean/median age was not available due to the heterogeneity among studies when presenting descriptive results, it was inferred from the information obtained. Thus, mean and median values were indistinctly considered; when both values were provided, an average of both was calculated. If mean values were absent, median values were considered and viceversa. If both values were absent from the validation cohort, mean/median age from the total cohort was considered. When this information was not available either, the study was not included in the meta-regression.

References

1. Bossuyt PM, Reitsma JB (2003) The STARD initiative. Lancet 361:71.

2. Bossuyt PM, Reitsma JB, Bruns DEet al (2003) Towards complete and accurate reporting of studies of diagnostic accuracy: the STARD initiative. Radiology 226:24-28.
3. Bossuyt PM, Reitsma JB, Bruns DEet al (2015) STARD 2015: an updated list of essential items for reporting diagnostic accuracy studies. Radiology 277:826-832.
4. Cohen JF, Korevaar DA, Altman DGet al (2016) STARD 2015 guidelines for reporting diagnostic accuracy studies: explanation and elaboration. BMJ Open 6:e012799.
5. Mongan J, Moy L, Kahn CE (2020) Checklist for artificial intelligence in medical imaging (CLAIM): a guide for authors and reviewers. Radiol Artif Intell 2:e200029.
6. Reitsma JB, Glas AS, Rutjes AWet al (2005) Bivariate analysis of sensitivity and specificity produces informative summary measures in diagnostic reviews. J Clin Epidemiol 58:982-990.
7. Whiting PF, Rutjes AW, Westwood ME, et al. QUADAS-2: a revised tool for the quality assessment of diagnostic accuracy studies. Ann Intern Med 2011; 155(8):529–536.

Supplementary Figure S1. Hierarchical sROC curves of included studies for the comparative performance of radiomics models and combined models (radiomics + clinical data) using machine learning and/or deep learning methods for the prediction of EGFR mutation status ($n = 37$ and $n = 29$ studies, respectively). EGFR, epidermal growth factor receptor; sROC = summary receiver operating characteristic.

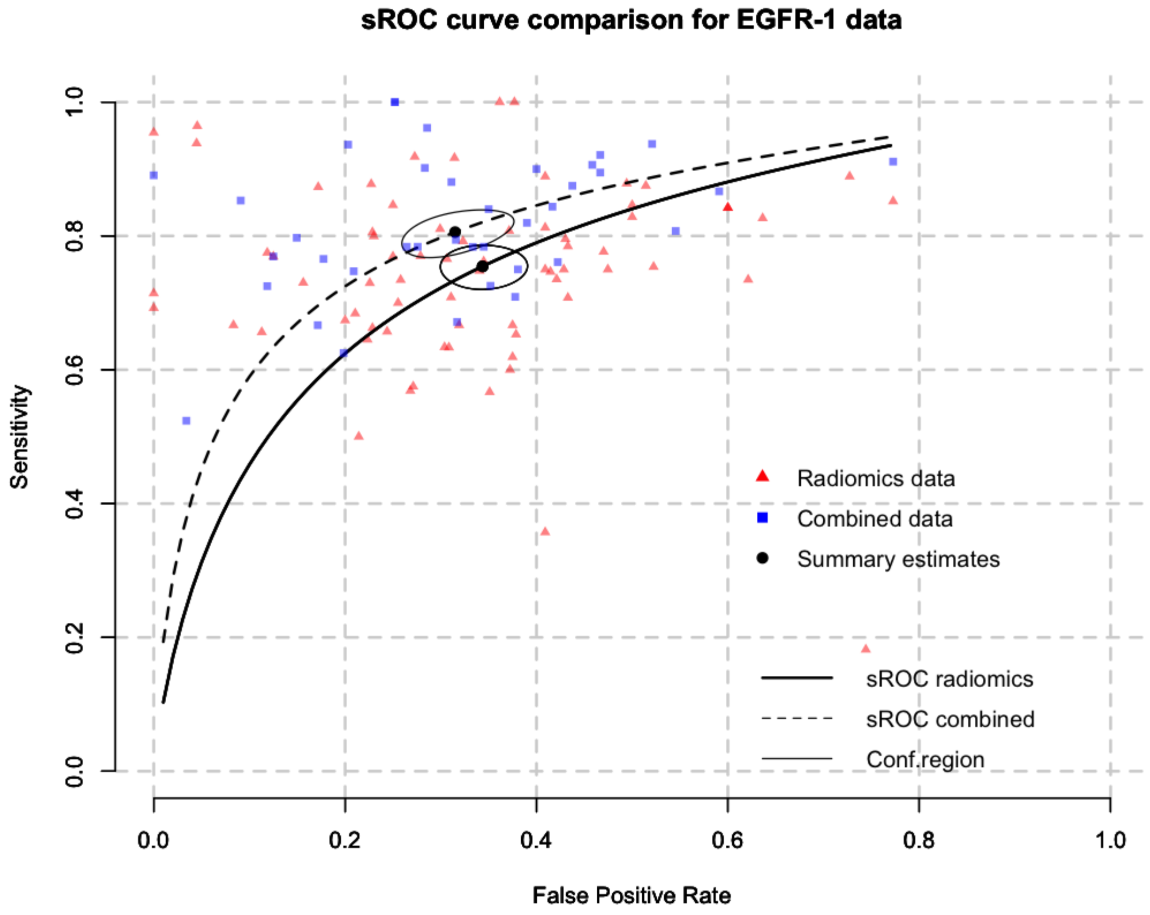

Supplementary Figure S2. Forest plots of the included studies developing combined models (radiomics + clinical data/ deep features + radiomic features) using machine learning and/or deep learning methods for the prediction of EGFR mutation status. Numbers are estimates with 95% CIs in brackets and indicated by horizontal lines. For those studies with the same name for the first author and published the same year, a hashtag was added to unequivocally tag them as done in Tables 1 and 2 and in the reference list. EGFR, epidermal growth factor receptor; CI, confidence interval; DOR, diagnostic odds ration; FPR, false positive rate.

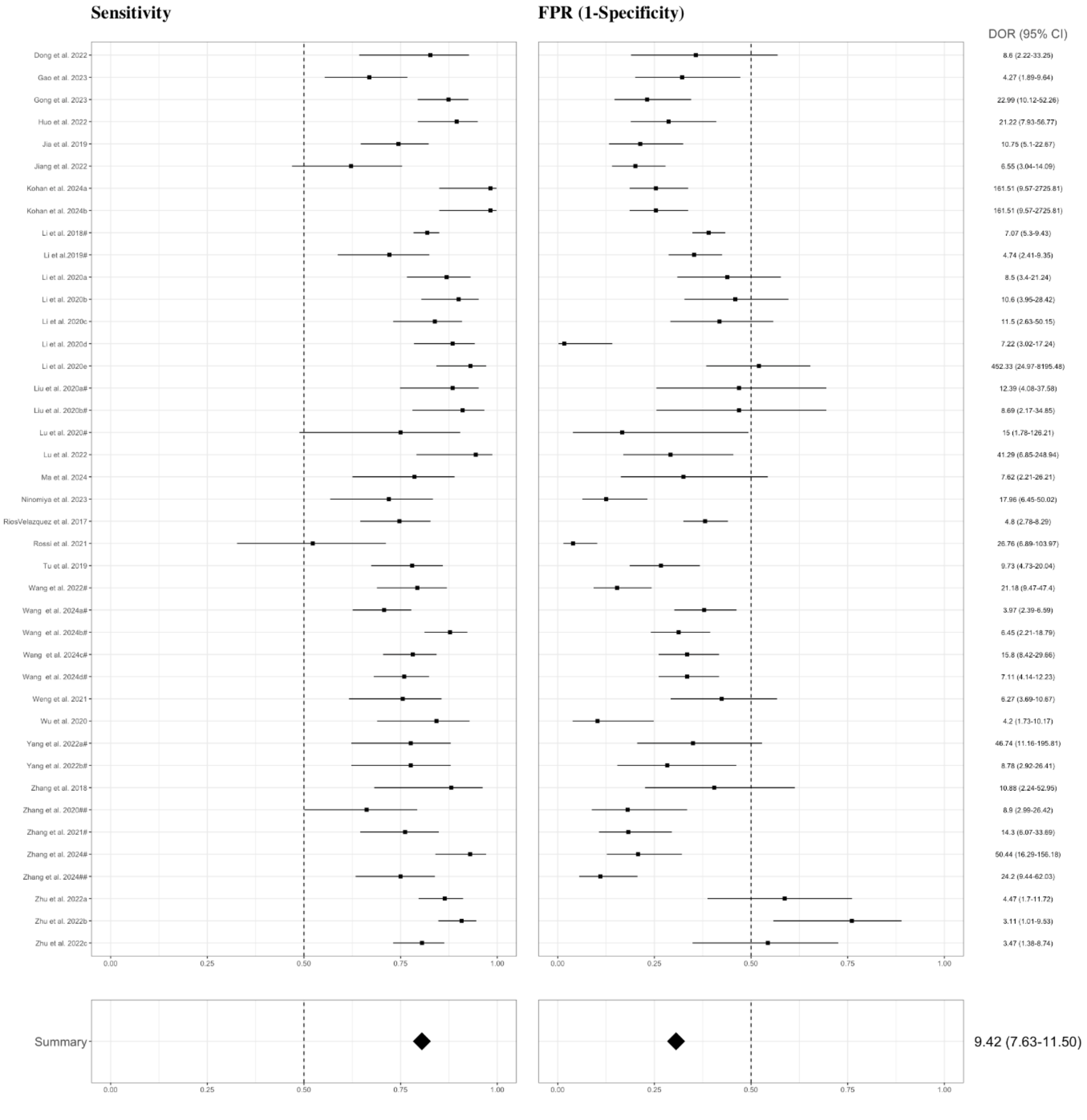

Supplementary Figure S3. Hierarchical sROC curve of included studies for the combined models (radiomics + clinical data/ deep features + radiomic features) using machine learning and/or deep learning methods for the prediction of EGFR mutation status.

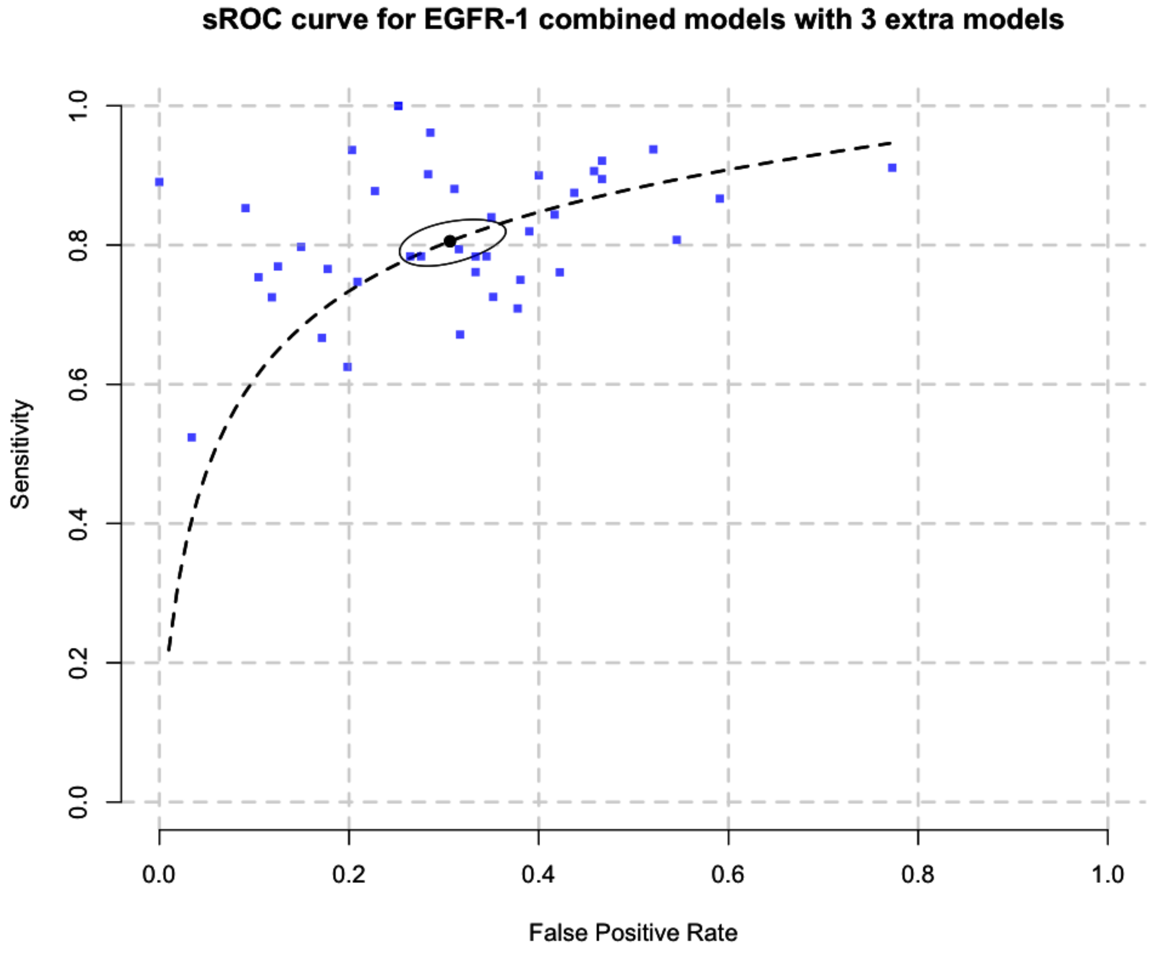

Supplementary Figure S4. Hierarchical sROC curve of included studies for the performance of radiomics models for the prediction of ALK mutation status (*n* = 3). ALK, anaplastic lymphoma kinase; sROC = summary receiver operating characteristic.

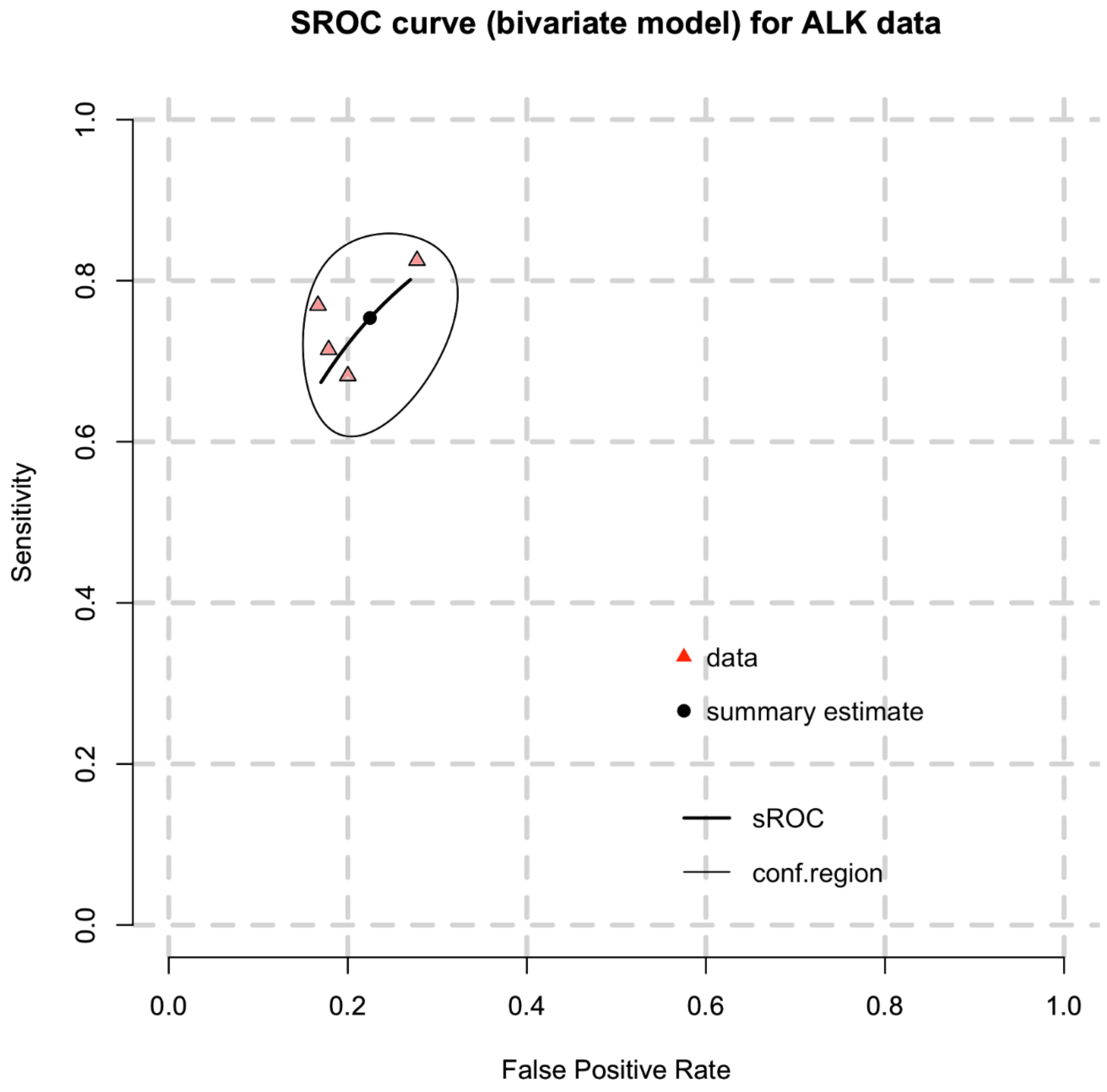

Supplementary Figure S5. Hierarchical sROC curve of included studies for the performance of radiomics models for the prediction of KRAS mutation status (*n* = 6). KRAS, Kirsten rat sarcoma viral oncogene homologue; sROC = summary receiver operating characteristic.

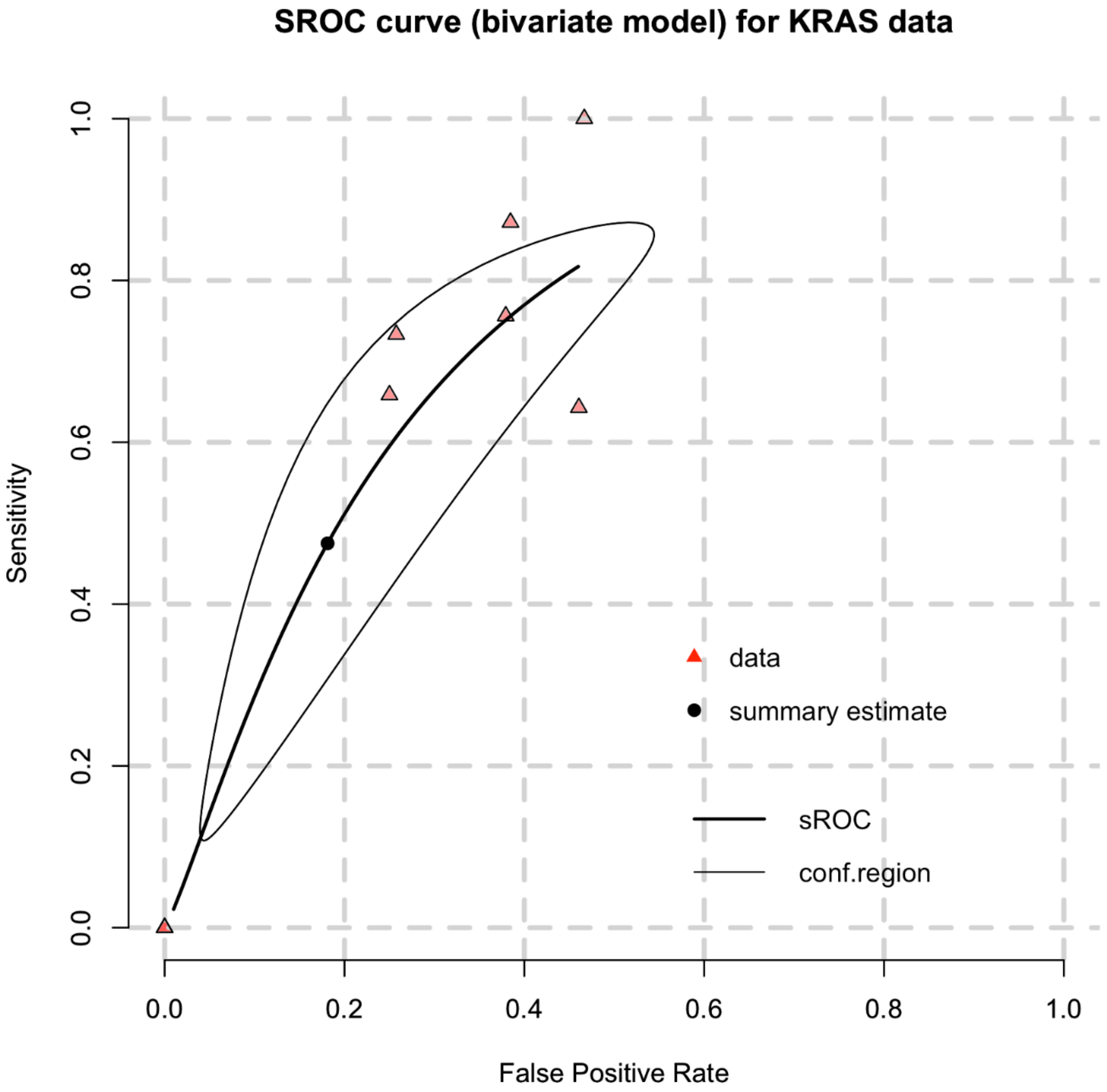

Supplementary Figure S6. Deeks' funnel plot for the assessment of publication bias in radiomics models for the prediction of EGFR mutation status ($n = 14$). EGFR, epidermal growth factor receptor.

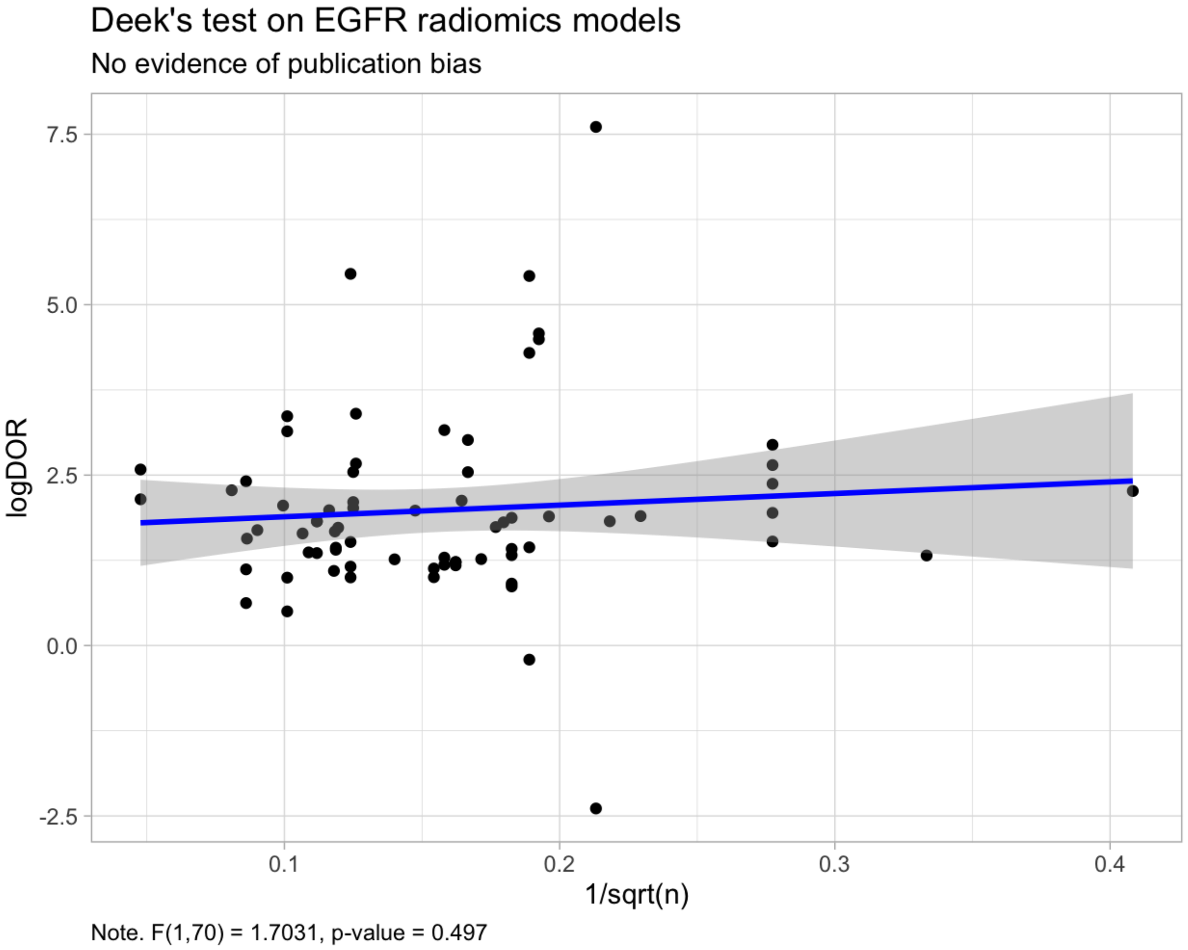

Supplementary Figure S7. Deeks' funnel plot for the assessment of publication bias in radiomics models for the prediction of ALK rearrangement status ($n = 5$). ALK, anaplastic lymphoma kinase.

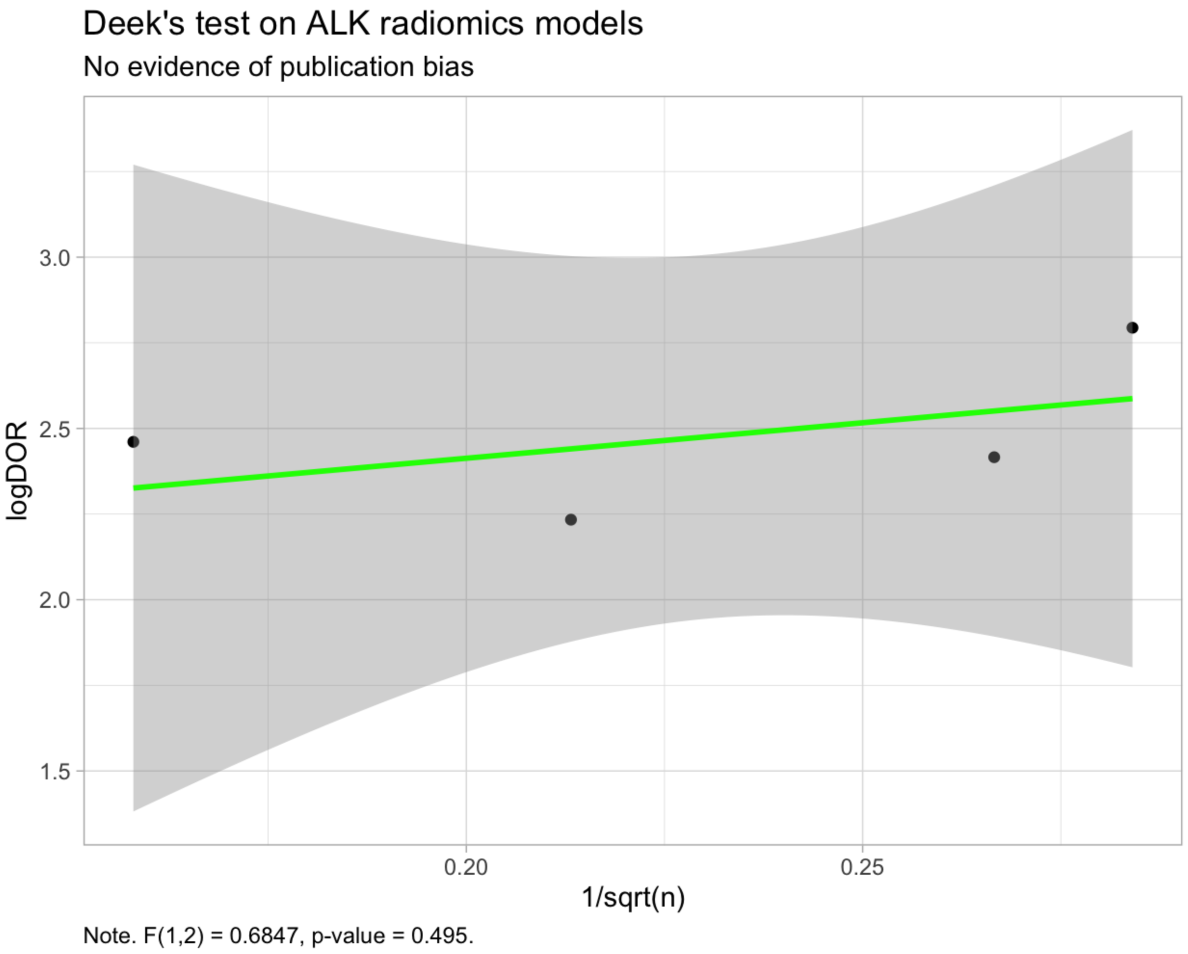

Supplementary Figure S8. Deeks' funnel plot for the assessment of publication bias in radiomics models for the prediction of KRAS mutation status ($n = 6$). KRAS, Kirsten rat sarcoma viral oncogene homologue.

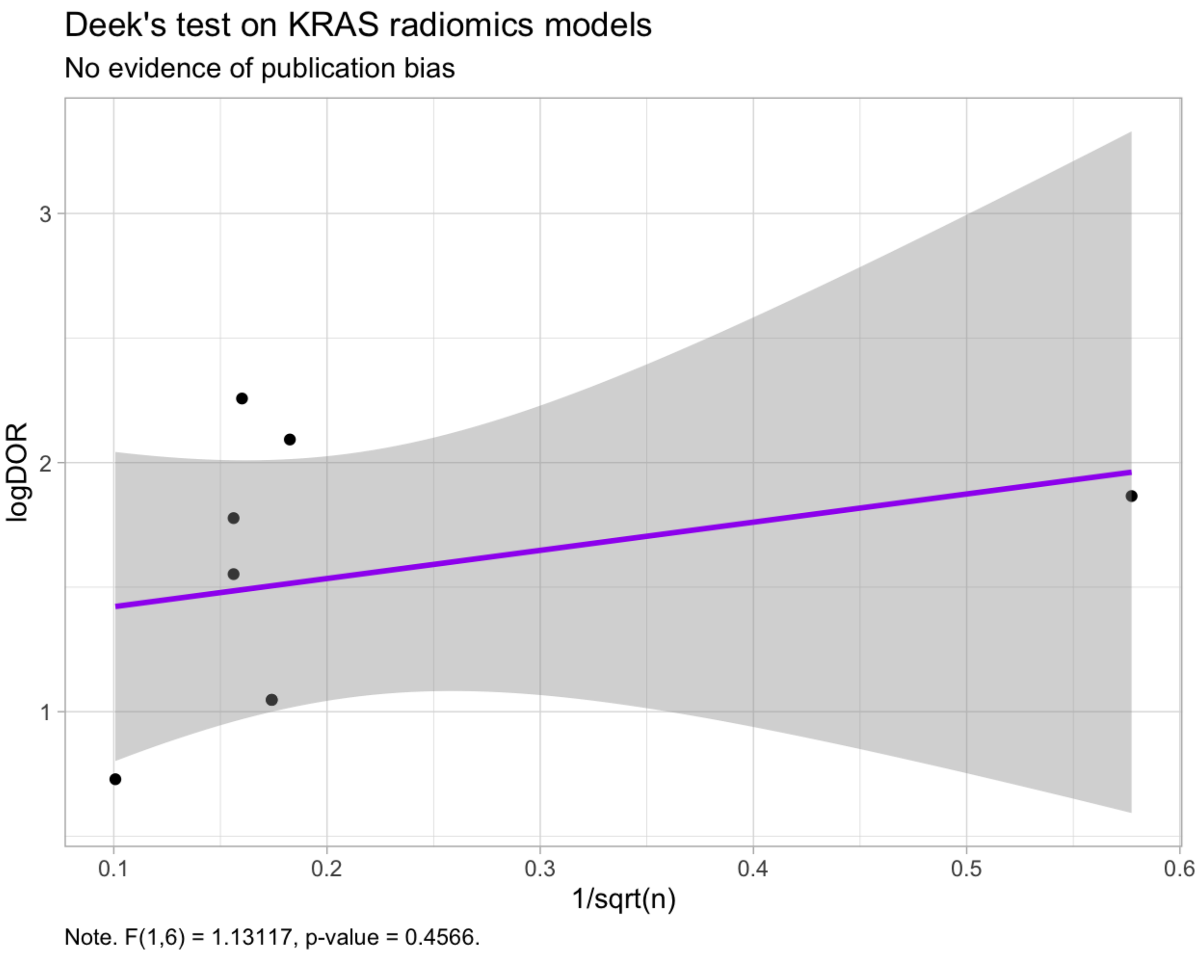

Deek's test on EGFR combined models

A slight evidence of publication bias

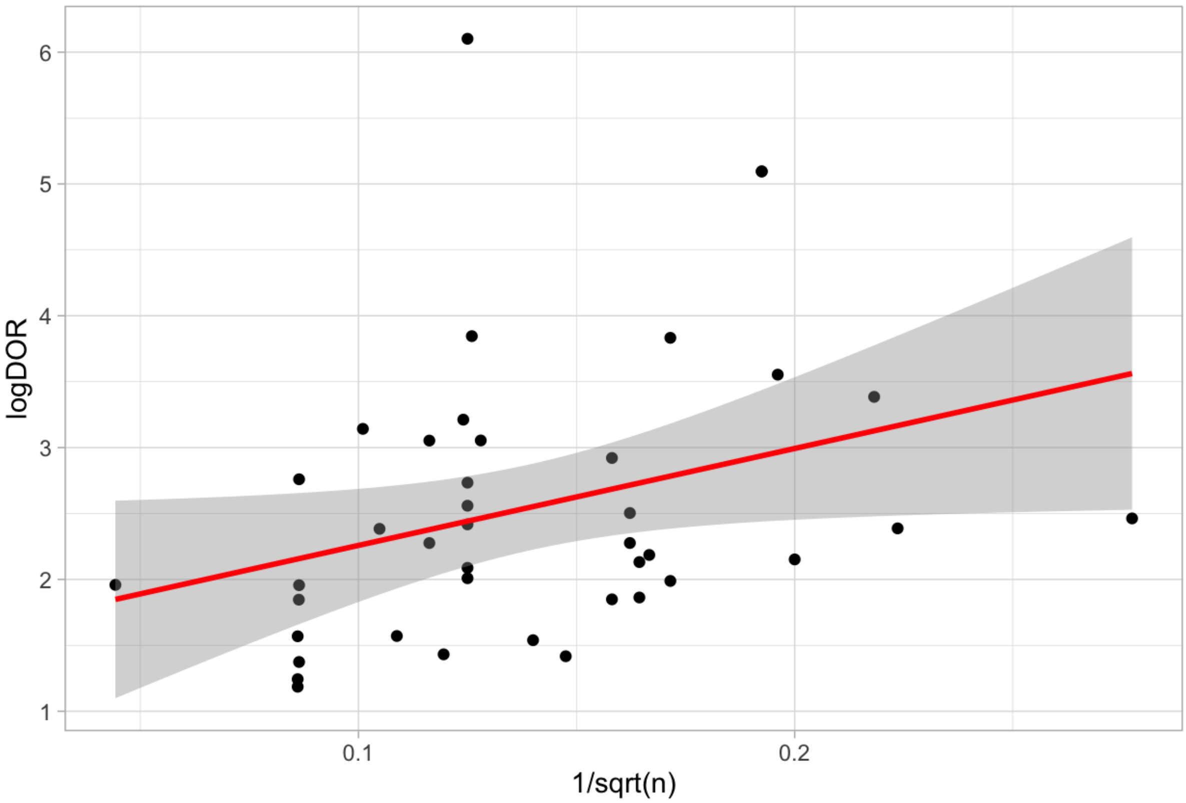

Note. F(1,39) = 4.385, p-value = 0.04281. When the analysis was repeated without the outlier, results were non-significant.

Supplementary Table S1. Search strategy applied for the qualitative analysis (systematic review).

Databases	Search strategy
MEDLINE (via Pubmed)	("radiomics"[TIAB] OR "radiomic"[TIAB] OR "texture analysis"[TIAB]) AND ("lung neoplasms"[MESH] OR "lung cancer"[TIAB] OR "NSCLC"[TIAB] or "non-small cell lung cancer"[TIAB] OR "lung adenocarcinoma"[TIAB]) AND ("mutational status" OR "mutation" OR "molecular subtype" OR "ALK"[TIAB] OR "anaplastic lymphoma kinase"[TIAB] OR "BRAF"[TIAB] OR "EGFR"[TIAB] OR "Epidermal growth factor receptor"[TIAB] OR "ERRB2"[TIAB] OR "Receptor, ErbB-2"[MESH] OR "HER2"[TIAB] OR "KRAS"[TIAB] OR "Kirsten rat sarcoma virus"[TIAB] OR "Proto Oncogene Proteins c met"[TIAB] OR "NTRK"[TIAB] OR "ROS"[TIAB] OR "c-ros"[TIAB])
COCHRANE LIBRARY	("radiomics" OR “radiomic” OR "texture analysis") AND ("lung neoplasms" OR "lung cancer" OR “NSCLC” OR “non-small cell lung cancer”) AND ("mutational status" OR "mutation" OR "molecular subtype" OR "ALK" OR "anaplastic lymphoma kinase" OR “BRAF” OR “EGFR” OR “ERRB2” OR "Receptor, ErbB-2" OR “HER2” OR “KRAS” OR “Kirsten rat sarcoma virus” OR “Proto Oncogene Proteins c met” OR “NTRK” OR “ROS” OR “c-ros”)
EMBASE	('radiomics':ab,ti OR 'radiomics'/exp OR 'radiomic':ab,ti OR 'texture analysis':ab,ti) AND ('lung cancer'/exp OR 'lung cancer':ab,ti OR

	**'NSCLC'/exp OR 'NSCLC':ab,ti OR 'non small cell lung cancer'/exp OR 'non small cell lung cancer':ab,ti OR 'lung adenocarcinoma'/exp OR 'lung adenocarcinoma':ab,ti) AND ('mutational status':ab,ti OR ('mutational' NEAR/2 'status') OR 'mutation':ab,ti OR 'mutation'/exp OR 'molecular subtype':ab,ti OR ('molecular' NEAR/2 'subtype') OR 'ALK':ab,ti OR 'ALK gene'/exp OR 'anaplastic lymphoma kinase':ab,ti OR 'anaplastic lymphoma kinase'/exp OR 'BRAF':ab,ti OR 'BRAF gene'/exp OR 'EGFR':ab,ti OR 'EGFR gene'/exp OR 'Epidermal growth factor receptor':ab,ti OR 'Epidermal growth factor receptor gene'/exp OR 'ERRB2':ab,ti OR 'ERRB2 gene'/exp OR 'epidermal growth factor receptor 2'/exp OR 'epidermal growth factor receptor 2':ab,ti OR 'HER2':ab,ti OR 'KRAS':ab,ti OR 'KRAS gene'/exp OR 'Kirsten rat sarcoma virus':ab,ti OR 'Kirsten rat sarcoma virus'/exp OR 'Proto Oncogene Proteins c met' OR 'MET':ab,ti OR 'MET gene'/exp OR 'NTRK':ab,ti OR 'NTRK gene'/exp OR 'c ros oncogene 1':ab,ti OR 'ROS1':ab,ti OR 'ROS1 gene'/exp)**

Supplementary Table S2. Quality assessment results obtained after CLAIM evaluation.

N	Study	Score			Mean score	Cut-off
		Reviewer 1 (A.J.P.)	Reviewer 2 (F.B.B.)	Reviewer 3 (A.P.P.)		
1	Chang et al. 2021 [1]	28	24	27	26	17
2	Chang et al. 2021 [2]	26	25	26	26	17
3	Cheng et al. 2023 [3]	22	24	24	23	17
4	Dong et al. 2021 [4]	23	22	19	21	18
5	Dong et al. 2022 [5]	25	22	21	23	18
6	Feng et al. 2022 [6]	20	22	23	22	18.5
7	Gao et al. 2023 [7]	22	22	20	21	17.5
8	Gong et al. 2023 [8]	26	26	26	26	18
9	Hinzpeter et al. 2024 [9]	21	18	21	20	17
10	Hu et al. 2024 [10]	25	22	23	23	17
11	Huo et al. 2022 [11]	22	25	25	24	17.5
12	Jia et al. 2019 [12]	19	19	20	19	17
13	Jiang et al. 2022 [13]	24	23	22	23	16.5
14	Kohan et al. 2024 [14]	22	18	21	20	17.2
15	Le et al. 2021 [15]	22	20	21	21	17.5
16	Li et al. 2018 [16]	25	28	22	25	19
17	Li et al. 2019 [17]	23	22	21	22	17
18	Li et al. 2020 [18]	25	23	22	23	19

**19**	**Li et al. 2022 [19]**	**21**	**21**	**21**	**21**	**17**
**20**	**Liu et al. 2020 [20]**	**22**	**25**	**23**	**23**	**17**
**21**	**Liu et al. 2022 [21]**	**24**	**23**	**22**	**23**	**17**
**22**	**Lu et al. 2020 [22]**	**27**	**30**	**26**	**28**	**17.5**
**23**	**Lu et al. 2022 [23]**	**22**	**25**	**21**	**23**	**17.5**
**24**	**Ma et al. 2020 [24]**	**24**	**26**	**22**	**24**	**17**
**25**	**Ma et al. 2024 [25]**	**21**	**21**	**22**	**21**	**17**
**26**	**Nair et al. 2021 [26]**	**21**	**22**	**19**	**21**	**17.5**
**27**	**Ninomiya et al. 2021 [27]**	**21**	**22**	**21**	**21**	**17.5**
**28**	**Ninomiya et al. 2023 [28]**	**21**	**22**	**22**	**22**	**17**
**29**	**Rios Velazquez et al. 2017 [29]**	**19**	**23**	**20**	**21**	**18**
**30**	**Rossi et al. 2021 [30]**	**20**	**22**	**19**	**20**	**17.5**
**31**	**Shao et al. 2024 [31]**	**29**	**27**	**25**	**27**	**18.5**
**32**	**Song et al. 2020 [32]**	**27**	**28**	**27**	**27**	**19**
**33**	**Tan et al. 2024 [33]**	**24**	**23**	**23**	**23**	**17**
**34**	**Tu et al. 2019 [34]**	**19**	**21**	**20**	**20**	**17**
**35**	**Wang et al. 2022 [35]**	**23**	**26**	**26**	**25**	**18**
**36**	**Wang et al. 2022 [36]**	**24**	**22**	**22**	**23**	**19**
**37**	**Wang et al. 2024 [37]**	**21**	**20**	**20**	**20**	**17**
**38**	**Wang et al. 2025 [38]**	**27**	**26**	**25**	**26**	**18.5**
**39**	**Weng et al. 2021 [39]**	**24**	**25**	**24**	**24**	**17.5**
**40**	**Wu 2020 [40]**	**20**	**22**	**21**	**21**	**17**
**41**	**Wu et al. 2024 [41]**	**26**	**26**	**25**	**26**	**18.7**
**42**	**Yang 2020 [42]**	**22**	**23**	**23**	**23**	**17**

43	Yang 2022 [43]	19	18	18	18	17
44	Zhang 2018 [44]	26	27	23	25	17
45	Zhang 2020 [45]	19	19	19	19	17
46	Zhang 2020 [46]	22	23	23	23	17
47	Zhang 2021 [47]	27	26	26	26	17
48	Zhang et al. 2024 [48]	24	25	24	24	17
49	Zhang et al. 2024 [49]	25	27	23	25	18.3
50	Zhao 2022 [50]	23	24	23	23	19
51	Zhu 2022 [51]	22	22	20	21	17.5

Supplementary Table S3. Geographic origin of patient datasets included in the systematic review.

Countries	Number of studies
Canada	3
China	87
China / Japan	1
China / USA	5
France	1
Germany	1
Greece	1
India	1
Indonesia	1
Iran	1
Iran / Switzerland / Canada / Netherlands /UK	1
Italy	3
Japan	5
Malaysia / Japan / USA	1
South Korea	2
USA	9

Supplementary Table S4. Risk of bias and applicability concerns for each included study based on the QUADAS-2 assessment tool. The table summarizes the responses to all QUADAS-2 domains for the 124 included studies. Signaling questions are abbreviated as "Q" and applicability concerns as "A". Each row corresponds to a single study.

Study	Patient selection				Index test			Reference standard			Flow and timing		
Agüloğlu et al. 2022 [52]	Q1	Q2	Q3	A1	Q4	Q5	A2	Q6	Q7	A3	Q8	Q9	Q10
Aerts et al. 2016 [53]	Yes	Yes	Yes	No	Unclear	No	No	Yes	Unclear	No	Yes	Yes	Yes
Agazzi et al. 2021 [54]	Yes	Yes	Yes	No	Unclear	Yes	No	Yes	Unclear	No	Yes	Yes	Yes
Aide et al. 2022 [55]	Yes	Yes	Yes	No	Yes	Yes	No	Yes	Unclear	No	Yes	Yes	Yes
Chang et al. 2021 [1]	Yes	Yes	Yes	No	Unclear	Yes	No	Yes	Unclear	No	Yes	Yes	Yes
Chang et al. 2021 [2]	Yes	Yes	Yes	No	Yes	Yes	No	Yes	Unclear	No	Yes	Yes	Yes
Chen et al. 2021 [56]	Yes	Yes	Yes	No	Yes	Yes	No	Yes	Unclear	No	Yes	Yes	Yes
Chen et al. 2022 [57]	Yes	Yes	Yes	No	Yes	Yes	No	Yes	Unclear	No	Yes	Yes	Yes
Chen et al. 2024 [58]	Yes	Yes	Yes	No	Yes	Yes	No	Yes	Unclear	No	Yes	Yes	Yes
Cheng et al. 2023 [3]	Yes	Yes	Yes	No	Yes	Yes	No	Yes	Unclear	No	Yes	Yes	Yes
Choe et al. 2021 [59]	Yes	Yes	Yes	No	Yes	Yes	No	Yes	Unclear	No	Yes	Yes	Yes

Dang et al. 2021 [60]	Yes	Yes	Yes	No	Yes	Yes	No	Yes	Unclear	No	Yes	Yes	Yes
Digumarthy et al. 2019 [61]	Yes	Yes	Yes	No	Yes	Yes	No	Yes	Unclear	No	Yes	Yes	Yes
Dong et al. 2022 [5]	Yes	Yes	Yes	No	Yes	Yes	No	Yes	Unclear	No	Yes	Yes	Yes
Dong et al. 2021 [4]	Yes	Yes	Yes	No	Yes	Yes	No	Yes	Unclear	No	Yes	Yes	Yes
Feng et al. 2022 [6]	Yes	Yes	Yes	No	Yes	Yes	No	Yes	Unclear	No	Yes	Yes	Yes
Gao et al. 2023 [7]	Yes	Yes	Yes	No	Yes	Yes	No	Yes	Unclear	No	Yes	Yes	Yes
Gong et al. 2023 [8]	Yes	Yes	Yes	No	Yes	Yes	No	Yes	Unclear	No	Yes	Yes	Yes
Hao et al. 2022 [62]	Yes	Yes	Yes	No	Yes	Yes	No	Yes	Unclear	No	Yes	Yes	Yes
He et al. 2022 [63]	Yes	Yes	Yes	No	Yes	Yes	No	Yes	Unclear	No	Yes	Yes	Yes
Hinzpeter et al. 2024 [9]	Yes	Yes	Yes	No	Yes	Yes	No	Yes	Unclear	No	Yes	Yes	Yes
Hong et al. 2020 [64]	Yes	Yes	Yes	No	Yes	Yes	No	Yes	Unclear	No	Yes	Yes	Yes
Hu et al. 2024 [10]	Yes	Yes	Yes	No	Yes	Yes	No	Yes	Unclear	No	Yes	Yes	Yes
Huang et al. 2018 [65]	Yes	Yes	Yes	No	Yes	Yes	No	Yes	Unclear	No	Yes	Yes	Yes
Huang et al. 2022 [66]	Yes	Yes	Yes	No	Unclear	Yes	No	Yes	Unclear	No	Yes	Yes	Yes
Huang et al. 2022 [67]	Yes	Yes	Yes	No	Yes	Yes	No	Yes	Unclear	No	Yes	Yes	Yes

**Huo et al. 2022 [11]**	**Yes**	**Yes**	**Yes**	**No**	**Yes**	**Yes**	**No**	**Yes**	**Unclear**	**No**	**Yes**	**Yes**	**Yes**
**Hou et al. 2021 [68]**	**Yes**	**Yes**	**Yes**	**No**	**Yes**	**Yes**	**No**	**Yes**	**Unclear**	**No**	**Yes**	**Yes**	**Yes**
**Jia et al. 2019 [12]**	**Yes**	**Yes**	**Yes**	**No**	**Yes**	**Yes**	**No**	**Yes**	**Unclear**	**No**	**Yes**	**Yes**	**Yes**
**Jiang et al. 2019 [69]**	**Yes**	**Yes**	**Yes**	**No**	**Yes**	**Yes**	**No**	**Yes**	**Unclear**	**No**	**Yes**	**Yes**	**Yes**
**Jiang et al. 2022 [13]**	**Yes**	**Yes**	**Yes**	**No**	**Yes**	**Yes**	**No**	**Yes**	**Unclear**	**No**	**Yes**	**Yes**	**Yes**
**Kawazoe et al. 2023 [70]**	**Yes**	**Yes**	**Yes**	**No**	**Yes**	**Yes**	**No**	**Yes**	**Unclear**	**No**	**Yes**	**Yes**	**Yes**
**Kawazoe et al. 2023 [71]**	**Yes**	**Yes**	**Yes**	**No**	**Yes**	**Yes**	**No**	**Yes**	**Unclear**	**No**	**Yes**	**Yes**	**Yes**
**Kim et al. 2024 [72]**	**Yes**	**Yes**	**Yes**	**No**	**Yes**	**Yes**	**No**	**Yes**	**Unclear**	**No**	**Yes**	**Yes**	**Yes**
**Kohan et al. 2024 [14]**	**Yes**	**Yes**	**Yes**	**No**	**Yes**	**Yes**	**No**	**Yes**	**Unclear**	**No**	**Yes**	**Yes**	**Yes**
**Koyasu et al. 2020 [73]**	**Yes**	**Yes**	**Yes**	**No**	**Yes**	**Yes**	**No**	**Yes**	**Unclear**	**No**	**Yes**	**Yes**	**Yes**
**Le et al. 2021 [15]**	**Yes**	**Yes**	**Yes**	**No**	**Yes**	**Yes**	**No**	**Yes**	**Unclear**	**No**	**Yes**	**Yes**	**Yes**
**Li et al. 2018 [74]**	**Yes**	**Yes**	**Yes**	**No**	**Unclear**	**Yes**	**No**	**Yes**	**Unclear**	**No**	**Yes**	**Yes**	**Yes**
**Li et al. 2018 [16]**	**Yes**	**Yes**	**Yes**	**No**	**Unclear**	**Yes**	**No**	**Yes**	**Unclear**	**No**	**Yes**	**Yes**	**Yes**
**Li et al 2019 [75]**	**Yes**	**Yes**	**Yes**	**No**	**Yes**	**Yes**	**No**	**Yes**	**Unclear**	**No**	**Yes**	**Yes**	**Yes**
**Li et al. 2019 [17]**	**Yes**	**Yes**	**Yes**	**No**	**Yes**	**Yes**	**No**	**Yes**	**Unclear**	**No**	**Yes**	**Yes**	**Yes**

Li et al. 2020 [18]	Yes	Yes	Yes	No	Yes	Yes	No	Yes	Unclear	No	Yes	Yes	Yes
Li et al. 2021 [76]	Yes	Yes	Yes	No	Yes	Yes	No	Yes	Unclear	No	Yes	Yes	Yes
Li et al. 2022 [19]	Yes	Yes	Yes	No	Yes	Yes	No	Yes	Unclear	No	Yes	Yes	Yes
Li et al. 2023 [77]	Yes	Yes	Yes	No	Yes	Yes	No	Yes	Unclear	No	Yes	Yes	Yes
Li et al. 2025 [78]	Yes	Yes	Yes	No	Yes	Yes	No	Yes	Unclear	No	Yes	Yes	Yes
Liu et al. 2016 [79]	Yes	Yes	Yes	No	Yes	Yes	no	Yes	Unclear	No	Yes	Yes	Yes
Liu et al. 2020 [80]	Yes	Yes	Yes	No	Yes	Yes	No	Yes	Unclear	No	Yes	Yes	Yes
Liu et al. 2020 [20]	Yes	Yes	Yes	No	Yes	Yes	No	Yes	Unclear	No	Yes	Yes	Yes
Liu et al. 2022 [21]	Yes	Yes	Yes	No	Yes	Yes	No	Yes	Unclear	No	Yes	Yes	Yes
Liu et al. 2023 [81]	Yes	Yes	Yes	No	Yes	Yes	No	Yes	Unclear	No	Yes	Yes	Yes
Liu et al. 2023 [82]	Yes	Yes	Yes	No	Yes	Yes	No	Yes	Unclear	No	Yes	Yes	Yes
Lu et al. 2020 [83]	Yes	Yes	Yes	No	Yes	Yes	No	Yes	Unclear	No	Yes	Yes	Yes
Lu et al. 2020 [22]	Yes	Yes	Yes	No	Yes	Yes	No	Yes	Unclear	No	Yes	Yes	Yes
Lu et al. 2022 [23]	Yes	Yes	Yes	No	Yes	Yes	No	Yes	Unclear	No	Yes	Yes	Yes
Lu et al. 2024 [84]	Yes	Yes	Yes	No	Yes	Yes	No	Yes	Unclear	No	Yes	Yes	Yes

**Ma et al. 2020 [24]**	**Yes**	**Yes**	**Yes**	**No**	**Yes**	**Yes**	**No**	**Yes**	**Unclear**	**No**	**Yes**	**Yes**	**Yes**
**Ma et al. 2024 [25]**	**Yes**	**Yes**	**Yes**	**No**	**Yes**	**Yes**	**No**	**Yes**	**Unclear**	**No**	**Yes**	**Yes**	**Yes**
**Mahajan et al. 2024 [85]**	**Yes**	**Yes**	**Yes**	**No**	**Yes**	**Yes**	**No**	**Yes**	**Unclear**	**No**	**Yes**	**Yes**	**Yes**
**Mei et al. 2018 [86]**	**Yes**	**Yes**	**Yes**	**No**	**Yes**	**Yes**	**No**	**Yes**	**Unclear**	**No**	**Yes**	**Yes**	**Yes**
**Mu et al. 2020 [87]**	**Yes**	**Yes**	**Yes**	**No**	**Yes**	**Yes**	**No**	**Yes**	**Unclear**	**No**	**Yes**	**Yes**	**Yes**
**Nair et al. 2021 [26]**	**Yes**	**Yes**	**Yes**	**No**	**Yes**	**Yes**	**No**	**Yes**	**Unclear**	**No**	**Yes**	**Yes**	**Yes**
**Ninomiya et al. 2021 [27]**	**Yes**	**Yes**	**Yes**	**No**	**Yes**	**Yes**	**No**	**Yes**	**Unclear**	**No**	**Yes**	**Yes**	**Yes**
**Ninomiya et al. 2023 [28]**	**Unclear**	**Yes**	**Unclear**	**No**	**Unclear**	**Yes**	**No**	**Yes**	**Unclear**	**No**	**Unclear**	**Yes**	**Yes**
**Njoto et al. 2023 [88]**	**Yes**	**Yes**	**Yes**	**No**	**Unclear**	**Yes**	**No**	**Yes**	**Unclear**	**No**	**Unclear**	**Yes**	**Yes**
**Omura et al. 2023 [89]**	**No**	**No**	**Unclear**	**No**	**Unclear**	**Yes**	**No**	**Yes**	**Unclear**	**No**	**Unclear**	**Yes**	**Yes**
**Ottaiano et al. 2024 [90]**	**Yes**	**Yes**	**Yes**	**No**	**Unclear**	**Yes**	**No**	**Yes**	**Unclear**	**No**	**Unclear**	**Yes**	**Yes**
**Rinaldi et al. 2023 [91]**	**Yes**	**Yes**	**Yes**	**No**	**Unclear**	**Yes**	**No**	**Yes**	**Unclear**	**No**	**Yes**	**Yes**	**Yes**
**Ríos Velázquez et al. 2017 [29]**	**Yes**	**Yes**	**Yes**	**No**	**Unclear**	**Yes**	**No**	**Yes**	**Unclear**	**No**	**Yes**	**Yes**	**Yes**
**Rossi et al. 2021 [30]**	**Yes**	**Yes**	**Yes**	**No**	**Unclear**	**Yes**	**No**	**Yes**	**Unclear**	**No**	**Yes**	**Yes**	**Yes**

Ruan et al. 2022 [92]	Yes	Yes	Yes	No	Yes	Yes	No	Yes	Unclear	No	Yes	Yes	Yes
Shang et al. 2023 [93]	Yes	Yes	Yes	No	Unclear	Yes	No	Yes	Unclear	No	Yes	Yes	Yes
Shao et al. 2022 [94]	Yes	Yes	Yes	No	Unclear	Yes	No	Unclear	Unclear	No	Yes	Yes	Yes
Shao et al. 2024 [31]	Yes	Yes	Yes	No	Unclear	Yes	No	Yes	Unclear	No	Yes	Yes	Yes
Shiri et al. 2020 [95]	Yes	Yes	Yes	No	Unclear	Yes	No	Yes	Unclear	No	Yes	Yes	Yes
Shiri et al. 2022 [96]	Yes	Yes	Yes	No	Unclear	Yes	No	Yes	Unclear	No	Yes	Yes	Yes
Song et al. 2021 [97]	Yes	Yes	Yes	No	Unclear	Yes	No	Yes	Unclear	No	Yes	Yes	Yes
Song et al. 2020 [32]	Yes	Yes	Yes	No	Unclear	Yes	No	Yes	Unclear	No	Yes	Yes	Yes
Tan et al. 2023 [33]	Yes	Yes	Yes	No	Yes	Yes	No	Yes	Unclear	No	Yes	Yes	Yes
Trivizakis et al. 2021 [98]	Yes	Yes	Yes	No	Yes	Yes	No	Yes	Unclear	No	Yes	Yes	Yes
Tu et al. 2019 [34]	Yes	Yes	Yes	No	Unclear	Yes	No	Yes	Unclear	No	Yes	Yes	Yes
Wang et al. 2019 [99]	Yes	Yes	Yes	No	Unclear	Yes	No	Yes	Unclear	No	Yes	Yes	Yes
Wang et al. 2021 [100]	Yes	Yes	Yes	No	Unclear	Yes	No	Yes	Unclear	No	Yes	Yes	Yes
Wang et al. 2022 [35]	Yes	Yes	Yes	No	Unclear	Yes	No	Yes	Unclear	No	Yes	Yes	Yes
Wang et al. 2022 [36]	Yes	Yes	Yes	No	Unclear	Yes	No	Yes	Unclear	No	Yes	Yes	Yes

**Wang et al. 2024 [37]**	**Yes**	**Yes**	**Yes**	**No**	**Unclear**	**Yes**	**No**	**Yes**	**Unclear**	**No**	**Yes**	**Yes**	**Yes**
**Wang et al. 2025 [38]**	**Yes**	**Yes**	**Yes**	**No**	**Unclear**	**Yes**	**No**	**Yes**	**Unclear**	**No**	**Yes**	**Yes**	**Yes**
**Wang et al. 2024 [101]**	**Yes**	**Yes**	**Yes**	**No**	**Yes**	**Yes**	**No**	**Yes**	**Unclear**	**No**	**Yes**	**Yes**	**Yes**
**Wang et al. 2024 [102]**	**Yes**	**Yes**	**Yes**	**No**	**Yes**	**Yes**	**No**	**Yes**	**Unclear**	**No**	**Yes**	**Yes**	**Yes**
**Weng et al. 2021 [39]**	**Yes**	**Yes**	**Yes**	**No**	**Yes**	**Yes**	**No**	**Yes**	**Unclear**	**No**	**Yes**	**Yes**	**Yes**
**Wu et al. 2020 [40]**	**Yes**	**Yes**	**Yes**	**No**	**Unclear**	**Yes**	**No**	**Yes**	**Unclear**	**No**	**Yes**	**Yes**	**Yes**
**Wu et al. 2024 [41]**	**Yes**	**Yes**	**Yes**	**No**	**Yes**	**Yes**	**No**	**Yes**	**Unclear**	**No**	**Yes**	**Yes**	**Yes**
**Xiao et al. 2023 [103]**	**Yes**	**Yes**	**Yes**	**No**	**Unclear**	**Yes**	**No**	**Yes**	**Unclear**	**No**	**Yes**	**Yes**	**Yes**
**Xiong et al. 2024 [104]**	**Yes**	**Yes**	**Yes**	**No**	**Unclear**	**Yes**	**No**	**Yes**	**Unclear**	**No**	**Yes**	**Yes**	**Yes**
**Xu et al. 2024 [105]**	**Yes**	**Yes**	**Yes**	**No**	**Unclear**	**Yes**	**No**	**Yes**	**Unclear**	**No**	**Yes**	**Yes**	**Yes**
**Yamazaki et al. 2022 [106]**	**Yes**	**Yes**	**Yes**	**No**	**Unclear**	**Yes**	**No**	**Yes**	**Unclear**	**No**	**Yes**	**Yes**	**Yes**
**Yang et al. 2020 [42]**	**Yes**	**Yes**	**Yes**	**No**	**Unclear**	**Yes**	**No**	**Yes**	**Unclear**	**No**	**Yes**	**Yes**	**Yes**
**Yang et al. 2020 [107]**	**Yes**	**Yes**	**Yes**	**No**	**Unclear**	**Yes**	**No**	**Yes**	**Unclear**	**No**	**Yes**	**Yes**	**Yes**
**Yang et al. 2022 [43]**	**Yes**	**Yes**	**Yes**	**No**	**Unclear**	**Yes**	**No**	**Yes**	**Unclear**	**No**	**Yes**	**Yes**	**Yes**
**Yang et al. 2022 [108]**	**Yes**	**Yes**	**Yes**	**No**	**Unclear**	**Yes**	**No**	**Yes**	**Unclear**	**No**	**Yes**	**Yes**	**Yes**

Yang et al. 2022 [109]	Yes	Yes	Yes	No	Unclear	Yes	No	Yes	Unclear	No	Yes	Yes	Yes
Yao et al. 2024 [110]	Yes	Yes	Yes	No	Yes	Yes	No	Yes	Unclear	No	Yes	Yes	Yes
Yip et al. 2017 [111]	Yes	Yes	Yes	No	Unclear	Yes	No	Yes	Unclear	No	Yes	Yes	Yes
Zhang et al. 2018 [44]	Yes	Yes	Yes	No	Unclear	Yes	No	Yes	Unclear	No	Yes	Yes	Yes
Zhang et al. 2020 [45]	Yes	Yes	Yes	No	Unclear	Yes	No	Yes	Unclear	No	Yes	Yes	Yes
Zhang et al. 2020 [112]	Yes	Yes	Yes	No	Yes	Yes	No	Yes	Unclear	No	Yes	Yes	Yes
Zhang et al. 2020 [46]	Yes	Yes	Yes	No	Unclear	Yes	No	Yes	Unclear	No	Yes	Yes	Yes
Zhang et al. 2021 [113]	Yes	Yes	Yes	No	Unclear	No	No	Yes	Unclear	No	Yes	Yes	Yes
Zhang et al. 2021 [47]	Yes	Yes	Yes	No	Yes	Yes	No	Yes	Unclear	No	Yes	Yes	Yes
Zhang et al. 2023 [114]	Yes	Yes	Yes	No	Yes	Yes	No	Yes	Unclear	No	Yes	Yes	Yes
Zhang et al. 2023 [115]	Yes	Yes	Yes	No	Unclear	Yes	No	Yes	Unclear	No	Yes	Yes	Yes
Zhang et al. 2024 [48]	Yes	Yes	Yes	No	Yes	Yes	No	Yes	Unclear	No	Yes	Yes	Yes
Zhang et al. 2024 [49]	Yes	Yes	Yes	No	Unclear	Yes	No	Yes	Unclear	No	Yes	Yes	Yes
Zhang et al. 2024 [116]	Yes	Yes	Yes	No	Yes	Yes	No	Yes	Unclear	No	Yes	Yes	Yes
Zhang et al. 2024 [117]	Yes	Yes	Yes	No	Yes	Yes	No	Yes	Unclear	No	Yes	Yes	Yes

**Zhao et al. 2019 [118]**	**Yes**	**Yes**	**Yes**	**No**	**Unclear**	**Yes**	**No**	**Yes**	**Unclear**	**No**	**Yes**	**Yes**	**Yes**
**Zhao et al. 2019 [119]**	**Yes**	**Yes**	**Yes**	**No**	**Yes**	**Yes**	**No**	**Yes**	**Unclear**	**No**	**Yes**	**Yes**	**Yes**
**Zhao et al. 2022 [50]**	**Yes**	**Yes**	**Yes**	**No**	**Unclear**	**Yes**	**No**	**Yes**	**Unclear**	**No**	**Yes**	**Yes**	**Yes**
**Zhao et al. 2024 [120]**	**Yes**	**Yes**	**yes**	**No**	**Unclear**	**yes**	**no**	**yes**	**unclear**	**No**	**yes**	**Yes**	**Yes**
**Zhu et al. 2022 [51]**	**Yes**	**Yes**	**yes**	**No**	**Unclear**	**yes**	**no**	**yes**	**unclear**	**No**	**yes**	**Yes**	**Yes**
**Zhu et al. 2021 [121]**	**Yes**	**Yes**	**yes**	**No**	**Unclear**	**yes**	**no**	**yes**	**unclear**	**No**	**yes**	**Yes**	**Yes**
**Zuo et al. 2023 [122]**	**Yes**	**Yes**	**yes**	**No**	**Yes**	**yes**	**no**	**yes**	**unclear**	**No**	**yes**	**Yes**	**Yes**
**Zuo et al. 2024 [123]**	**Yes**	**Yes**	**yes**	**No**	**Unclear**	**yes**	**no**	**yes**	**unclear**	**No**	**yes**	**Yes**	**Yes**
**Zuo et al. 2024 [124]**	**Yes**	**Yes**	**yes**	**No**	**Unclear**	**yes**	**no**	**yes**	**unclear**	**No**	**yes**	**Yes**	**Yes**
	**Yes**	**Yes**	**yes**	**No**	**Yes**	**yes**	**no**	**yes**	**unclear**	**No**	**yes**	**Yes**	**Yes**

Supplementary Table S5. Studies included in the different meta-analyses conducted.

N	EGFR		ALK	KRAS
	**Radiomics models**	**Combined models**	**Radiomics models**	**Radiomics models**
1	Chang et al. 2021 [1]	Dong et al. 2022 [4]	Chang et al. 2021 [2]	Dong et al. 2021**†† [4]
2	Cheng et al. 2023 [3]	Gao et al. 2023 [7]	Ma et al. 2020¶ [24]	Hinzpeter et al. 2024** [9]
3	Dong et al. 2021*† [4]	Gong et al. 2023 [8]	Song et al. 2020 [32]	Kohan et al. 2024†** [14]
4	Feng et al. 2022† [6]	Kohan et al. 2024 [14]		Le et al. 2021** [15]
5	Gao et al. 2023 [6]	Huo et al. 2022 [11]		Rios Velazquez et al. 2017** [29]
6	Gong et al. 2023†‡ [8]	Jia et al. 2019 [12]		Wang et al. 2022 [36]
7	Hinzpeter et al. 2024† [9]	Jiang et al. 2022 [13]		
8	Hu et al. 2024 [10]	Li et al. 2018 [16]		
9	Kohan et al. 2024† [14]	Li et al. 2019 [17]		
10	Le et al. 2021 [15]	Li et al. 2020† [18]		
11	Li et al. 2018[16]	Liu et al. 2020† [20]		

12	Li et al. 2019 [17]	Lu et al. 2020 [22]		
13	Li et al. 2022 [19]	Lu et al. 2022 [23]		
14	Liu et al. 2020† [20]	Ma et al. 2024 [25]		
15	Liu et al. 2022† [21]	Ninomiya et al. 2023 [28]		
16	Lu et al. 2020 [22]	Rios Velazquez et al. 2017 [29]		
17	Lu et al. 2022 [23]	Rossi et al. 2021 [30]		
18	Ma et al. 2024 [25]	Tu et al. 2019 [34]		
19	Nair et al. 2021 [26]	Wang et al. 2022 [35]		
20	Ninomiya et al. 2021† [27]	Wang et al. 2025† [38]		
21	Rios Velazquez et al. 2017 [29]	Weng et al. 2021 [39]		
22	Shao et al. 2024 [31]	Wu 2020 [40]		
23	Tan et al. 2024 [33]	Yang et al. 2022† [43]		
24	Tu et al. 2019 [34]	Zhang et al. 2018 [44]		
25	Wang et al. 2022† [35]	Zhang et al. 2020 [46]		

26	Weng et al. 2021 [39]	Zhang et al. 2021 [47]		
27	Wang et al. 2024 [37]	Zhang et al. 2024 [47]		
28	Wang et al. 2025† [38]	Zhang et al. 2024 [48]		
29	Wu et al. 2024† [41]	Zhu 2022† [51]		
30	Yang 2020 [42]			
31	Zhang 2020† [45]			
32	Zhang 2020 [46]			
33	Zhang 2021[47]			
34	Zhang et al. 2024†§ [48]			
35	Zhang et al. 2024† [49]			
36	Zhao 2022 [50]			
37	Zhu 2022 [51]			

*Note that this study includes predictions based on features extracted by a multi-channel and multi-task deep learning model with the ability to simultaneously detect EGFR and KRAS oncogene mutations. Consequently, it did not include radiomics features (only single-task results for the independent prediction of EGFR and KRAS were considered for the quantitative analysis).

†These studies developed more than one model.

‡This study includes single- and multi-task deep neural network models according to the integration or not of a segmentation process, as well as a radiomic feature model and a feature fusion model (a hybrid multitask deep neural network radiomics model integrating both radiomic and deep features).

§This study develops a multiphase CT-based model integrating contrast and non-contrast enhanced images.

¶This study includes two different models, one based on pre-contrast images and another one on post-contrast images.

****These studies also developed models for EGFR prediction.**

††This study developed a multi-channel multi-task deep learning model for the prediction of both KRAS and EGFR mutations. However, and according to the inclusion criteria, only single-task metrics were considered for the quantitative analysis despite the multi-channel version displayed the highest performance for the simultaneous detection of both oncogenic driver mutations.

ALK, anaplastic lymphoma kinase; EGFR, epidermal growth factor receptor; KRAS, Kirsten rat sarcoma viral oncogene homologue.

Supplementary Table S6. Type of models (radiomic model/deep learning or combined [radiomic features + clinical variables and/or deep features]) developed in the studies for EGFR prediction and the radiomics/clinical features included. EGFR, epidermal growth factor receptor.

Study	Models	Radiomic features	Clinical variables
Chang et al. 2021 [1]	Radiomic	ShortRunLowGreyLevelEmphasis_AllDirection_offset1_SDH<br>Percentile85<br>OneVoxelVolume<br>Flatness<br>ShortRunEmphasis_AllDirection_offset_SD<br>HaralickCorrelation_AllDirection_offset4_SD<br>Zone Percentage<br>GLCM_Entropy_AllDirection_offset7_SD<br>Correlation_AllDirection_offset7_SD<br>CT_GLCMEntropy_AllDirection_offset1_SD<br>HaralickCorrelation_angle135_offset7	N/A

		**LongRunHighGreyLevelEmphasis_angleO_offset 1**<br>**ShortRunLowGreyLevelEmphasis_AllDirection_offset7_SD**<br>**HaralickCorrelation_AllDirection_offset1_SD**<br>**SurfaceVolumeRatio**	
**Cheng et al. 2023 [3]**	**Radiomic**	**lbp-2D_glszm_SAHGLE**<br>**lbp-3D-k_firstorder_Skewness**	**N/A**
**Dong et al. 2021 [4]**	**Deep learning**	**Not specified**	**N/A**
**Dong et al. 2022 [4]**	**Combined**	**wavelet-HLL_GLCM_MaximumProbability**<br>**wavelet-LLL_GLCM_MaximumProbability**<br>**original_GLCM_SumEntropy)**<br>**log-sigma-1-0-mm-3D_GLCM_MaximumProbability**<br>**wavelet-LHL_firstorder_Kurtosis**<br>**wavelet-LLL_firstorder_Skewness**<br>**log-sigma-2-0-mm-3D_firstorder_Kurtosis**<br>**original_shape_Sphericity**	**Smoking status**<br>**Histological type**

		wavelet-LHL_GLSZM_LargeAreaHighG	
**Feng et al. 2022 [6]**	**Radiomic**	**Skewness.7_firstorder_wavelet-LHL**<br>**SmallAreaHighGrayLevelEmphasis.7_GLSZM_wavelet-LHL**<br>**HighGrayLevelZoneEmphasis.12_GLSZM_wavelet-HHH**<br>**90Percentile_firstorder_original**<br>**Variance.4_firstorder_square**<br>**Range.4_firstorder_square**<br>**GrayLevelVariance.26_GLSZM_wavelet-LHH**<br>**JointAverage.11_GLCM_wavelet-HLH**<br>**MeanAbsolute Deviation.4_firstorder_square**<br>**RobustMeanAbsoluteDeviation.4_firstorder_square**<br>**GrayLevelNonUniformity.32_GLSZM_wavelet-LLH |**<br>**GrayLevelNonUniformity.1_girlm_original**<br>**GrayLevelNonUniformity.4_girlm_logarithm**<br>**GrayLevelNonUniformity.16_girlm_squareroot**<br>**HighGrayLevelRunEmphasis.5_girlm_squareroot**<br>**GrayLevelNonUniformityNormalized.21_GLSZM_wavelet-LLH**<br>**LowGrayLevelRunEmphasis girlm_original**	**N/A**

		**LowGrayLevelRunEmphasis.5_girlm_squareroot GrayLevel Variance.32_GLSZM_wavelet-LLH**<br>**Minimum.4_firstorder_square**<br>**SmallArealowGrayLevelEmphasis.11_GLSZM_wavelet-HLH**<br>**SmallArealowGrayLevelEmphasis.12_GLSZM_wavelet-HHH**<br>**Mean.12_firstorder_wavelet-HHH**<br>**SmallArealowGrayLevelEmphasis.9_GLSZM_wavelet-HLL**<br>**Imc2.12 GLCM wavelet-HHH**<br>**ADC**	
**Gao et al. 2023 [6]**	**Radiomic**<br>**Combined**	**original_firstorder_Kurtosis**<br>**original_firstorder_Median**<br>**original_firstorder_Skewness**<br>**log-sigma-1-0-mm-3D_firstorder_Energy**<br>**log-sigma-4-0-mm-3D_GLDM_DependenceVariance**<br>**wavelet-LHL_GLRLM_LongRunLowGrayLevelEmphasis**<br>**wavelet-HLL_firstorder_Energy**	**CEA**<br>**Sex (male)**<br>**Nodule type (sub-solidity)**

**Gong et al. 2023 [8]**	**Radiomic**<br>**Deep learning**<br>**Combined (radiomics + deep features)**	**RADIOMIC MODEL:**<br>**Log-sigma-2.0-mm-3D_glcm_Idmn**<br>**Log-sigma-2.0-mm-3D_glcm_Id**<br>**Log-sigma-2.0-mm-3D_glcm_Idn**<br>**Wavelet_LLL_ngtdm_Contrast**<br>**Wavelet_LLL_ngtdm_Strength**<br>**COMBINED MODEL:**<br>**Not specified**	**N/A**
**Hinzpeter et al. 2024 [9]**	**Radiomic**	**MOST IMPORTANT FEATURES:**<br>**log GLZLM LZLGE**<br>**GLCM Energy AngularSecondMoment**<br>**GLCM Correlation**<br>**log GLCM Contrast Variance**<br>**CONVENTIONAL HUmax**	**N/A**

**Hu et al. 2024 [10]**	**Radiomic**	**Square_ngtdm_Complexity**<br><br>**Wavelet-LLL_glcm_Idn**<br><br>**Log-sigma-1-0-mm-3D_glrlm_LongRunHighGray LevelEmphasis**<br><br>**Lbp-3D-k_firstorder_10Percentile**<br><br>**Logarithm_gldm_DependenceEntropy**<br><br>**Exponential_gldm_DependenceNonUniformity Normalized**	**N/A**
**Huo et al. 2022* [11]**	**Combined**	**137 features (not specified)**	**Age**<br><br>**Sex (female)**<br><br>**Non-smokers**<br><br>**Clinical stage (I-II)**
**Jia et al. 2019 [12]**	**Combined**	**94 features (not specified)**	**Sex**<br><br>**Smoking history**
**Jiang et al. 2022 [13]**	**Combined**	**Skewness**<br><br>**Minimum**	

		**Kurtosis**<br>**Variance**<br>**Minimum**<br>**10th percentile**<br>**SumSquare**<br>**SizeZoneNonUniformity**<br>**HighGrayLevelZoneEmphasis**<br>**ZoneVariance**<br>**LargeDependence HighGrayLevelEmphasis**<br>**LargeDependenceHighGrayLevel Emphasis**<br>**DependenceEntropy**	**Age**<br>**Sex**<br>**Smoking**<br>**Tumor**<br>**Family history**
**Kohan et al. 2024 [14]**	**Radiomic**<br>**Combined**	**PARAMS ZSpatialResampling**<br>**log SHAPE Volume vx**<br>**log NGLDM Coarseness**<br>**CONVENTIONAL HUpeakSphere0 5mL discretized**	**Age**<br>**Sex**

		volume sought<br>GLZLM ZP	Smoking status<br>Race (White vs Asian)
Le et al. 2021 [15]	Radiomic	wavelet-LLLfirstorderEnergy<br>wavelet-LHHGLSZMGrayLevelNonUniformityNormalized<br>wavelet-HHLGLDMSmallDependenceLowGratLevelEmphasis<br>wavelet-HLHGLCM_MCC<br>wavelet-HLHGLSZMSmallAreaLowGrayLevelEmphasis<br>wavelet-HHHGLCMjointEnergy<br>wavelet-HHHGLRLMGrayLevelNonUniformityNormalized	N/A
Li et al. 2018[16]	Radiomic<br>Combined	338 features (not specified)	Sex<br>Smoking status
Li et al. 2019 [17]	Radiomic<br>Combined	CT_GGS_Gray Span<br>CT_GGC_Gray Mean	Age<br>Sex<br>Smoking status<br>Clinical stage

			Lesion location
Li et al. 2020 [18]	Combined	12 features (not specified)	Sex<br>Age<br>Smoking status
Li et al. 2022 [19]	Radiomic	3 features (not specified)	–
Liu et al. 2020 Liu, 2020 #20}	Radiomic<br>Combined	RADIOMIC MODEL:<br>wavelet-HLH_GLDM_DependenceVariance<br>wavelet-LHL_GLDM_LargeDependenceLowGrayLevelEmphasis<br>logarithm_GLCM_InverseVariance<br>square_GLDM_DependenceVariance<br>wavelet-HLH_GLDM_LargeDependenceHighGrayLevelEmphasis<br>wavelet-HHH_GLCM_Id<br>log-sigma-0-5-mm-3D_GLSZM_ZoneEntropy<br>square_GLCM_Correlation<br>original_GLCM_ClusterShade	Age<br>Sex

		**wavelet-LHH_GLDM_LargeDependenceHighGrayLevelEmphasis**<br><br>**COMBINED MODEL:**<br>**wavelet-HLH_GLDM_DependenceVariance**<br>**custom_PatientSex**<br>**logarithm_GLCM_InverseVariance**<br>**square_GLCM_Correlation**<br>**wavelet-HLL_firstorder_Kurtosis**<br>**wavelet-LHL_GLRLM_LongRunLowGrayLevelEmphasis**<br>**wavelet-HLL_firstorder_Median**<br>**original_GLSZM_SizeZoneNonUniformityNormalized**<br>**exponential_firstorder_Skewness**<br>**wavelet-LLH_GLCM_ClusterShade**	**Smoking history**
**Liu et al. 2022 [21]**	**Radiomic**	**Mean absolute deviation**<br>**60 Percentile area**	

		Convex<br>Correlation<br>Dissimilarity<br>5-1 Homogeneity 2<br>10-4 Homogeneity 2<br>-333-7 Information measure corr 1<br>8-1 Information measure corr 1<br>9-7 Information measure corr 1<br>2-4 Inverse diff norm<br>6-4 Inverse variance<br>8-4 Inverse variance<br>8-1 Max Probability<br>12-7 Max Probability<br>-333 Run length nonuniformity	–

Lu et al. 2020 [22]	Radiomic<br>Combined	original_GLSZM_SmallAreaHighGrayLevelEmphasis<br>original_GLSZM_SmallAreaLowGrayLevelEmphasis<br>original_GLDM_LowGrayLevelEmphasis<br>log-sigma-1-0-mm-3D_GLCM_Cluster Prominence<br>log-sigma-3-0-mm-3D_GLDM_DependenceNonUniformityNormalized wavelet-LLL_GLCM_InverseVariance<br>wavelet-LLH_GLCM_Imc2<br>wavelet-HLL_firstorder_Mean<br>wavelet-HLL_GLSZM_LowGrayLevelZoneEmphasis<br>wavelet-HLL_GLDM_SmallDependenceHighGrayLevelEmphasis<br>wavelet-HLH_GLSZM_SizeZoneNonUniformityNormalized<br>wavelet-HHH_firstorder_Skewness<br>wavelet-HHH_GLSZM_Size Zone Non Uniformity Normalized	Sex<br>Smoking status Pathohistological subtype<br>Vascular infiltration status
Lu et al. 2022 [23]	Radiomic<br>Combined	1269 features (not specified)	Age<br>Sex<br>Smoking status

			**Stage of disease Serum level of tumor markers (CEA, CYFRA 21-1, SCC, Pro-GRP)**
**Ma et al. 2024 [25]**	**Radiomic**<br>**Combined (radiomic features + clinical data)**	**Five radiomics features: one first-order feature, two GLCM features, one GLRLM feature, and one GLSZM feature.**	**Smoking history**<br>**Sex**<br>**Age**
**Nair et al. 2021† [26]**	**Radiomic**	**NGTDM_600_Complexity**<br>**Glrl_Saggital_30_ShortRunEmphasis**<br>**Glrl_Saggital_30_ShortRunHighGrayLevelEmphasis**<br>**Glrl_Saggital_120_ShortRunHighGrayLevelEmphasis**<br>**Glrl_Coronal_120_ShortRunHighGrayLevelEmphasis**<br>**Glrl_Coronal_30_ShortRunEmphasis**<br>**Glrl_Saggital_120_ShortRunEmphasis**	**–**

		**Glrl_Axial_30_ShortRunEmphasis**<br><br>**Glrl_Coronal_120_ShortRunEmphasis**<br><br>**FirstOrder_HistogramBin2**	
**Ninomiya et al. 2021 [27]**	**Radiomic**	**BN MODEL:**<br><br>**b0_GLCM_Energy_45,**<br><br>**b1/b0_GLSZM_ZSN_104**<br><br>**b1_GLCM_SumAverage_122**<br><br>**b0_GLRLM_Lrlge_97)**<br><br><br>**OI MODEL:**<br><br>**GLRLM_ ShortRunLowGrayLevelEmphasis**<br><br>**GLSZM_ LowGrayLevelZoneEmphasis**<br><br>**GLSZM_ShortZoneLowGrayEmphasis**<br><br><br>**WD MODEL:**	**–**

		**GLSZM_ LowGrayLevelZoneEmphasis _LL**	
**Ninomiya et al. 2023 [28]**	**Combined**	**GLSZM_SmallAreaLowGrayLevelEmphasis**<br>**GLSZM_LargeAreaEmphasis**<br>**Hist.RootMeanSquared**<br>**GLDM_DependenceVariance**	**Sex**<br>**Smoking status**
**Rios Velazquez et al. 2017 [29]**	**Radiomic**<br>**Combined**	**imaging.Wavelet_LHH_GLCM_invDiffmomnor**<br>**imaging.LoG_sigma_3_mm_3D_GLSZM_highIntensityLarteAreaEmp**<br>**imaging.Wavelet_LLL_GLCM_clusProm**<br>**imaging.GLCM_maxProb**<br>**imaging.Wavelet_LLL_stats_energy imaging.Wavelet_HLL_stats_var imaging.LoG_sigma_3_mm_3D_GLSZM_largeAreaEmphasis imaging.Wavelet_LLH_stats_range imaging.Wavelet_LHH_GLCM_clusProm imaging.Wavelet_LLL_GLSZM_highIntensityLarteAreaEmp imaging.Wavelet_HLH_GLSZM_lowIntensitySmallAreaEmp imaging.Wavelet_HHL_stats_energy imaging.Wavelet_LLH_stats_mean**<br>**imaging.Stats_median imaging.Wavelet_HHL_GLCM_maxProb imaging.LoG_sigma_3_mm_3D_GLCM_clusProm imaging.Shape_spherDisprop**	**Stage**<br>**Sex**<br>**Smoking status**<br>**Age**<br>**Race**

		imaging.Stats_kurtosis<br><br>imaging.Wavelet_HHH_GLCM_correl1<br>imaging.LoG_sigma_3_mm_3D_rlgl_grayLevelNonuniformity	
Rossi et al. 2021 [30]	Combined	First order_90 Percentile<br><br>First order_Entropy<br><br>First order_Maximum<br><br>First order_Median<br><br>First order_Robust mean absolute deviation<br><br>First order_Root mean squared<br><br>First order_Skewness<br><br>First order_Uniformity<br><br>GLCM_Correlation<br><br>GLCM_Difference average<br><br>GLCM_Difference entropy<br><br>GLCM_InverseDifference<br><br>GLCM_ InverseDifferenceMoment	Sex<br><br>Smoking status

		**GLCM_ InverseDifferenceMomentNormalized**<br><br>**GLCM_ InverseDifferenceNormalized**<br><br>**GLCM_ InformationalMeasureCorrelation1**<br><br>**GLCM_ InformationalMeasureCorrelation2**<br><br>**GLCM_InverseVariance**<br><br>**GLCM_JointEnergy**<br><br>**GLCM_JointEntropy**<br><br>**GLCM_MaximalCorrelationCoefficient**<br><br>**GLCM_MaximumProbability**<br><br>**GLCM_SumEntropy**<br><br>**GLDM_DependenceEntropy**<br><br>**GLDM_DependenceNonUniformity**<br><br>**GLDM_Dependence NonUniformityNormalized**<br><br>**GLDM_DependenceVariance**<br><br>**GLDM_GrayLevelNonUniformity**	

		**GLDM_LargeDependenceEmphasis**<br>**GLDM_SmallDependenceEmphasis**<br>**GLRLM_GrayLevelNonUniformity**<br>**GLRLM_GrayLevelNonUniformityNormalized**<br>**GLRLM_RunEntropy**<br>**GLRLM_RunPercentage**<br>**GLRLM_ShortRunEmphasis**<br>**GLSZM_GrayLevelNonUniformity**<br>**GLSZM_GrayLevelNonUniformityNormalized**<br>**GLSZM_SizeZoneNonUniformityNormalized**<br>**GLSZM_SmallAreaEmphasis**<br>**GLSZM_ZoneEntropy**<br>**GLSZM_ZoneVariance**<br>**NGTDM_Coarseness**	
**Shao et al. 2024 [31]**	**Deep learning**	**Not specified**	**N/A**

**Tan et al. 2024 [33]**	**Radiomic**	**SphericalDisproportion**<br>**IntensityHistogramSkewness**<br>**IntensityHistogramMinimumGreyLevel**<br>**IntensityHistogramInterquartileRange**<br>**GLCM_InverseDifferenceMoment**<br>**GLRLM_LongRunsEmphasis**<br>**GLSZM_SmallZoneHighGreyLevelEmphasis**	**N/A**
**Tu et al. 2019 [34]**	**Radiomic**<br>**Combined**	**X0_GLRLM_ RunLengthNon-Uniformity**<br>**X4_H_median**<br>**X0_GLCM_homogeneity1**	**Maximum diameter**<br>**Location**<br>**Sex**
**Wang et al. 2022 [35]**	**Radiomic**<br>**Combined‡**	**Not specified**	**Age**<br>**Sex**<br>**Tumor staging**<br>**Number**

			**Size**<br>**Past recurrence**<br>**Medication status**
**Wang et al. 2025 [38]**	**Radiomic**<br>**Deep Learning**<br>**Combined (Deep learning + radiomics; Deep learning + clinical data; Deep learning + radiomics + clinical data)**	**Wavelet_LHH_glrlm_LongRunLowGrayLevelEmphasis**<br>**Log_sigma_5_0_mm_3D_firstoder_10Percentile**<br>**Wavelet_LHL_firstorder_Skewness**<br>**Wavelet_HHH_glcm_MaximumProbability**<br>**Wavelet_HLH_glszm_GrayLevelNonUniformityNormalized**<br>**Wavelet_HLL_glcm_MCC**<br>**Wavelet_HHH_glszm_ZoneVariance**<br>**Wavelet_HHL_glcm_MCC**<br>**Log_sigma_3_0_mm_3D_glcm_InverseVariance**	**Sex**<br>**Age**<br>**Smoking status**

Wang et al. 2024 [37]	Radiomic	12 features (not specified)	N/A
Weng et al. 2021 [39]	Radiomic<br>Combined	SmallAreaEmphasis<br>LongRunHigh GreyLevelEmphasis_angle0_offset4<br>ClusterProminence_All Direction_offset7_SD<br>InverseDifference Moment_All Direction_offset4_SD<br>LowGreyLevel Run Emphasis_All Direction_offset4_SD<br>LongRunLowGrey Level Emphasis_All Direction_offset7_SDCorrelation_angle0_offset7<br>std Deviation<br>GLCM Energy_All Direction_offset4_SD	Smoking status<br>Spiculation<br>Air bronchogram<br>CEA<br>SCCA
Wu 2020 [40]	Combined	Not specified	Smoking status<br>Histological subtype
Wu et al. 2024 [41]	Deep learning	Not specified	

**Yang 2020 [42]**	**Radiomic**	**Not specified**	**–**
**Yang 2022 [43]**	**Radiomic[§]**<br>**Combined**	**Nonwavelet-LHH_NGTDM_Strength**<br>**wavelet-LHH_GLDM_DependenceEntropy**<br>**wavelet-LLL_GLSZM_LargeAreaLowGrayLevelEmphasis**<br>**wavelet-LLL_firstorder_Minimum**<br>**wavelet-LLH_NGTDM_Contrast**<br>**wavelet-LHH_NGTDM_Strength**<br>**log-sigma-1-5-mm-3D_firstorder_Kurtosis**<br>**wavelet-LHL_GLCM_ClusterShade**<br>**wavelet-LHH_NGTDM_Strength**<br>**wavelet-LLL_GLSZM_LargeAreaLowGrayLevelEmphasis**<br>**wavelet-LLH_firstorder_Mean**<br>**original_NGTDM_Contrast**<br>**original_firstorder_Kurtosis**<br>**log-sigma-1-5-mm-3D_firstorder_Kurtosis**	**Sex**<br>**Emphysema**<br>**Interstitial lung disease**

		**wavelet-LLL_NGTDM_Contrast**<br>**original_GLCM_MaximumProbability**<br>**wavelet-LLL_GLSZM_LargeAreaLowGrayLevelEmphasis**	
**Zhang 2018 [44]**	**Combined**	**IIF.range**<br>**IIF.Skewness**<br>**W_{LLH}F.IF.mean_absulute_eviation**<br>**W_{LHH}F.IF.median**<br>**W_{LLH}F.IF.mean**<br>**W_{LLH}F.GLCM.variance**<br>**GLRLM_ HighGrayLevelRunEmphasis**	**Histological subtype**<br>**Sex**<br>**Smoking status**
**Zhang 2020 [45]**	**Radiomic**	**GLSZM_ HighGrayLevelZoneEmphasis**<br>**GLDM_DependenceVariance**<br>**GLSZM_ GreyLevelNon UniformityNormalized**<br>**GLSZM_ZoneEntropy**	**–**
**Zhang 2020 [46]**	**Radiomic**	**784 features (not specified)**	**Sex**

	**Combined**		**Histopathological Subtype**<br>**Age**
**Zhang 2021 [47]**	**Radiomic**<br>**Combined**	**fo_Skewness**<br>**exp_GLRLM_ShortRunEmphasis**<br>**exp_GLRLM_ShortRunHighGrayLevelEmphasis**<br>**exp_GLDM_SmallDependenceEmphasis**<br>**grad_GLDM_DependenceEntropy**<br>**LLH_fo_90P**<br>**LLH_GLCM_SumEntropy**<br>**LLL–fo_kurtosis**<br>**LLL–GLCM_ClusterProminence**<br>**LLL_GLSZM_GrayLevelNonUniformityNormalized**<br>**LLL_GLSZM_GrayLevelVariance**<br>**LLL_GLSZM_ZoneEntropy**	**Smoking history**<br>**Bubble-like lucency**<br>**Pleural attachment**<br>**Pleural retraction**

Zhang et al. 2024 [48]	Radiomic<br>Combined (radiomic features + clinical data	**RADIOMIC MODEL (NON-ENHANCED CT):**<br>GLRLM_Long Run High Gray Level Emphasis<br>Median<br>NGTDM_Busyness<br>GLDM_Dependence Entropy<br>**COMBINED MODEL:**<br>GLRLM_Long Run High Gray Level Emphasis<br>Median<br>NGTDM_Busyness<br>GLDM_Dependence Entropy<br>NGTDM_Strength<br>GLRLM_Short Run Emphasis<br>GLCM_Sun Entropy<br>First-order_90th Percentile	Age<br>Sex<br>Long-axis diameter<br>Short-axis diameter
Zhang et al. 2024 [49]	Radiomic	**RADIOMIC MODEL:**	N/A

	**Deep learning**<br>**Combined (radiomic + deep features)**	**Gradient_LargeDependenceHighgraylevelEmphasis**<br>**Square_glszm_GrayLevelVariance**<br>**Gradient_glrlm_LongRunLowGrayLevelEmphasis**<br>**Square_glszm_SizeZoneNonUniformityNormalized**<br>**Gradient_glrlm_RunVariance**<br>**Gradient_glcm_ClusterProminence**<br>**Original_glszm_SmallAreaEmphasis**<br>**Exponential_glszm_LargeAreEmphasis**<br>**Exponential_firstorder_TotalEnergy**<br>**Exponential_glrlm_ShortRunLowGrayLevelEmphasis**<br>**Exponential_gldm_LargeDependenceHighGrayLevelEmphasis**<br>**Original_glcm_Maximumprobability**<br>**Squareroot_firstorder_Kurtosis**	
**Zhao 2022§ [50]**	**Radiomic**	**CT_Shape_Sphericity**	**–**

		CT_GLRLM_ShortRunEmphasis<br>CT_GLRLM_ShortRunHighGreyLevelEmphasis<br>CT_NGLDM_Busyness<br>CT_GlzIm_ShortZoneEmphasis	
Zhu 2022 [51]	Radiomic<br>Combined	log_sigma_1.0_mm_3D_GLRLM_RunVariance<br>wavelet_LLH_firstorder_RootMeanSquared<br>log-sigma-2-0-mm-3D_GLCM_ClusterShade<br>wavelet_HHH_firstorder_Mean	Sex<br>Age<br>Emphysema<br>Pathological subtype

*Combined model also included 14 CT features: location (peripheral), tumor size ≥3cm, subsolid density, spiculation, lobulation, air bronchogram, air space, necrosis, calcification (presence), vascular convergence sign, pleural retraction sign, pleural effusion, lymphatic metastasis and multiple pulmonary metastasis.

†Top 10 selected features. The maximum number of texture features included was determined by maximizing cross-validated accuracy. This value was not the same for each binary group or each machine learning model.

‡Note that this model includes radiomic features + deep features and clinical variables.

§Sensitivity and specificity (or confusion matrices to calculate them) were not available for radiomic models

#Model 1.

BN, Betti numbers; CEA, carcinoembryonic antigen; CYFRA 21-1, fragment of cytokeratin sub-unit 19; GLCM, gray-level co-occurrence matrix; GLDM, gray-level dependence matrix; GLRLM, gray-level run-length matrix; GLSZM, gray-level size zone matrix; NGTDM, neighboring gray tone difference matrix; OI, original image; Pro-GRP, pro-gastrin-releasing peptide; SCC, squamous cell carcinoma antigen; WD, wavelet decomposition.

Supplementary Table S7. Type of models (radiomic model or combined [radiomic features + clinical variables]) developed in the studies for ALK prediction and the radiomics/clinical features included. ALK, anaplastic lymphoma kinase.

Study	Models	Radiomic features	Clinical variables
Chang et al. 2021 [2]	Radiomic	CT_uniformity<br>CT_LongRunEmphasis_AllDirection_offset4_SD<br>CT_HaraEntropy<br>CT_GLCMEnergy_angle135_offset7<br>CT_LongRunHighGreyLevelEmphasis_angle45_offset1<br>CT_LongRunLowGreyLevelEmphasis_AllDirection_offset7_SD<br>CT_Correlation_AllDirection_offset4_SD<br>CT_Percentile70<br>CT_HaralickCorreltion_AllDirection_offset4_SD<br>CT_LongRunLowGreyLevelEmphasis_AllDirection_offset4_SD<br>CT_LongRunEmphasis_angle135_offset4	–

		CT_LongRunHighGreyLevelEmphasis_angle90_offset4<br><br>CT_LongRunLowGreyLevelEmphasis_AllDirection_offset1_SD<br><br>CT_HaralickCorreltion_AllDirection_offset7_SD<br><br>CT_ShortRunEmphasis_AllDirection_offset1_SD<br><br>CT_LongRunHighGreyLevelEmphasis_angle0_offset1<br><br>CT_GLCMEntropy_angle90_offset1<br><br>CT_Percentile30<br><br>CT_LongRunEmphasis_angle90_offset4<br><br>CT_LongRunEmphasis_AllDirection_offset1_SD	
**Ma et al. 2020 [24]**	**Radiomic**	**PRE-CONTRAST MODEL:**<br><br>**wavelet-LLL_GLCM_DifferenceVariance**<br><br>**wavelet-LLH_firstorder_Median**<br><br>**wavelet-LLH_NGTDM_Busyness**<br><br>**wavelet-LHL_GLSZM_LargeAreaLowGrayLevelEmphasis**	**–**

		**wavelet-HHH_GLSZM_LargeAreaLowGrayLevelEmphasis**<br>**wavelet-LHL_firstorder_Energy**<br>**wavelet-HHL_firstorder_90Percentile**<br>**wavelet-HHL_GLCM_JointEntropy**<br>**wavelet-HHL_firstorder_Uniformity**<br>**wavelet-HHL_firstorder_RobustMeanAbsoluteDeviation**<br>**wavelet-LHH_GLDM_LargeDependenceLowGrayLevelEmphasis**<br>**wavelet-HLH_firstorder_Median**<br>**wavelet-LHL_GLDM_LargeDependenceLowGrayLevelEmphasis**<br>**wavelet-HHL_GLCM_InverseDifference**<br>**wavelet-HHL_firstorder_InterquartileRange**<br>**wavelet-HHL_GLCM_MaximumProbabiblity**<br>**wavelet-HHH_GLSZM_SmallAreaLowGrayLevelEmphasis**<br>**wavelet-HHL_firstorder_Mean**<br>**wavelet-HLL_GLCM_ClusterShade**	

		**wavelet-HHL_GLSZM_SmallAreaLowGrayLevelEmphasis**<br><br>**wavelet-LHH_GLCM_ MaximalCorrelationCoefficient**<br><br>**wavelet-LLL_GLSZM_SizeZoneNonUniformityNormalized**<br><br>**wavelet-LLL_GLSZM_SmallAreaEmphasis**<br><br>**wavelet-HHL_GLCM_ InverseDifferenceNormalized**<br><br><br>**POST-CONTRAST MODEL:**<br><br>**wavelet-LHH_GLDM_SmallDependenceHighGrayLevelEmphasis**<br><br>**wavelet_HHL_GLSZM_GrayLevelNonUniformity**<br><br>**wavelet-LLH_firstorder_Mean**<br><br>**wavelet-LLH_GLSZM_HighGrayLevelZoneEmphasis**<br><br>**wavelet-LLH_GLSZM_SmallAreaHighGrayLevelEmphasis**<br><br>**wavelet-LLH_GLSZM_SmallAreaLowGrayLevelEmphasis**<br><br>**wavelet-HHH_GLCM_MaximumProbability**<br><br>**wavelet-LLL_GLDM_LargeDependenceLowGrayLevelEmphasis**	

		**wavelet-HLL_GLDM_DependenceVariance**<br><br>**wavelet-HHH_firstorder_Mean**<br><br>**wavelet-HHH_GLDM_LowGrayLevelEmphasis**<br><br>**wavelet-LLH_firstorder_90Percentile**<br><br>**wavelet-HHL_GLDM_DependenceVariance**<br><br>**wavelet-HHH_GLCM_MaximalCorrelationCoefficient**<br><br>**wavelet-HHH_NGTDM_Contrast**<br><br>**wavelet-original_GLCM_InverseVariance**<br><br>**wavelet-LLH_firstorder_Range**<br><br>**wavelet-HHL_GLCM_MaximalCorrelationCoefficient**<br><br>**wavelet-HLL_GLSZM_GrayLevelNonUniformityNormalized**	
**Song et al. 2020 [32]**	**Radiomic**<br><br>**Combined***	**RADIOMIC MODEL:**<br><br>**Original_Firstorder_90Percentile**<br><br>**Original_Firstorder_Entropy**	

		**Original_Firstorder_Maximum**<br>**Wavelet-LHH_Firstorder_10Percentile**<br>**Wavelet-HLL_Firstorder_Median**<br>**Wavelet-HHH_Firstorder_Mean**<br>**LoG-sigma-1-0-mm-3D_Firstorder_Median**<br>**LoG-sigma-1-0-mm-3D_Firstorder_RootMeanSquared**<br>**LoG-sigma-1-0-mm-3D_Firstorder_Minimum**<br>**LoG-sigma-2-0-mm-3D_Firstorder_10Percentile**<br>**LoG-sigma-3-0-mm-3D_Firstorder_90Percentile**<br>**LoG-sigma-5-0-mm-3D_Firstorder_Skewness**<br>**Original_ GLCM _ClusterShade**<br>**Wavelet-LHH_ GLCM _Correlation**<br>**Wavelet-LHL_ GLCM _InverseDifferenceNormalized**<br>**Wavelet-HHH_ GLCM _InformationalMeasureofCorrelation1**<br>**LoG-sigma-1-0-mm-3D_GLCM _Autocorrelation**	**Age**<br>**Sex**<br>**Smoking history**<br>**Smoking index**<br>**Clinical stage**<br>**Distal metastasis**<br>**Pathological invasiveness of the tumor**

		**LoG-sigma-2-0-mm-3D_GLCM _InverseVariance**<br><br>**Original_GLSZM_ SmallAreaHighGrayLevelEmphasis**<br><br>**Wavelet-HHH_ GLSZM _ SmallAreaHighGrayLevelEmphasis**<br><br>**Wavelet-HLL_ GLSZM _ZoneEntropy**<br><br>**Wavelet-HLH_ GLSZM _ZoneEntropy**<br><br>**LoG-sigma-2-0-mm-3D_GLSZM _ZoneEntropy**<br><br>**LoG-sigma-3-0-mm-3D_GLSZM _ SmallAreaEmphasis**<br><br>**LoG-sigma-3-0-mm-3D_GLSZM _ Size-ZoneNonUniformityNormalized**<br><br>**LoG-sigma-5-0-mm-3D_GLSZM _ GrayLevelNonUniformityNormalized**<br><br>**Wavelet-LHH_GLDM_ LargeDependenceHighGrayLevelEmphasis**<br><br>**LoG-sigma-1-0-mm-3D_GLDM _ HighGrayLevelEmphasis**<br><br>**LoG-sigma-3-0-mm-3D_GLRLM_RunPercentage**<br><br>**LoG-sigma-4-0-mm-3D_GLRLM _ LongRunLowGrayLevelEmphasis**<br><br><br>**COMBINED MODEL:**	

		**Current smoker**<br>**Stage I**<br>**Male**<br>**Local lymphadenopathy**<br>**Pericardial effusion**<br>**Left Lower Lobe lesion**<br>**No cavity in the lesion**<br>**Lobulated margin**<br>**No pleural retraction sign**<br>**No local lymphadenopathy**<br>**Wavelet-HHL_Firstorder_Kurtosis**<br>**Wavelet-HLL_Firstorder_Median**<br>**Wavelet-LHH_Firstorder_Skewness**<br>**Wavelet-LLL_Firstorder_Minimum**<br>**Wavelet-HLH_Firstorder_Median**	

		**LoG-sigma-1-0-mm-3D_Firstorder_Minimum**<br><br>**LoG-sigma-2-0-mm-3D_Firstorder_Minimum**<br><br>**Wavelet-LLL_GLCM_ClusterShade**<br><br>**Wavelet-LLH_GLCM _InformationalMeasureofCorrelation2**<br><br>**Wavelet-HLH_GLCM _InformationalMeasureofCorrelation2**<br><br>**Wavelet-HLH_GLCM_InformationalMeasureofCorrelation1**<br><br>**LoG-sigma-1-0-mm-3D_GLCM_InformationalMeasureofCorrelation1**<br><br>**LoG-sigma-3-0-mm-3D_GLCM_InformationalMeasureofCorrelation2**<br><br>**LoG-sigma-5-0-mm-3D_GLCM_InformationalMeasureofCorrelation2**<br><br>**Original_Shape_MajorAxisLength**<br><br>**Wavelet-HLH_GLSZM_SizeZoneNon-Uniformity**<br><br>**LoG-sigma-4-0-mm-3D_GLSZM_GrayLevelNonUniformityNormalized**<br><br>**Wavelet-HLH_GLDM_ LargeDependenceHigh GrayLevelEmphasis**<br><br>**Wavelet-HHH_GLDM_ LargeDependenceHigh GrayLevelEmphasis**<br><br>**Original_GLRLM_ HighGrayLevelRunEmphasis**	

***Not included in the meta-analysis due to the lack of enough studies to run the analysis.**

GLCM, gray-level co-occurrence matrix; GLDM, gray-level dependence matrix; GLRLM, gray-level run-length matrix; GLSZM, gray-level size zone matrix; NGTDM, neighboring gray tone difference matrix.

Supplementary Table S8. Type of models (radiomic model or combined [radiomic features + clinical variables]) developed in the studies for KRAS prediction and the radiomics/clinical features included. KRAS, Kirsten rat sarcoma viral oncogene homologue.

Study	Models	Radiomic features	Clinical variables
Dong et al. 2021 [4]	Radiomic	Not specified	–
Hinzpeter et al. 2024 [9]	Radiomic	MOST IMPORTANT FEATURES:<br>GLCM Correlation<br>GLRLM HGRE<br>log CONVENTIONAL HUcalciumAgatstonScore<br>DISCRETIZED HISTO Energy Uniformity<br>log NGLDM Contrast	N/A
Kohan et al. 2024 [14]	Radiomic	PARAMS ZSpatialResampling<br>log CONVENTIONAL HUSkewness	N/A

	Combined (radiomic features + clinical data)*	Log NGLDM Contrast<br>GLCM Correlation<br>onlyForCT	
Le et al. 2021 [15]	Radiomic	wavelet-LLHGLSZMLargeAreaEmphasis<br>wavelet-LLLGLDMDependenceEntropy<br>wavelet-LHHGLDMLargeDependenceLowGrayLevelEmphasis<br>ori-firstorderkurtosis<br>wavelet-HLHGLCMInverseVariance<br>wavelet-HLLGLSZMSmallAreaHighGrayLevelEmphasis<br>wavelet-LHHGLCMId<br>wavelet-HHLGLCMDifferenceEntropy<br>wavelet-LLLGLSZMGrayLevelNonUniformityNormalized<br>wavelet-HHHGLCMDifferenceAverage<br>wavelet-HHHGLDMDependenceEntropy	–

Rios Velazquez et al. 2017 [29]	Radiomic<br>Combined	imaging.LoG_sigma_3_mm_3D_GLSZM_highIntensityLarteAreaEmp<br>imaging.Wavelet_LHH_GLCM_clusProm<br>imaging.Wavelet_LHH_GLCM_energy imaging.Wavelet_LLL_stats_energy<br>imaging.Wavelet_LLL_stats_median<br>imaging.LoG_sigma_3_mm_3D_GLSZM_largeAreaEmphasis<br>imaging.Wavelet_HHH_GLSZM_lowIntensitySmallAreaEmp<br>imaging.Wavelet_HHH_GLCM_correl1<br>imaging.LoG_sigma_3_mm_3D_GLCM_clusProm<br>imaging.Wavelet_LLL_GLSZM_highIntensityLarteAreaEmp<br>imaging.Wavelet_HHL_stats_energy imaging.Wavelet_HLL_stats_var<br>imaging.Wavelet_HLH_GLSZM_lowIntensitySmallAreaEmp<br>imaging.Wavelet_LHH_rlgl_GrayLevelNonuniformity<br>imaging.LoG_sigma_3_mm_3D_GLSZM_lowIntensitySmallAreaEmp<br>imaging.GLCM_clusShade imaging.Wavelet_LHH_GLCM_invDiffmomnor<br>imaging.Wavelet_HLL_stats_min<br>imaging.Wavelet_LLL_rlgl_longRunHighGrayLevEmpha<br>imaging.Wavelet_LLH_stats_mean	Stage<br>Sex<br>Smoking status<br>Age<br>Race
Wang et al. 2022 [36]	Radiomic	CT_square_GLSZM_SizeZoneNonUniformityNormalized<br>CT_wavelet-LHH_GLDM_DependenceNonUniformityNormalized<br>CT_wavelet-HHL_firstorder_Skewness	–

		**CT_wavelet-HHL_GLDM_DependenceNonUniformityNormalized**	

***Not included in the meta-analysis due to the lack of enough studies to run the analysis.**

GLCM, gray-level co-occurrence matrix; GLDM, gray-level dependence matrix; GLRLM, gray-level run-length matrix; GLSZM , gray-level size zone matrix.

Supplementary Table S9. Results of the meta-regression analyzing the effects of age, type of segmentation (manual/semi-automatic/automatic), type of model (radiomics/combined [radiomic features + clinical data) and artificial intelligence methodology (machine learning/deep learning).

**AGE**<br><br>**Fixed-effects coefficients**					
	**Estimate**	**SE**	**z**	***p*-value**	**CI 95%**
**tsens.(Intercept)**	**3.736**	**1.872**	**1.996**	**0.046**	**[0.068, 7.404]**
**tsens.AGE**	**-0.038**	**0.030**	**-1.264**	**0.206**	**[-0.098, 0.021]**
**tfpr.(Intercept)**	**-1.429**	**1.715**	**-0.833**	**0.405**	**[-4.792, 1.933]**
**tfpr.AGE**	**0.008**	**0.027**	**0.301**	**0.763**	**[-0.046, 0.063]**
**Variance components: between-studies Std. Dev and correlation matrix**					
	**SD**		**tsens**		**tfpr**
**tsens**	**0.478**		**–**		**0.272**
**tfpr**	**0.425**		**0.272**		**–**
**TYPE OF SEGMENTATION**<br><br>**Fixed-effects coefficients**					
	**Estimate**	**SE**	**z**	***p*-value**	**CI 95%**
**tsens.(Intercept)**	**1.371**	**0.111**	**12.319**	**0.000**	**[1.153, 1.589]**

**tsens.SegmentationSemiautomatic**	**-0.152**	**0.226**	**-0.671**	**0.502**	**[-0.595, 0.292]**
**tsens.SegmentationUnknown**	**-0.134**	**0.377**	**-0.356**	**0.722**	**[-0.873, 0.604]**
**tfpr.(Intercept)**	**-0.995**	**0.108**	**-9.196**	**0.000**	**[-1.207, -0.783]**
**tfpr.SegmentationSemiautomatic**	**0.213**	**0.216**	**0.989**	**0.323**	**[-0.209, 0.635]**
**tfpr.SegmentationUnknown**	**-0.189**	**0.369**	**-0.513**	**0.608**	**[-0.912, 0.533]**
**Variance components: between-studies Std. Dev and correlation matrix**					
	**SD**		**tsens**		**tfpr**
**tsens**	**0.499**		**–**		**0.405**
**tfpr**	**0.495**		**0.405**		**–**
**CONTRAST**<br>**Fixed-effects coefficients**					
	**Estimate**	**SE**	**z**	***p*-value**	**CI 95%**
**tsens.(Intercept)**	**1.833**	**0.462**	**3.969**	**0.000**	**[0.928, 2.738]**
**tsens.Contrastcontrast-enhanced**	**-0.374**	**0.504**	**-0.742**	**0.458**	**[-1.363, 0.614]**
**tsens.Contrastnon-contrast CT**	**-0.615**	**0.475**	**-1.295**	**0.195**	**[-1.547, 0.316]**
**tfpr.(Intercept)**	**-0.101**	**0.570**	**-0.177**	**0.860**	**[-1.217, 1.016]**
**tfpr.Contrastcontrast-enhanced**	**-1.017**	**0.424**	**-2.396**	**0.017**	**[-1.848, -0.185]**

**tfpr.Contrastnon-contrast CT**	**-0.054**	**0.466**	**-0.116**	**0.908**	**[-0.968, 0.860]**
**Variance components: between-studies Std. Dev and correlation matrix**					
	**SD**		**tsens**		**tfpr**
**tsens**	**0.486**		**–**		**0.355**
**tfpr**	**0.492**		**0.355**		**–**
**TYPE OF MODEL**<br>**Fixed-effects coefficients**					
	**Estimate**	**SE**	**z**	***p*-value**	**CI 95%**
**tsens.(Intercept)**	**1.422**	**0.137**	**10.394**	**0.000**	**[1.154, 1.690]**
**tsens.Modelrad**	**-0.174**	**0.187**	**-0.930**	**0.352**	**[-0.542, 0.193]**
**tfpr.(Intercept)**	**-0.970**	**0.128**	**-7.600**	**0.000**	**[-1.220, -0.720]**
**tfpr.Modelrad**	**0.025**	**0.182**	**0.135**	**0.892**	**[-0.332, 0.381]**
**Variance components: between-studies Std. Dev and correlation matrix**					
	**SD**		**tsens**		**tfpr**
**tsens**	**0.498**		**–**		**0.389**
**tfpr**	**0.498**		**0.389**		**–**
**AI METHODOLOGY**<br>**Fixed-effects coefficients**					
	**Estimate**	**SE**	**z**	***p*-value**	**CI 95%**

**tsens.(Intercept)**	**1.612**	**0.190**	**8.507**	**0.000**	**[1.241, 1.984]**
**tsens.TypeML**	**-0.370**	**0.215**	**-1.718**	**0.086**	**[-0.792, 0.052]**
**tfpr.(Intercept)**	**-0.915**	**0.193**	**-4.749**	**0.000**	**[-1.292, -0.537]**
**tfpr.TypeML**	**-0.055**	**0.219**	**-0.252**	**0.801**	**[-0.484, 0.374]**
**Variance components: between-studies Std. Dev and correlation matrix**					
	**SD**		**tsens**		**tfpr**
**tsens**	**0.468**		**–**		**0.362**
**tfpr**	**0.502**		**0.362**		**–**

AI, artificial intelligence; CI, confidence interval; ML, machine learning; rad, model including only radiomic features; SE, standard error; SD, Standard deviation; z, standard score in a gaussian distribution; tsens, logarithmic transformation of sensitivity; tfpr, logarithmic transformation of false positive rate.

Supplementary Table S10. Detailed comparison of the methodological scope and contributions of the systematic reviews and meta-analyses conducted by Nguyen et al. and Chen et al.

Feature	Present Study	Chen et al. 2024 [125]	Nguyen et al. 2023 [126]
Scope	AI-based prediction of EGFR, ALK, and KRAS mutation status in NSCLC	Prediction of EGFR, ALK, KRAS, and BRAF mutation status in lung cancer	AI-based prediction of EGFR mutation status in NSCLC
Number of studies (meta-analysis)	51 studies / 111 models	128 studies	35 studies
Meta-analysis unit	Model-level: each model analyzed independently	Study-level	Study-level
Imaging modalities	CT only (standard of care in NSCLC)	CT, PET-CT, MRI	CT, PET-CT, MRI
Inclusion of clinical data	Explicit stratification: clinical-only, radiomics-only, and combined models	Integrated, but not separated analytically	Partially considered
Primary outcomes	Sensitivity and false positive rate, DOR	c-index, sensitivity, specificity	AUC, sensitivity, specificity
Statistical methods	Bivariate random-effects model (Reitsma); subgroup meta-regression	Random-effects model; bivariate mixed-effects model	Bivariate random-effects model

**Mutation subtypes**	**EGFR, ALK, KRAS (most clinically actionable)**	**EGFR, ALK, KRAS, BRAF**	**EGFR only**
**Clinical perspective**	**Central: results discussed in context of clinical applicability**	**Not explicitly emphasized**	**Minimal**
**Standardization of methodology**	**Homogeneous imaging modality, standardized model extraction criteria**	**High heterogeneity across models and imaging types**	**Moderate heterogeneity**

References

1 Chang C, Zhou S, Yu H et al (2021) A clinically practical radiomics-clinical combined model based on PET/CT data and nomogram predicts EGFR mutation in lung adenocarcinoma. Eur Radiol 31:6259–6268. https://doi.org/10.1007/s00330-020-07676-x

2 Chang C, Sun X, Wang G et al (2021) A Machine Learning Model Based on PET/CT Radiomics and Clinical Characteristics Predicts ALK Rearrangement Status in Lung Adenocarcinoma. Front Oncol 11:603882. https://doi.org/10.3389/fonc.2021.603882

3 Cheng Y, Wang H, Yuan W et al (2023) Combined radiomics of primary tumour and bone metastasis improve the prediction of EGFR mutation status and response to EGFR-TKI therapy for NSCLC. Phys Med 116:103177. https://doi.org/10.1016/j.ejmp.2023.103177

4 Dong Y, Hou L, Yang W et al (2021) Multi-channel multi-task deep learning for predicting EGFR and KRAS mutations of non-small cell lung cancer on CT images. Quant Imaging Med Surg 11:2354–2375. https://doi.org/10.21037/qims-20-600

5 Dong Y, Jiang Z, Li C et al (2022) Development and validation of novel radiomics-based nomograms for the prediction of EGFR mutations and Ki-67 proliferation index in non-small cell lung cancer. Quant Imaging Med Surg 12:2658–2671. https://doi.org/10.21037/qims-21-980

6 Feng Y, Song F, Zhang P et al (2022) Prediction of EGFR Mutation Status in Non-Small Cell Lung Cancer Based on Ensemble Learning. Front Pharmacol 13:897597. https://doi.org/10.3389/fphar.2022.897597

7 Gao J, Niu R, Shi Y et al (2023) The predictive value of [(18)F]FDG PET/CT radiomics combined with clinical features for EGFR mutation status in different clinical staging of lung adenocarcinoma. EJNMMI Res 13:26. https://doi.org/10.1186/s13550-023-00977-4

8 Gong J, Fu F, Ma X et al (2023) Hybrid deep multi-task learning radiomics approach for predicting EGFR mutation status of non-small cell lung cancer in CT images. Phys Med Biol 68https://doi.org/10.1088/1361-6560/ad0d43

9 Hinzpeter R, Kulanthaivelu R, Kohan A et al (2024) Predictive [(18)F]-FDG PET/CT-Based Radiogenomics Modelling of Driver Gene Mutations in Non-small Cell Lung Cancer. Acad Radiol 31:5314–5323. https://doi.org/10.1016/j.acra.2024.06.038

10 Hu Y, Geng Y, Wang H et al (2024) Improved Prediction of Epidermal Growth Factor Receptor Status by Combined Radiomics of Primary Nonsmall-Cell Lung Cancer and Distant Metastasis. J Comput Assist Tomogr 48:780–788. https://doi.org/10.1097/RCT.0000000000001591

11 Huo JW, Luo TY, Diao L et al (2022) Using combined CT-clinical radiomics models to identify epidermal growth factor receptor mutation subtypes in lung adenocarcinoma. Front Oncol 12:846589. https://doi.org/10.3389/fonc.2022.846589

12 Jia TY, Xiong JF, Li XY et al (2019) Identifying EGFR mutations in lung adenocarcinoma by noninvasive imaging using radiomics features and random forest modeling. Eur Radiol 29:4742–4750. https://doi.org/10.1007/s00330-019-06024-y

13 Jiang M, Yang P, Li J et al (2022) Computed tomography-based radiomics quantification predicts epidermal growth factor receptor mutation status and efficacy of first-line targeted therapy in lung adenocarcinoma. Front Oncol 12:985284. https://doi.org/10.3389/fonc.2022.985284

14 Kohan A, Hinzpeter R, Kulanthaivelu R et al (2024) Contrast Enhanced CT Radiogenomics in a Retrospective NSCLC Cohort: Models, Attempted Validation of a Published Model and the Relevance of the Clinical Context. Acad Radiol 31:2953–2961. https://doi.org/10.1016/j.acra.2024.01.031

15 Le NQK, Kha QH, Nguyen VH, Chen YC, Cheng SJ, Chen CY (2021) Machine Learning-Based Radiomics Signatures for EGFR and KRAS Mutations Prediction in Non-Small-Cell Lung Cancer. Int J Mol Sci 22:9254. https://doi.org/10.3390/ijms22179254

16 Li Y, Lu L, Xiao M et al (2018) CT Slice Thickness and Convolution Kernel Affect Performance of a Radiomic Model for Predicting EGFR Status in Non-Small Cell Lung Cancer: A Preliminary Study. Sci Rep 8:17913. https://doi.org/10.1038/s41598-018-36421-0

17 Li S, Ding C, Zhang H, Song J, Wu L (2019) Radiomics for the prediction of EGFR mutation subtypes in non-small cell lung cancer. Med Phys 46:4545–4552. https://doi.org/10.1002/mp.13747

18 Li S, Luo T, Ding C, Huang Q, Guan Z, Zhang H (2020) Detailed identification of epidermal growth factor receptor mutations in lung adenocarcinoma: Combining radiomics with machine learning. Med Phys 47:3458–3466. https://doi.org/10.1002/mp.14238

19 Li S, Li Y, Zhao M, Wang P, Xin J (2022) Combination of (18)F-Fluorodeoxyglucose PET/CT Radiomics and Clinical Features for Predicting Epidermal Growth Factor Receptor Mutations in Lung Adenocarcinoma. Korean J Radiol 23:921–930. https://doi.org/10.3348/kjr.2022.0295

20 Liu Q, Sun D, Li N et al (2020) Predicting EGFR mutation subtypes in lung adenocarcinoma using (18)F-FDG PET/CT radiomic features. Transl Lung Cancer Res 9:549–562. https://doi.org/10.21037/tlcr.2020.04.17

21 Liu Y, Zhou J, Wu J et al (2022) Development and Validation of Machine Learning Models to Predict Epidermal Growth Factor Receptor Mutation in Non-Small Cell Lung Cancer: A Multi-Center Retrospective Radiomics Study. Cancer Control 29:10732748221092926. https://doi.org/10.1177/10732748221092926

22 Lu L, Sun SH, Yang H et al (2020) Radiomics Prediction of EGFR Status in Lung Cancer-Our Experience in Using Multiple Feature Extractors and The Cancer Imaging Archive Data. Tomography 6:223–230. https://doi.org/10.18383/j.tom.2020.00017

23 Lu J, Ji X, Wang L et al (2022) Machine Learning-Based Radiomics for Prediction of Epidermal Growth Factor Receptor Mutations in Lung Adenocarcinoma. Dis Markers 2022:2056837. https://doi.org/10.1155/2022/2056837

24 Ma DN, Gao XY, Dan YB et al (2020) Evaluating Solid Lung Adenocarcinoma Anaplastic Lymphoma Kinase Gene Rearrangement Using Noninvasive Radiomics Biomarkers. Onco Targets Ther 13:6927–6935. https://doi.org/10.2147/OTT.S257798

25 Ma JW, Jiang X, Wang YM et al (2024) Dual-energy CT-based radiomics in predicting EGFR mutation status non-invasively in lung adenocarcinoma. Heliyon 10:e24372. https://doi.org/10.1016/j.heliyon.2024.e24372

26 Nair JKR, Saeed UA, McDougall CC et al (2021) Radiogenomic Models Using Machine Learning Techniques to Predict EGFR Mutations in Non-Small Cell Lung Cancer. Can Assoc Radiol J 72:109–119. https://doi.org/10.1177/0846537119899526

27 Ninomiya K, Arimura H, Chan WY et al (2021) Robust radiogenomics approach to the identification of EGFR mutations among patients with NSCLC from three different

countries using topologically invariant Betti numbers. PLoS One 16:e0244354. https://doi.org/10.1371/journal.pone.0244354

28 Ninomiya K, Arimura H, Tanaka K et al (2023) Three-dimensional topological radiogenomics of epidermal growth factor receptor Del19 and L858R mutation subtypes on computed tomography images of lung cancer patients. Comput Methods Programs Biomed 236:107544. https://doi.org/10.1016/j.cmpb.2023.107544

29 Rios Velazquez E, Parmar C, Liu Y et al (2017) Somatic Mutations Drive Distinct Imaging Phenotypes in Lung Cancer. Cancer Res 77:3922–3930. https://doi.org/10.1158/0008-5472.CAN-17-0122

30 Rossi G, Barabino E, Fedeli A et al (2021) Radiomic Detection of EGFR Mutations in NSCLC. Cancer Res 81:724–731. https://doi.org/10.1158/0008-5472.CAN-20-0999

31 Shao X, Ge X, Gao J et al (2024) Transfer learning-based PET/CT three-dimensional convolutional neural network fusion of image and clinical information for prediction of EGFR mutation in lung adenocarcinoma. BMC Med Imaging 24:54. https://doi.org/10.1186/s12880-024-01232-5

32 Song L, Zhu Z, Mao L et al (2020) Clinical, Conventional CT and Radiomic Feature-Based Machine Learning Models for Predicting ALK Rearrangement Status in Lung Adenocarcinoma Patients. Front Oncol 10:369. https://doi.org/10.3389/fonc.2020.00369

33 Tan JL, Xia L, Sun SG, Zeng H, Lu DY, Cheng XJ (2023) Prediction of EGFR mutation status in lung adenocarcinoma based on (18)F-FDG PET/CT radiomic features. Am J Nucl Med Mol Imaging 13:230–244.

34 Tu W, Sun G, Fan L et al (2019) Radiomics signature: A potential and incremental predictor for EGFR mutation status in NSCLC patients, comparison with CT morphology. Lung Cancer 132:28–35. https://doi.org/10.1016/j.lungcan.2019.03.025

35 Wang C, Ma J, Shao J et al (2022) Predicting EGFR and PD-L1 Status in NSCLC Patients Using Multitask AI System Based on CT Images. Front Immunol 13:813072. https://doi.org/10.3389/fimmu.2022.813072

36 Wang J, Lv X, Huang W et al (2022) Establishment and Optimization of Radiomics Algorithms for Prediction of KRAS Gene Mutation by Integration of NSCLC Gene Mutation Mutual Exclusion Information. Front Pharmacol 13:862581. https://doi.org/10.3389/fphar.2022.862581

37 Wang Y, Yang G, Gao X, Li L, Zhu H, Yi H (2024) Subregion-specific (18)F-FDG PET-CT radiomics for the pre-treatment prediction of EGFR mutation status in solid lung adenocarcinoma. Am J Nucl Med Mol Imaging 14:134–143. https://doi.org/10.62347/DDRR4923

38 Wang XY, Wu SH, Ren J, Zeng Y, Guo LL (2025) Predicting Gene Comutation of EGFR and TP53 by Radiomics and Deep Learning in Patients With Lung Adenocarcinomas. J Thorac Imaging 40:e0817. https://doi.org/10.1097/RTI.0000000000000817

39 Weng Q, Hui J, Wang H et al (2021) Radiomic Feature-Based Nomogram: A Novel Technique to Predict EGFR-Activating Mutations for EGFR Tyrosin Kinase Inhibitor Therapy. Front Oncol 11:590937. https://doi.org/10.3389/fonc.2021.590937

40 Wu S, Shen G, Mao J, Gao B (2020) CT Radiomics in Predicting EGFR Mutation in Non-small Cell Lung Cancer: A Single Institutional Study. Front Oncol 10:542957. https://doi.org/10.3389/fonc.2020.542957

41 Wu J, Meng H, Zhou L et al (2024) Habitat radiomics and deep learning fusion nomogram to predict EGFR mutation status in stage I non-small cell lung cancer: a multicenter study. Sci Rep 14:15877. https://doi.org/10.1038/s41598-024-66751-1

42 Yang C, Chen W, Gong G, Li Z, Qiu Q, Yin Y (2020) Application of CT radiomics features to predict the EGFR mutation status and therapeutic sensitivity to TKIs of advanced lung adenocarcinoma. Transl Cancer Res 9:6683–6690. https://doi.org/10.21037/tcr-20-1216

43 Yang X, Liu M, Ren Y et al (2022) Using contrast-enhanced CT and non-contrast-enhanced CT to predict EGFR mutation status in NSCLC patients-a radiomics nomogram analysis. Eur Radiol 32:2693–2703. https://doi.org/10.1007/s00330-021-08366-y

44 Zhang L, Chen B, Liu X et al (2018) Quantitative Biomarkers for Prediction of Epidermal Growth Factor Receptor Mutation in Non-Small Cell Lung Cancer. Transl Oncol 11:94–101. https://doi.org/10.1016/j.tranon.2017.10.012

45 Zhang M, Bao Y, Rui W et al (2020) Performance of (18)F-FDG PET/CT Radiomics for Predicting EGFR Mutation Status in Patients With Non-Small Cell Lung Cancer. Front Oncol 10:568857. https://doi.org/10.3389/fonc.2020.568857

46 Zhang B, Qi S, Pan X et al (2020) Deep CNN Model Using CT Radiomics Feature Mapping Recognizes EGFR Gene Mutation Status of Lung Adenocarcinoma. Front Oncol 10:598721. https://doi.org/10.3389/fonc.2020.598721

47 Zhang G, Cao Y, Zhang J et al (2021) Predicting EGFR mutation status in lung adenocarcinoma: development and validation of a computed tomography-based radiomics signature. Am J Cancer Res 11:546–560.

48 Zhang G, Man Q, Shang L et al (2024) Using Multi-phase CT Radiomics Features to Predict EGFR Mutation Status in Lung Adenocarcinoma Patients. Acad Radiol 31:2591–2600. https://doi.org/10.1016/j.acra.2023.12.024

49 Zhang G, Shang L, Cao Y et al (2024) Prediction of epidermal growth factor receptor (EGFR) mutation status in lung adenocarcinoma patients on computed tomography (CT) images using 3-dimensional (3D) convolutional neural network. Quant Imaging Med Surg 14:6048–6059. https://doi.org/10.21037/qims-24-33

50 Zhao HY, Su YX, Zhang LH, Fu P (2022) Prediction model based on 18F-FDG PET/CT radiomic features and clinical factors of EGFR mutations in lung adenocarcinoma. Neoplasma 69:233–241. https://doi.org/10.4149/neo_2021_201222N1388

51 Zhu H, Song Y, Huang Z et al (2022) Accurate prediction of epidermal growth factor receptor mutation status in early-stage lung adenocarcinoma, using radiomics and clinical features. Asia Pac J Clin Oncol 18:586–594. https://doi.org/10.1111/ajco.13641

52 Aguloglu N, Aksu A, Akyol M, Katgi N, Doksoz TC (2022) Importance of pretreatment 18f-fdg pet/ct texture analysis in predicting egfr and alk mutation in patients with non-small cell lung cancer. Nuklearmedizin 61:433–439. https://doi.org/10.1055/a-1868-4918

53 Aerts HJ, Grossmann P, Tan Y et al (2016) Defining a Radiomic Response Phenotype: A Pilot Study using targeted therapy in NSCLC. Sci Rep 6:33860. https://doi.org/10.1038/srep33860

54 Agazzi GM, Ravanelli M, Roca E et al (2021) CT texture analysis for prediction of EGFR mutational status and ALK rearrangement in patients with non-small cell lung cancer. Radiol Med 126:786–794. https://doi.org/10.1007/s11547-020-01323-7

55 Aide N, Weyts K, Lasnon C (2022) Prediction of the Presence of Targetable Molecular Alteration(s) with Clinico-Metabolic (18) F-FDG PET Radiomics in Non-Asian

Lung Adenocarcinoma Patients. Diagnostics (Basel) 12:2448. https://doi.org/10.3390/diagnostics12102448

56 Chen W, Hua Y, Mao D et al (2021) A Computed Tomography-Derived Radiomics Approach for Predicting Uncommon EGFR Mutation in Patients With NSCLC. Front Oncol 11:722106. https://doi.org/10.3389/fonc.2021.722106

57 Chen Q, Li Y, Cheng Q et al (2022) EGFR Mutation Status and Subtypes Predicted by CT-Based 3D Radiomic Features in Lung Adenocarcinoma. Onco Targets Ther 15:597–608. https://doi.org/10.2147/OTT.S352619

58 Chen Z, Gao S, Ding C et al (2024) CT-based non-invasive identification of the most common gene mutation status in patients with non-small cell lung cancer. Med Phys 51:1872–1882. https://doi.org/10.1002/mp.16744

59 Choe J, Lee SM, Kim W et al (2021) CT radiomics-based prediction of anaplastic lymphoma kinase and epidermal growth factor receptor mutations in lung adenocarcinoma. Eur J Radiol 139:109710. https://doi.org/10.1016/j.ejrad.2021.109710

60 Dang Y, Wang R, Qian K, Lu J, Zhang H, Zhang Y (2021) Clinical and radiological predictors of epidermal growth factor receptor mutation in nonsmall cell lung cancer. J Appl Clin Med Phys 22:271–280. https://doi.org/10.1002/acm2.13107

61 Digumarthy SR, Padole AM, Gullo RL, Sequist LV, Kalra MK (2019) Can CT radiomic analysis in NSCLC predict histology and EGFR mutation status? Medicine (Baltimore) 98:e13963. https://doi.org/10.1097/MD.0000000000013963

62 Hao P, Deng BY, Huang CT et al (2022) Predicting anaplastic lymphoma kinase rearrangement status in patients with non-small cell lung cancer using a machine learning algorithm that combines clinical features and CT images. Front Oncol 12:994285. https://doi.org/10.3389/fonc.2022.994285

63 He R, Yang X, Li T et al (2022) A Machine Learning-Based Predictive Model of Epidermal Growth Factor Mutations in Lung Adenocarcinomas. Cancers (Basel) 14:4664. https://doi.org/10.3390/cancers14194664

64 Hong D, Xu K, Zhang L, Wan X, Guo Y (2020) Radiomics Signature as a Predictive Factor for EGFR Mutations in Advanced Lung Adenocarcinoma. Front Oncol 10:28. https://doi.org/10.3389/fonc.2020.00028

65 Huang Q, Lu L, Dercle L et al (2018) Interobserver variability in tumor contouring affects the use of radiomics to predict mutational status. J Med Imaging (Bellingham) 5:011005. https://doi.org/10.1117/1.JMI.5.1.011005

66 Huang W, Wang J, Wang H et al (2022) PET/CT Based EGFR Mutation Status Classification of NSCLC Using Deep Learning Features and Radiomics Features. Front Pharmacol 13:898529. https://doi.org/10.3389/fphar.2022.898529

67 Huang X, Sun Y, Tan M et al (2022) Three-Dimensional Convolutional Neural Network-Based Prediction of Epidermal Growth Factor Receptor Expression Status in Patients With Non-Small Cell Lung Cancer. Front Oncol 12:772770. https://doi.org/10.3389/fonc.2022.772770

68 Hou D, Li W, Wang S et al (2021) Different Clinicopathologic and Computed Tomography Imaging Characteristics of Primary and Acquired EGFR T790M Mutations in Patients with Non-Small-Cell Lung Cancer. Cancer Manag Res 13:6389–6401. https://doi.org/10.2147/CMAR.S323972

69 Jiang M, Zhang Y, Xu J et al (2019) Assessing EGFR gene mutation status in non-small cell lung cancer with imaging features from PET/CT. Nucl Med Commun 40:842–849. https://doi.org/10.1097/MNM.0000000000001043

70 Kawazoe Y, Shiinoki T, Fujimoto K et al (2023) Investigation of the combination of intratumoral and peritumoral radiomic signatures for predicting epidermal growth factor receptor mutation in lung adenocarcinoma. J Appl Clin Med Phys 24:e13980. https://doi.org/10.1002/acm2.13980

71 Kawazoe Y, Shiinoki T, Fujimoto K et al (2023) Comparison of the radiomics-based predictive models using machine learning and nomogram for epidermal growth factor receptor mutation status and subtypes in lung adenocarcinoma. Phys Eng Sci Med 46:395–403. https://doi.org/10.1007/s13246-023-01232-9

72 Kim S, Lim JH, Kim CH et al (2024) Deep learning-radiomics integrated noninvasive detection of epidermal growth factor receptor mutations in non-small cell lung cancer patients. Sci Rep 14:922. https://doi.org/10.1038/s41598-024-51630-6

73 Koyasu S, Nishio M, Isoda H, Nakamoto Y, Togashi K (2020) Usefulness of gradient tree boosting for predicting histological subtype and EGFR mutation status of non-small cell lung cancer on (18)F FDG-PET/CT. Ann Nucl Med 34:49–57. https://doi.org/10.1007/s12149-019-01414-0

74 Li XY, Xiong JF, Jia TY et al (2018) Detection of epithelial growth factor receptor (EGFR) mutations on CT images of patients with lung adenocarcinoma using radiomics and/or multi-level residual convolutionary neural networks. J Thorac Dis 10:6624–6635. https://doi.org/10.21037/jtd.2018.11.03

75 Li X, Yin G, Zhang Y et al (2019) Predictive Power of a Radiomic Signature Based on (18)F-FDG PET/CT Images for EGFR Mutational Status in NSCLC. Front Oncol 9:1062. https://doi.org/10.3389/fonc.2019.01062

76 Li H, Gao C, Sun Y et al (2021) Radiomics Analysis to Enhance Precise Identification of Epidermal Growth Factor Receptor Mutation Based on Positron Emission Tomography Images of Lung Cancer Patients. J Biomed Nanotechnol 17:691–702. https://doi.org/10.1166/jbn.2021.3056

77 Li X, Chen J, Zhang C, Han Z, Zheng X, Cao D (2023) Application value of CT radiomic nomogram in predicting T790M mutation of lung adenocarcinoma. BMC Pulm Med 23:339. https://doi.org/10.1186/s12890-023-02609-y

78 Li S, Hu Y, Tian C et al (2025) Prediction of EGFR-TP53 genes co-mutations in patients with lung adenocarcinoma (LUAD) by (18)F-FDG PET/CT radiomics. Clin Transl Oncol 27:1506–1515. https://doi.org/10.1007/s12094-024-03685-0

79 Liu Y, Kim J, Balagurunathan Y et al (2016) Radiomic Features Are Associated With EGFR Mutation Status in Lung Adenocarcinomas. Clin Lung Cancer 17:441–448 e446. https://doi.org/10.1016/j.cllc.2016.02.001

80 Liu G, Xu Z, Ge Y et al (2020) 3D radiomics predicts EGFR mutation, exon-19 deletion and exon-21 L858R mutation in lung adenocarcinoma. Transl Lung Cancer Res 9:1212–1224. https://doi.org/10.21037/tlcr-20-122

81 Liu Z, Zhang T, Lin L, Long F, Guo H, Han L (2023) Applications of radiomics-based analysis pipeline for predicting epidermal growth factor receptor mutation status. Biomed Eng Online 22:17. https://doi.org/10.1186/s12938-022-01049-9

82 Liu X, Xu T, Wang S et al (2023) CT-based radiomic phenotypes of lung adenocarcinoma: a preliminary comparative analysis with targeted next-generation sequencing. Front Med (Lausanne) 10:1191019. https://doi.org/10.3389/fmed.2023.1191019

83 Lu X, Li M, Zhang H et al (2020) A novel radiomic nomogram for predicting epidermal growth factor receptor mutation in peripheral lung adenocarcinoma. Phys Med Biol 65:055012. https://doi.org/10.1088/1361-6560/ab6f98

84 Lu J, Ji X, Liu X et al (2024) Machine learning-based radiomics strategy for prediction of acquired EGFR T790M mutation following treatment with EGFR-TKI in NSCLC. Sci Rep 14:446. https://doi.org/10.1038/s41598-023-50984-7

85 Mahajan A, Kania V, Agarwal U et al (2024) Deep-Learning-Based Predictive Imaging Biomarker Model for EGFR Mutation Status in Non-Small Cell Lung Cancer from CT Imaging. Cancers (Basel) 16:1130. https://doi.org/10.3390/cancers16061130

86 Mei D, Luo Y, Wang Y, Gong J (2018) CT texture analysis of lung adenocarcinoma: can Radiomic features be surrogate biomarkers for EGFR mutation statuses. Cancer Imaging 18:52. https://doi.org/10.1186/s40644-018-0184-2

87 Mu W, Jiang L, Zhang J et al (2020) Non-invasive decision support for NSCLC treatment using PET/CT radiomics. Nat Commun 11:5228. https://doi.org/10.1038/s41467-020-19116-x

88 Njoto EN, Jasminarti Dwi Kusumawardani IA, Rai IBN (2023) Predicting EGFR Mutation in Lung Adenocarcinoma: Development and Validation of the EGFR Mutation Predictive Score (EMPS) in Bali, Indonesia. Asian Pac J Cancer Prev 24:2903–2910. https://doi.org/10.31557/APJCP.2023.24.8.2903

89 Omura K, Murakami Y, Hashimoto K et al (2023) Detection of EGFR mutations in early-stage lung adenocarcinoma by machine learning-based radiomics. Transl Cancer Res 12:837–847. https://doi.org/10.21037/tcr-22-2683

90 Ottaiano A, Grassi F, Sirica R et al (2024) Associations between Radiomics and Genomics in Non-Small Cell Lung Cancer Utilizing Computed Tomography and Next-Generation Sequencing: An Exploratory Study. Genes (Basel) 15:803. https://doi.org/10.3390/genes15060803

91 Rinaldi L, Guerini Rocco E, Spitaleri G et al (2023) Association between Contrast-Enhanced Computed Tomography Radiomic Features, Genomic Alterations and Prognosis in Advanced Lung Adenocarcinoma Patients. Cancers (Basel) 15:4553. https://doi.org/10.3390/cancers15184553

92 Ruan D, Fang J, Teng X (2022) Efficient 18F-Fluorodeoxyglucose positron emission tomography/computed tomography-based machine learning model for predicting epidermal growth factor receptor mutations in non-small cell lung cancer. Q J Nucl Med Mol Imaging. 10.23736/s1824-4785.22.03441-0https://doi.org/10.23736/s1824-4785.22.03441-0

93 Shang Y, Chen W, Li G et al (2023) Computed Tomography-derived intratumoral and peritumoral radiomics in predicting EGFR mutation in lung adenocarcinoma. Radiol Med 128:1483–1496. https://doi.org/10.1007/s11547-023-01722-6

94 Shao J, Ma J, Zhang S et al (2022) Radiogenomic System for Non-Invasive Identification of Multiple Actionable Mutations and PD-L1 Expression in Non-Small Cell Lung Cancer Based on CT Images. Cancers (Basel) 14:4823. https://doi.org/10.3390/cancers14194823

95 Shiri I, Maleki H, Hajianfar G et al (2020) Next-Generation Radiogenomics Sequencing for Prediction of EGFR and KRAS Mutation Status in NSCLC Patients Using Multimodal Imaging and Machine Learning Algorithms. Mol Imaging Biol 22:1132–1148. https://doi.org/10.1007/s11307-020-01487-8

96 Shiri I, Amini M, Nazari M et al (2022) Impact of feature harmonization on radiogenomics analysis: Prediction of EGFR and KRAS mutations from non-small cell lung cancer PET/CT images. Comput Biol Med 142:105230. https://doi.org/10.1016/j.compbiomed.2022.105230

97 Song J, Ding C, Huang Q et al (2021) Deep learning predicts epidermal growth factor receptor mutation subtypes in lung adenocarcinoma. Med Phys 48:7891–7899. https://doi.org/10.1002/mp.15307

98 Trivizakis E, Souglakos J, Karantanas A, Marias K (2021) Deep Radiotranscriptomics of Non-Small Cell Lung Carcinoma for Assessing Molecular and Histology Subtypes with a Data-Driven Analysis. Diagnostics (Basel) 11:2383. https://doi.org/10.3390/diagnostics11122383

99 Wang X, Kong C, Xu W et al (2019) Decoding tumor mutation burden and driver mutations in early stage lung adenocarcinoma using CT-based radiomics signature. Thorac Cancer 10:1904–1912. https://doi.org/10.1111/1759-7714.13163

100 Wang C, Xu X, Shao J et al (2021) Deep Learning to Predict EGFR Mutation and PD-L1 Expression Status in Non-Small-Cell Lung Cancer on Computed Tomography Images. J Oncol 2021:5499385. https://doi.org/10.1155/2021/5499385

101 Wang B, Bao C, Wang X et al (2024) Inter-equipment validation of PET-based radiomics for predicting EGFR mutation statuses in patients with non-small cell lung cancer. Clin Radiol 79:571–578. https://doi.org/10.1016/j.crad.2023.12.030

102 Wang C, Zhang R, Sun X, Xing L (2024) Prediction of epidermal growth factor receptor mutation status by textural features in stage IV lung adenocarcinoma. Memo -

Magazine of European Medical Oncology 17:100–106. https://doi.org/10.1007/s12254-024-00961-1

103 Xiao Z, Cai H, Wang Y et al (2023) Deep learning for predicting epidermal growth factor receptor mutations of non-small cell lung cancer on PET/CT images. Quant Imaging Med Surg 13:1286–1299. https://doi.org/10.21037/qims-22-760

104 Xiong W, Yu X, Zhou T, Huang H, Zhao Z, Wang T (2024) A Radiomics-clinical Nomogram based on CT Radiomics to Predict Acquired T790M Mutation Status in Non-small Cell Lung Cancer Patients. Curr Med Imaging 20:17. https://doi.org/10.2174/0115734056283623240215102037

105 Xu N, Wang J, Dai G et al (2024) EfficientNet-Based System for Detecting EGFR-Mutant Status and Predicting Prognosis of Tyrosine Kinase Inhibitors in Patients with NSCLC. J Imaging Inform Med 37:1086–1099. https://doi.org/10.1007/s10278-024-01022-z

106 Yamazaki M, Yagi T, Tominaga M, Minato K, Ishikawa H (2022) Role of intratumoral and peritumoral CT radiomics for the prediction of EGFR gene mutation in primary lung cancer. Br J Radiol 95:20220374. https://doi.org/10.1259/bjr.20220374

107 Yang B, Ji HS, Zhou CS et al (2020) (18)F-fluorodeoxyglucose positron emission tomography/computed tomography-based radiomic features for prediction of epidermal growth factor receptor mutation status and prognosis in patients with lung adenocarcinoma. Transl Lung Cancer Res 9:563–574. https://doi.org/10.21037/tlcr-19-592

108 Yang L, Xu P, Li M et al (2022) PET/CT Radiomic Features: A Potential Biomarker for EGFR Mutation Status and Survival Outcome Prediction in NSCLC Patients Treated With TKIs. Front Oncol 12:894323. https://doi.org/10.3389/fonc.2022.894323

109 Yang X, Fang C, Li C et al (2022) Can CT Radiomics Detect Acquired T790M Mutation and Predict Prognosis in Advanced Lung Adenocarcinoma With Progression After First- or Second-Generation EGFR TKIs? Front Oncol 12:904983. https://doi.org/10.3389/fonc.2022.904983

110 Yao X, Zhu Y, Huang Z et al (2024) Fusion of shallow and deep features from (18)F-FDG PET/CT for predicting EGFR-sensitizing mutations in non-small cell lung cancer. Quant Imaging Med Surg 14:5460–5472. https://doi.org/10.21037/qims-23-1028

111 Yip SSF, Parmar C, Kim J, Huynh E, Mak RH, Aerts H (2017) Impact of experimental design on PET radiomics in predicting somatic mutation status. Eur J Radiol 97:8–15. https://doi.org/10.1016/j.ejrad.2017.10.009

112 Zhang J, Zhao X, Zhao Y et al (2020) Value of pre-therapy (18)F-FDG PET/CT radiomics in predicting EGFR mutation status in patients with non-small cell lung cancer. Eur J Nucl Med Mol Imaging 47:1137–1146. https://doi.org/10.1007/s00259-019-04592-1

113 Zhang T, Xu Z, Liu G et al (2021) Simultaneous Identification of EGFR,KRAS,ERBB2, and TP53 Mutations in Patients with Non-Small Cell Lung Cancer by Machine Learning-Derived Three-Dimensional Radiomics. Cancers (Basel) 13https://doi.org/10.3390/cancers13081814

114 Zhang T, Liu Z, Lin L et al (2023) Detection of the gene mutation of epidermal growth factor receptor in lung adenocarcinoma by radiomic features from a small amount of PET data. Nucl Med Commun 44:795–802. https://doi.org/10.1097/MNM.0000000000001718

115 Zhang R, Shi K, Hohenforst-Schmidt W et al (2023) Ability of (18)F-FDG Positron Emission Tomography Radiomics and Machine Learning in Predicting KRAS Mutation Status in Therapy-Naive Lung Adenocarcinoma. Cancers (Basel) 15:3684. https://doi.org/10.3390/cancers15143684

116 Zhang X, Zhang G, Qiu X et al (2024) Non-invasive decision support for clinical treatment of non-small cell lung cancer using a multiscale radiomics approach. Radiother Oncol 191:110082. https://doi.org/10.1016/j.radonc.2024.110082

117 Zhang X, Zhang G, Qiu X et al (2024) Exploring non-invasive precision treatment in non-small cell lung cancer patients through deep learning radiomics across imaging features and molecular phenotypes. Biomark Res 12:12. https://doi.org/10.1186/s40364-024-00561-5

118 Zhao W, Yang J, Ni B et al (2019) Toward automatic prediction of EGFR mutation status in pulmonary adenocarcinoma with 3D deep learning. Cancer Med 8:3532–3543. https://doi.org/10.1002/cam4.2233

119 Zhao W, Wu Y, Xu Y et al (2019) The Potential of Radiomics Nomogram in Non-invasively Prediction of Epidermal Growth Factor Receptor Mutation Status and Subtypes in Lung Adenocarcinoma. Front Oncol 9:1485. https://doi.org/10.3389/fonc.2019.01485

120 Zhao W, Chen W, Li G et al (2024) GMILT: A Novel Transformer Network That Can Noninvasively Predict EGFR Mutation Status. IEEE Trans Neural Netw Learn Syst 35:7324–7338. https://doi.org/10.1109/TNNLS.2022.3190671

121 Zhu Y, Guo YB, Xu D et al (2021) A computed tomography (CT)-derived radiomics approach for predicting primary co-mutations involving TP53 and epidermal growth factor receptor (EGFR) in patients with advanced lung adenocarcinomas (LUAD). Ann Transl Med 9:545. https://doi.org/10.21037/atm-20-6473

122 Zuo Y, Liu Q, Li N, Li P, Zhang J, Song S (2023) Optimal (18)F-FDG PET/CT radiomics model development for predicting EGFR mutation status and prognosis in lung adenocarcinoma: a multicentric study. Front Oncol 13:1173355. https://doi.org/10.3389/fonc.2023.1173355

123 Zuo Y, Liu L, Chang C et al (2024) Value of multi-center (18)F-FDG PET/CT radiomics in predicting EGFR mutation status in lung adenocarcinoma. Med Phys 51:4872–4887. https://doi.org/10.1002/mp.16947

124 Zuo Y, Liu Q, Li N et al (2024) Explainable (18)F-FDG PET/CT radiomics model for predicting EGFR mutation status in lung adenocarcinoma: a two-center study. J Cancer Res Clin Oncol 150:469. https://doi.org/10.1007/s00432-024-05998-7

125 Chen J, Chen A, Yang S, Liu J, Xie C, Jiang H (2024) Accuracy of machine learning in preoperative identification of genetic mutation status in lung cancer: A systematic review and meta-analysis. Radiother Oncol 196:110325. https://doi.org/10.1016/j.radonc.2024.110325

126 Nguyen HS, Ho DKN, Nguyen NN, Tran HM, Tam KW, Le NQK (2024) Predicting EGFR Mutation Status in Non-Small Cell Lung Cancer Using Artificial Intelligence: A Systematic Review and Meta-Analysis. Acad Radiol 31:660–683. https://doi.org/10.1016/j.acra.2023.03.040
