# Supplementary figures and images for: Prediction of oncogene mutation status in non-small cell lung cancer: a systematic review and meta-analysis with a special focus on artificial intelligence-based methods
Source: Eur Radiol. 2025 Sep 8;36(3):2157–85. doi: 10.1007/s00330-025-11962-x (PMC12963223; doi:10.1007/s00330-025-11962-x)

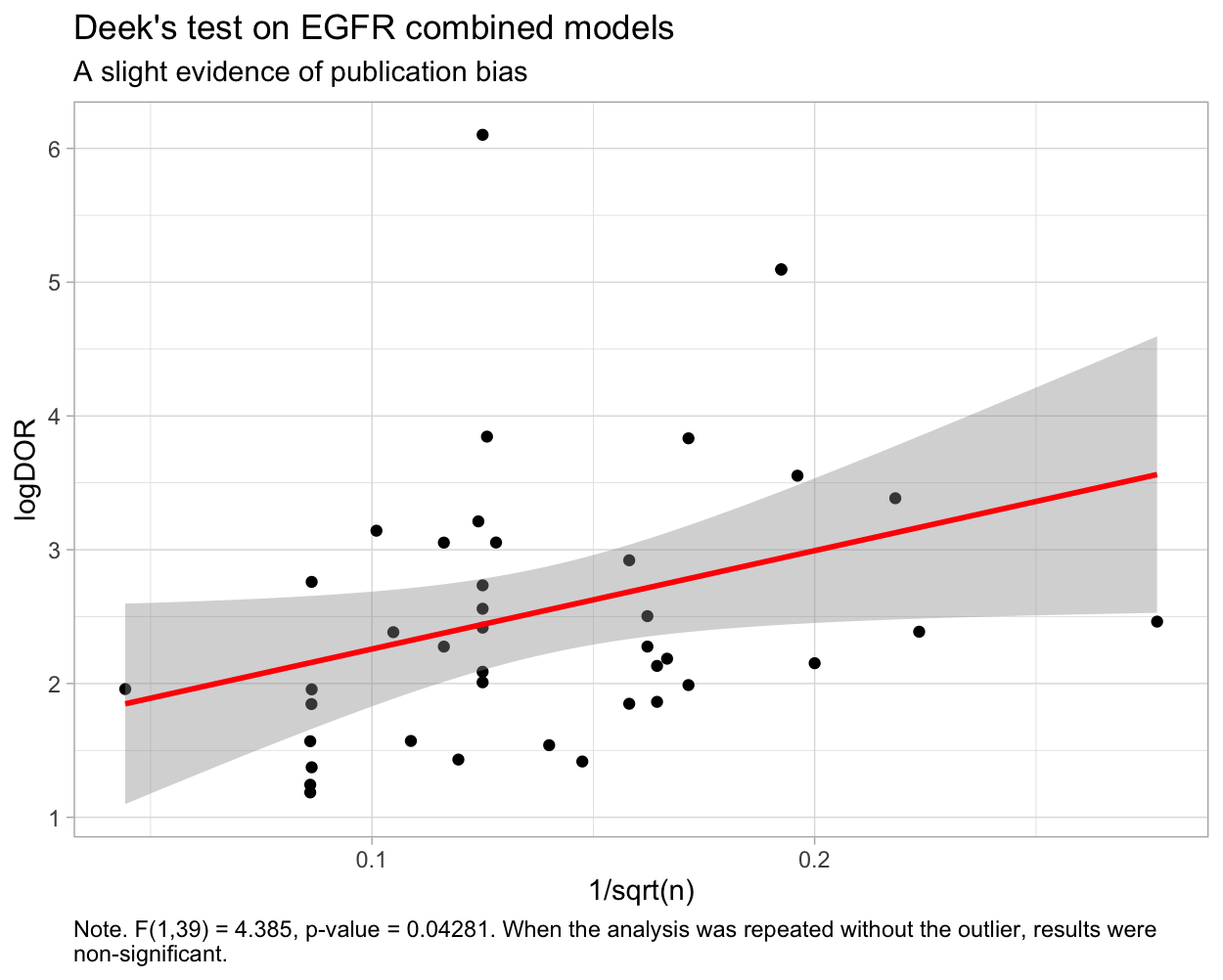

Supplement: Supplementary file 2 — Supplementary Figure S9 [file 330_2025_11962_MOESM2_ESM.tiff]
