# Supplementary material for: Prediction of oncogene mutation status in non-small cell lung cancer: a systematic review and meta-analysis with a special focus on artificial intelligence-based methods
Source: Eur Radiol. 2025 Sep 8;36(3):2157–85. doi: 10.1007/s00330-025-11962-x (PMC12963223; doi:10.1007/s00330-025-11962-x)
Supplement: Supplementary file 3 — Supplementary material [file 330_2025_11962_MOESM3_ESM.docx]

**Supplementary material**

*PROSPERO registration*

This systematic review and meta-analysis was initially registered in PROSPERO ([https://www.crd.york.ac.uk/PROSPERO](https://www.crd.york.ac.uk/PROSPERO/#loginpage)) on July 29, 2022. Further amendments were performed on October 5, 2022. Amendments included:

- EMBASE as an additional search database
- “Predictive model” was replaced by “classification model”

*Inclusion criteria*

Papers were included in the qualitative synthesis (systematic review) if meeting the following inclusion criteria based on Patient, Index test, Comparator, Reference test, Diagnosis of reference (PIRD) questions: 1) being focused on the ability of radiomics to predict oncogene mutation status in NSCLC; 2) radiomics features were extracted from CT or from F-18 fluoro-deoxy-glucose (FDG)/CT scans; 3) a full text was available; 4) were written in English, since it is the international language of science, and therefore used in high-impact journals.

*Exclusion criteria*

Papers describing studies conducted using MRI scans (not the standard of care for NSCLC patients) or performed in phantom or animal models, or published as case reports, editorials, reviews, poster presentations, letters, editorials, or meeting abstracts were excluded. Papers not on the field of interest were also excluded.

For the quantitative synthesis (meta-analysis), the following additional exclusion criteria were applied: 1) oncogene mutation status was not the primary objective of the paper; 2) were focused on specific mutation subtypes; 3) did not apply AI-based methodologies; 4) developed simultaneous detection models or discriminant models; 5) sensitivity or specificity metrics were not available and could not be calculated; 6) were not comparable with the other articles included (model was developed based on intra- and extra-tumor derived radiomics features); 7) only included models developed with a combination of quantitative features extracted from PET/CT or from PET images (strictly adhering to a clinical perspective, PET scanning equipment is not always available and CT remains the standard of care for NSCLC patients); 8) did not reach a sufficient quality score according to the quality assessment (described below).

*Quality assessment*

Classification, image reconstruction, text analysis, and workflow optimization are some of the applications of AI in medical imaging that are addressed by CLAIM, which is modeled after the Standards for Reporting of Diagnostic Accuracy Studies (STARD) guideline [1-4]. CLAIM checklist consists of 42 items divided into the conventional sections included in peer-reviewed scientific articles: title or abstract (1 item), abstract (1 item), introduction (2 items), methods (28 items subdivided into study design [2 items], data [7 items], ground truth [5 items], data partitions [3 items], model [ 3 items], training [3 items] and evaluation [5 items]), results (5 items subdivided into data [2 items] and model performance [3 items]), discussion (2 items) and other information (3 items). The CLAIM guideline offers a roadmap for writers and reviewers with the intention of fostering clear, open, and verifiable scientific discourse on the use of AI in medical imaging [5].

For our quality assessment, a score was calculated for each paper ([total score, 42 - number of “not applicable” fields in each case]). A cut-off value of at least half of the total score after removing the “not applicable” items was established for the inclusion in the quantitative analysis. Therefore, this cut-off value varied for each study depending on the number of items that were applicable from among the 42 total items included in the CLAIM checklist (e.g., a cut-off value of 19 was established for those studies in which only 38 items of the checklist were applicable). **See Supplementary Table 2**. The assessment of the rigor, quality, and generalizability of the work of all enrolled studies was performed by three reviewers.

## Data extraction

Data extracted included the following: (1) study details: first author, publication year, research questions, study design; (2) patient details: the source of data acquisition (single-center/multicenter), sample size, smoking history, age, sex, TNM staging, treatment status (naïve or any treatment received prior image acquisition), histological subtype; (3) imaging details: imaging modality, plain or contrast CT; (4) oncogene mutation status-related information: type of mutation, specific subtype of mutation (if available), sequencing method; sequencing kit (5) radiomics details: segmentation software, type of segmentation (manual, automatic, or semi-automatic), radiomics feature extraction software, number of imaging features extracted, number and name of radiomics features included in final models, features selection methods, type of models constructed (machine learning [ML], deep learning [DL], classical statistical model), final classifier used in machine learning models, clinical variables included in the models (if applicable), and models performance. Two independent reviewers with more than 10 years of experience in biomedical research completed the initial screening and extracted data from all included studies.

## Data analysis

The Reitsma method [6] has the distinct advantage of preserving the two-dimensional nature of the underlying data. It can also produce summary estimates of sensitivity and specificity (false positive rate [FPR, 1-specificity]), recognizing any possible correlation between these two measures. The method uses a random effect approach in which the values of the sensitivity and FPR estimates are obtained with restricted maximum likelihood.

The analyses were carried out by reproducing the confusion matrices of each model presented in the studies, the number of cases and the prevalence of oncogene mutant positive cases. All calculations were performed on the basis of validation cohorts for studies applying a training/validation split method, or on the basis of the total sample when cross-validation was the validation strategy. To ensure homogeneity, calculations were conducted based on internal validation cohort data when external validation was also performed (minority of the cases).

Predictor factors analyzed in the meta-regression: (1) average age of the cases, (2) manual segmentation vs semi-automatic segmentation vs both procedures (no studies including automatic segmentation approaches met the inclusion criteria for the quantitative analysis), (3) whether the model included only radiomics features or was combined with clinical variables, and (4) whether the model was classified as ML or DL. The heterogeneity in the description of the clinical variables included in the models prevented the inclusion of additional predictors of greatest clinical interest. Only the best model from each study according to its DOR was selected. When the mean/median age was not available due to the heterogeneity among studies when presenting descriptive results, it was inferred from the information obtained. Thus, mean and median values were indistinctly considered; when both values were provided, an average of both was calculated. If mean values were absent, median values were considered and viceversa. If both values were absent from the validation cohort, mean/median age from the total cohort was considered. When this information was not available either, the study was not included in the meta-regression.

**References**

1. Bossuyt PM, Reitsma JB (2003) The STARD initiative. Lancet 361:71.

2. Bossuyt PM, Reitsma JB, Bruns DEet al (2003) Towards complete and accurate reporting of studies of diagnostic accuracy: the STARD initiative. Radiology 226:24-28.

3. Bossuyt PM, Reitsma JB, Bruns DEet al (2015) STARD 2015: an updated list of essential items for reporting diagnostic accuracy studies. Radiology 277:826-832.

4. Cohen JF, Korevaar DA, Altman DGet al (2016) STARD 2015 guidelines for reporting diagnostic accuracy studies: explanation and elaboration. BMJ Open 6:e012799.

5. Mongan J, Moy L, Kahn CE (2020) Checklist for artificial intelligence in medical imaging (CLAIM): a guide for authors and reviewers. Radiol Artif Intell 2:e200029.

6. Reitsma JB, Glas AS, Rutjes AWet al (2005) Bivariate analysis of sensitivity and specificity produces informative summary measures in diagnostic reviews. J Clin Epidemiol 58:982-990.
